# Novel Aporphine- and Proaporphine–Clerodane Hybrids Identified from the Barks of Taiwanese *Polyalthia longifolia* (Sonn.) Thwaites var. *pendula* with Strong Anti-DENV2 Activity

**DOI:** 10.3390/ph15101218

**Published:** 2022-09-30

**Authors:** I-Wen Lo, Geng-You Liao, Jin-Ching Lee, Chi-I Chang, Yang-Chang Wu, Yen-Yu Chen, Shang-Pin Liu, Huey-Jen Su, Chih-I Liu, Chia-Yi Kuo, Zheng-Yu Lin, Tsung-Lin Li, Yun-Sheng Lin, Chia-Ching Liaw

**Affiliations:** 1 Genomics Research Center, Academia Sinica, Taipei 115201, Taiwan; 2 Institute of Physiology, School of Medicine, National Yang Ming Chiao Tung University, Taipei 112304, Taiwan; 3 Department of Marine Biotechnology and Resources, National Sun Yat-sen University, Kaohsiung 804201, Taiwan; 4 Department of Biological Science and Technology, National Pingtung University of Science and Technology, Pingtung 912301, Taiwan; 5 Chinese Medicine Research and Development Center, China Medical University Hospital, Taichung 404332, Taiwan; 6 Graduate Institute of Integrated Medicine, College of Chinese Medicine, China Medical University, Taichung 404333, Taiwan; 7 Department of Education and Research, Taipei City Hospital, Taipei 103212, Taiwan; 8 Bachelor of Program in Scientific Agriculture, National Pingtung University of Science and Technology, Pingtung 912301, Taiwan; 9 Department of Nursing, Meiho University, Pingtung 912009, Taiwan; 10 Department of Biological Science and Technology, Meiho University, Pingtung 912009, Taiwan; 11 Division of Chinese Material Medica Development, National Research Institute of Chinese Medicine, Taipei 112026, Taiwan; 12 Department of Biochemical Science and Technology, National Chiayi University, Chiayi 600355, Taiwan

**Keywords:** *Polyalthia longifolia* var. *pendula*, clerodane, aporphine, proaporphine, anti-DENV2, NS2B-NS3 protease

## Abstract

Hybrid natural products produced via mixed biosynthetic pathways are unique and often surprise one with unexpected medicinal properties in addition to their fascinating structural complexity/diversity. In view of chemical structures, hybridization is a way of diversifying natural products usually through dimerization of two similar or dissimilar subcomponents through a C–C or N–C covalent linkage. Here, we report four structurally attractive diterpene–alkaloid conjugates polyalongarins A–D (**1**–**4**), clerodane-containing aporphine and proaporphine alkaloids, the first of its kind from the barks of Taiwanese *Polyalthia longifolia* (Sonn.) Thwaites var. *pendula*. In addition to conventional spectroscopic analysis, single crystal X-ray crystallography was employed to determine the chemical structures and stereo-configurations of **1**. Compounds **1**–**4** were subsequently subjected to in vitro antiviral examination against DENV2 by evaluating the expression level of the NS2B protein in DENV2-infected Huh-7 cells. These compounds display encouraging anti-DENV2 activity with superb EC_50_ (2.8–6.4 μM) and CC_50_ values (50.4–200 μM). The inhibitory mechanism of **1**–**4** on NS2B was further explored drawing on in-silico molecular docking analysis. Based on calculated binding affinities and predicted interactions between the functional groups of **1**–**4** and the allosteric-site residues of the DENV2 NS2B-NS3 protease, our analysis concludes that the clerodane–aporphine/proaporphine-type hybrids are novel and effective DENV NS2B-NS3 protease inhibitors.

## 1. Introduction

Dengue fever is a mosquito-transmitted infectious disease caused by dengue viruses (DENVs). DENVs belong to the genus Flavivirus in the Flaviviridae family of RNA viruses, of which a single positive-strand RNA genome can immediately translate into a single polyprotein chain by utilizing the host-cell translation machinery upon infection. This single polyprotein precursor is organized in a functionally inactive linear polyprotein NH_2_-C-prM-E-NS1-NS2A-NS2B-NS3-NS4A-NS4B-NS5-COOH in the first place [1]. Each independent and functional unit becomes mature after the polyprotein being subject to cleavage by host or self-encoded proteases. The dengue-specific vaccine, Dengvaxia, has recently been approved as a prophylactic measure, while its efficacy and the risk of developing severe syndromes in seronegative patients raise considerable concerns [2]. Dengue fever remains an incurable disease with only supportive treatment. It has been well documented that the NS2B-NS3pro protease plays a role in post-translation maturation in the viral replication. The NS3pro is an intrinsic serine protease containing a characteristic catalytic tri-ad (His51, Asp75, and Ser135) at its *N*-terminal domain; the NS2B protein is crucial in stabilizing NS3pro, thus highlighting NS2B-NS3pro as an ideal drug target concerning development of flavivirus therapeutics [3,4,5].

Natural products are born as bioactive molecules with immense structural complexity/diversity, on which hybridization can further multiply the level of complexity/diversity via permuting/recombining scaffold units at multiplex spatial dimensions. The naturally occurring hybrids are relatively rare, while their uniqueness is otherwise warranted [6,7]. Most hybrids reported thus far are microbial secondary metabolites produced via mixed biosynthetic pathways, such as NRPS and PKS pathways [6]. They are often manifested by the manner of two identical or unidentical entities coupled simply with a C–C, O–C or N–C linkage(s) by means of radical addition (diradical coupling) or carbanionic/carbocationic chemistry [8]. Their origins have now been expanded to the kingdom of plants as exemplified by identification of dimeric diterpenes longimides A and B (two bisclerodane imides) [9] and alkaloids bidebilines A–D (four bisdehydroaporphines) in *Polyalithia* [10]. *Polyalithia* spp. belongs to Annonaceae, the largest family in Magnoliales, which are deciduous or evergreen flowering trees and shrubs in tropical, mid-latitude, or temperate region [11].

**Figure 1:**
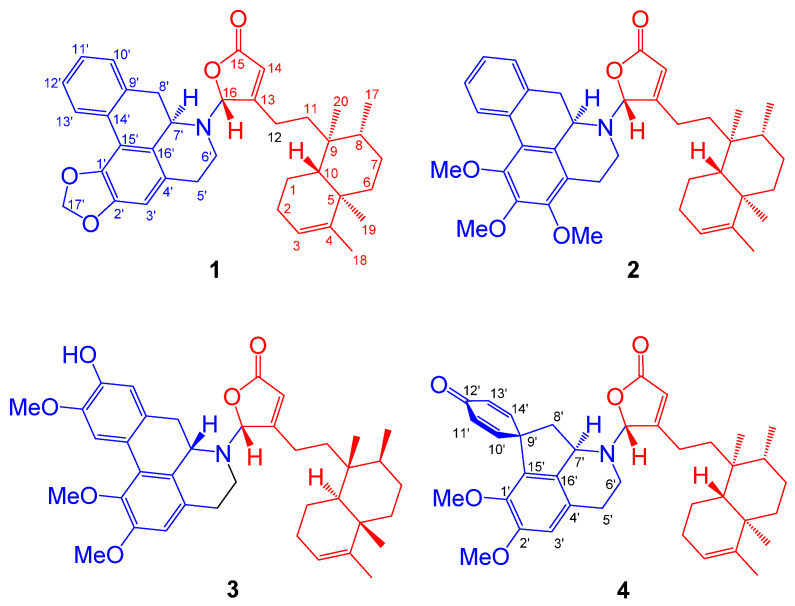
The chemical structures of polyalongarins A–D (**1**–**4**).

In this study, four novel diterpene–alkaloid hybrids named polyalongarins A–D (**1**–**4**) each assembled by one clerodane diterpene and one aporphine/proaporphine alkaloid, were isolated and identified for the first time from the barks of Taiwanese *P. longifolia* (Sonn.) Thwaites var. *pendula* (Figure 1). Both clerodane-type diterpenoids and aporphine/proaporphine-type alkaloids, individually, are abundant in *Polyalthia* (usually as ornamental trees in Southeast Asia and as a folk medicine in India) [12], which also exist in other plants, with a plethora of biological activities, such as anti-inflammation [13,14], anti-cancer [10], anti-plasmodium [15], anti-malaria [16], anti-microbes [17,18], etc. In this study, the isolated polyalongarins A–D (**1**–**4**) were examined for their biological evaluation, revealing a promising antiviral activity specific to the NS2B-NS3 protease of DENV2. The mode of action was in parallel explored with in-silico molecular docking simulation, whereby the target site of polyalongarins A–D is pinpointed at the allosteric regulation site rather than the catalytic site that does not come with the territory in this case.

## 2. Results

### 2.1. Structure Elucidation of Polyalongarins A–D (***1***–***4***)

**Figure 2:**
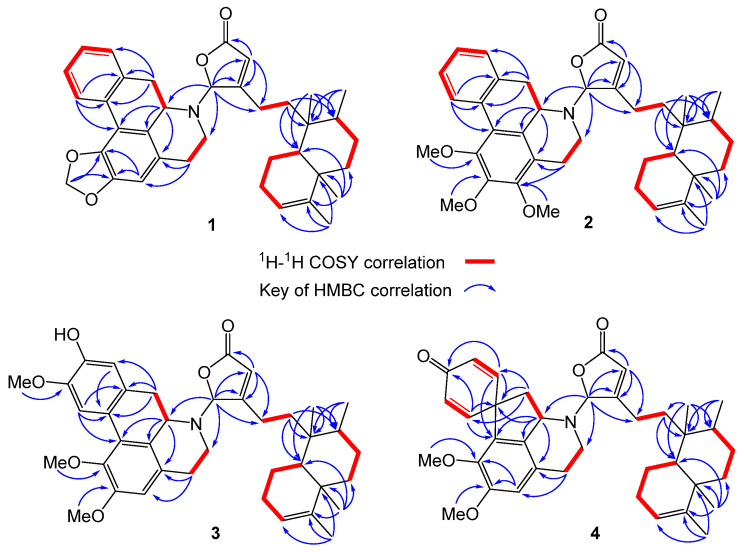
^1^H-^1^H COSY (red bold) and key HMBC (blue arrow) correlations of compounds **1**–**4**.

Polyalongarin A (**1**) was obtained as colorless crystals with specific rotation [α]${}_{D}^{25}$ = –181 (*c* 0.05, CHCl_3_). On the basis of the HRESIMS spectrum, polyalongarin A (**1**) [*m*/*z* 566.32658 [M + H]^+^ (calcd. for C_37_H_44_NO_4_, 566.32649)] complies with the “nitrogen rule”, revealing that **1** is an alkaloid possessing a molecular formula of C_37_H_43_NO_4_ with 17 degrees of unsaturation (DOU). According to ^13^C NMR and DEPTs spectra, the 37 carbons can be grouped into four methyls, ten methylenes (one nitrogen-containing methylene at δ_C_ 41.4 and one dioxymethylene at δ_C_ 100.9), four methines (one nitrogen-containing methine at δ_C_ 56.3 and one *N*,*O*-mixed acetal at δ_C_ 93.9), seven olefinic methines, two quaternary carbons, nine sp^2^ quaternary carbons, and one carboxyl group at δ_C_ 171.5. Having determined proton-carbon single bond correlations by HMQC, the planar moiety of **1** was concluded by 2D NMR (^1^H-^1^H COSY and HMBC) (Figure 2); thereby, the ^1^H- and ^13^C-NMR signals were unequivocally assigned as summarized in Table 1.

The α,β-unsaturated γ-lactone unit was confirmed based on the HMBC correlations for H-14 (δ_H_ 5.98 s) and H-16 (δ_H_ 6.19 s) extended to C-13 (δ_C_ 169.8), C-15 (δ_C_ 171.5), and C-16 (δ_C_ 93.9), where the characteristic chemical shift value at δ_C_ 93.9 (C-16) stands for a strong electron-withdrawing group adjacent to the γ-position of the five-membered ring. Next, a tetramethyl-octahydronaphthalene bicyclic ring (a decalin-derivative) was established by two ^1^H-^1^H COSY correlations of H-10/H_2_-1/H_2_-2/H-3 and H_2_-6/H_2_-7/H-8/CH_3_-17 alongside the HMBC correlations for the four methyls to their neighbor carbons (Figure 2): CH_3_-17 (δ_H_ 0.79 d, *J* = 4.5) to C-7, C-8, and C-9; CH_3_-18 (δ_H_ 1.58 s) to C-3, C-4, and C-5; CH_3_-19 (δ_H_ 1.01 s) to C-4, C-5, C-6, and C-10; and CH_3_-20 (δ_H_ 0.78 s) to C-8, C-9, C-10. Given the HMBC cross peaks of H-14/C-12, H-16/C-12, and CH_3_-20/C-11 and the ^1^H-^1^H COSY correlations of H_2_-11/H_2_-12, the α,β-unsaturated γ-lactone ring is determined in connection with the tetramethyl-octahydronaphthalene bicyclic ring through a short C2 chain known as a type III clerodane diterpenoid [19]. On the other hand, the remaining twelve olefinic carbons and eleven DOU suggest that the alkaloid portion is made of a five-membered ring structure and two benzene rings. Three correlations of H-5′/H-6′, H-7′/H-8′, and H-10′/H-11′/H-12′/H-13′ were observed in the COSY spectrum of **1**, as well as the long range ^1^H-^13^C correlations of H-3′/C-1′, C-2′, C-4′, C-5′, C-16′; H-5′/C-3′, C-4′; H-7′/C-4′, C-6′, C-15′, C-16′; H-8′/C-9′, C-10′, C-14′, C-16′; H-10′/C-8′, C-9′, C-14′; H-13′/C-9′, C-14′, C-15′; and H_2_-17/C-1′ and C-2′ were deduced from the HMBC spectrum. Taken together, a benzylisoquinoline named anonaine [17,18] was determined. Moreover, an unexpected but crucial HMBC correlation of H-16/C-6′, C-7′ was observed, where the strong electron-pulled phenomenon at C-16 resulted from the *N* atom of anonaine and the carboxyl group. The C-N bond that connects the clerodane and the anonaine thereby brings about the unprecedented hybrid of clerodane–aprophine alkaloid, named polyalongarin A (**1**) here.

**Figure 3:**
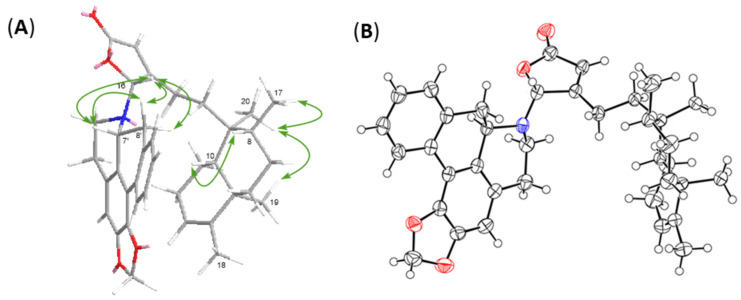
Main NOESY correlations (**A**) and ORTEP diagram (**B**) of compound **1**.

Given that there are four intriguing but dizzy diastereomers *trans–cis* (TC), *trans–trans* (TT), *cis–cis* (CC), and *cis–trans* (CT) in the decalin system, the neo- or *ent*-neo-absolute configurations of clerodanes were determined on the basis of the stereo-configurations of C-5, C-8, C-9, and C-10 [20]. In brief, the absence of NOESY correlations for H-10/CH_3_-19 but the appearance of that for H-10/H-8 and CH_3_-17/CH_3_-19/CH_3_-20 supports that the clerodane skeleton in compound **1** is a type of TC. The C-7′ in anonaine is usually a *R*-configuration with a negative optical rotation as seen in previous reports, while the chirality of C-16 is uncertain merely based on the NOESY correlations (Figure 3A) of H-16/H-7′/H-8′α and H-16/H-8′β. To resolve this uncertainty, we resorted to X-ray crystallography. The crystals of **1** were grown in a MeOH/CH_2_Cl_2_ solvent system and subsequently subjected to X-ray diffraction, whereby the crystal structure undoubtedly depicts the absolute stereochemistry of **1**. The ORTEP diagram for the crystal structure of **1** (Figure 3B) confirms the TC type configuration of clerodane (neo-TC), namely, the *R*-configuration of C-16 and the *P* helical conformation between the five-membered γ-lactone ring and aprophine.

The framework of polyalongarin B (**2**) is akin to that of 1, in that both share similar UV, IR, NMR, optical rotation and circular dichroism (CD) spectra. Its molecular formula is C_39_H_49_NO_5_ with 16 DOU according to HRESIMS [*m*/*z* 612.36844 [M + H]^+^ (calcd. for C_39_H_50_NO_5_, 612.36835)]. There are three additional aliphatic methoxy groups with chemical shifts at δ_H_ 3.95 (3H, s), 3.70 (3H, s), and 3.92 (3H, s) in ^1^H-NMR spectrum (Table 1), whereas the absence of dioxymethylene is observed from ^13^C-NMR spectrum (Table 1). In conjunction with HMBC correlations of H_2_-5′/C-3′ and C-4′; 1′-OMe/C-1′; 2′-OMe/C-2′; and 3′-OMe/C-3′ (Figure 2), 2 was determined to be a clerodane-containing 1′,2′,3′-trimethoxy-aprophine alkaloid similar with **1**.

**Figure 4:**
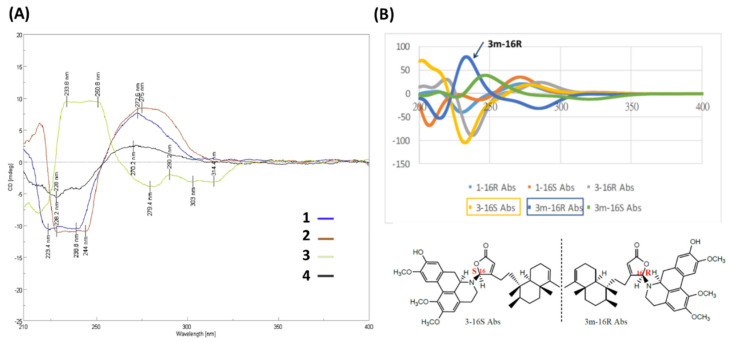
Stereochemistry of **1**–**4** determined by CD/ECD. (**A**) Experimental CD spectra of **1**–**4**. (**B**) Calculated ECD comparison between **1** and **3**. The calculated ECD curves of different stereoisomers of **1** and **3** in B are shown as **1**-16*R*, light blue; **1**-16*S*, orange; **3**-16*R*, gray; **3**-16*S*, yellow orange; **3**-16*R*, gray; **3**-16*S*, yellow orange; **3m**-16*R* (the mirror isomer of **3** with 16*R*), blue; **3m**-16*S* (the mirror isomer of **3** with 16*S*), green.

Based on spectroscopic analysis, **3** is likely a clerodane–aprophine alkaloid. As a matter of fact, it is a hydroxylated analog of **2** with molecular formula of C_39_H_49_NO_6_ (16 DOU) derived from HRESIMS [*m*/*z* 628.36295 [M + H]^+^ (calcd. for C_39_H_50_NO_6_, 628.32326)]. Given the absence of ^1^H-^1^H COSY fragment of H-10′/H-11′/H-12′/H-13′ and atypical HMBC correlations (H-10′/C-9′, C-11′, C-12′, and C-14′; H-13′/C-9′, C-11′, C-12′, and C-14′; 1′-OMe/C-1′; 2′-OMe/C-2′; and 12′-OMe/C-12′), the chemical entity **3** is determined to be a 1′,2′,12′-trimethoxy-11′-hydroxy-aprophine alkaloid. Given the optical rotation [35.4 (*c* 0.05, CHCl_3_)] and the reverse CD curve (Figure 4A), 3 is clearly distinct from others [21]. Taking advantage of MMFF94 energy minimization in the Chem3D 16.0 suite [22], the dihedral angle ω_16-N,C-16,C-13,C-12_ of **3** should be the same no matter the lone pair on the 16-*N* atom pointing toward the α or β direction. However, the helical conformation of the γ-lactone ring and aprophine is poised in an *M*-configuration (Figure S1 in the Supporting Information). In the ECD calculation for all possible stereoisomers of **3** (Figure 4B), only is **3m**-16*R* (the mirror isomer of **3** with 16*R*) in line with the experimental CD spectrum of **3** concerning the binary positive [π→π* transitions at 234 and 251 nm] and negative [n→π* transitions at 279 and 303 nm] Cotton effects. Altogether, the most plausible configuration for 3 is 16*R*, 7’*S*, *ent*-neo-TC, not least considering both the NOESY correlations and the absence of NOESY correlations of H-16/H-6′β/H-7′.

Similarly, polyalongarin D (**4**) was first analyzed by HRESIMS with *m*/*z* 598.35281 [M + H]^+^ (calcd. for C_38_H_49_NO_5_, 598.35270). On the basis of ^1^H- and ^13^C NMR (Table 1), four methyls at δ_H_ 0.75 (d, *J* = 5.5 Hz), 0.77 (s), 1.00 (s), 1.57 (s), and two sp^2^ methines at δ_C_ 5.16 (br s) and 5.98 (s) were spotted; four olefinic carbons at δ_C_ 118.6, 120.1, 144.6, 169.0, and one carboxyl carbon at δ_C_ 171.4 were additionally identified, together suggesting that **4** is a cleordane–alkaloid congener with a molecular formula of C_38_H_47_NO_5_ and 16 DOU. Apart from the signals for a typical clerodane, a characteristic AA′XX′ proton system δ_H_ 6.90 (d, *J* = 10.0 Hz), 6.33 (d, *J* = 10.0 Hz), 6.44 (d, *J* = 10.0 Hz), and 7.04 (d, *J* = 10.0 Hz) at the down field indicates two pairs of aromatic ortho-protons, which are further ascertained by HMBC correlations [H-9′ (H-13′)/C-8′, C-10′ (C-12′), C-11′, C-13′ (C-9′), C-14′, and C-15′; and H-10′ (H-12′)/C-9′ (C-13′), C-11′, C-12′ (C-10′) and C-14′]. All together establish a unique spiro conformation. On the other hand, specific sets of cross peaks (H-3′/C-1′, C-2′, C-4′, C-5′, C-16′; H-8′/C-9′, C-13′, C-14′, C-15′, C-16′; 1′-OMe/C-1′; 2′-OMe/C-2′) identified from HMBC spectrum are elucidated, which correlates the alkaloid to a stepharine moiety [23]. Moreover, the optical rotation [–61.0 (*c* 0.05, CHCl_3_)] of **4** echoes a similar CD curve in analogy to those of **1** and **2**, suggesting the stereochemistry of **4** follows 5*R*, 8*R*, 9*S*, 10*R*, 16*R*, and 7′*R*. Summing up, compound **4** is a new type of hybrid featuring an unusual chemical architecture of a clerodane–proaporphine alkaloid.

The biosynthetic pathways for these hybrids **1**-**4** are put forward and shown in Figure S2 in the Supporting Information.

### 2.2. Anti-DENV2 Activities of Polyalongarins A–D (***1***–***4***)

**Figure 5:**
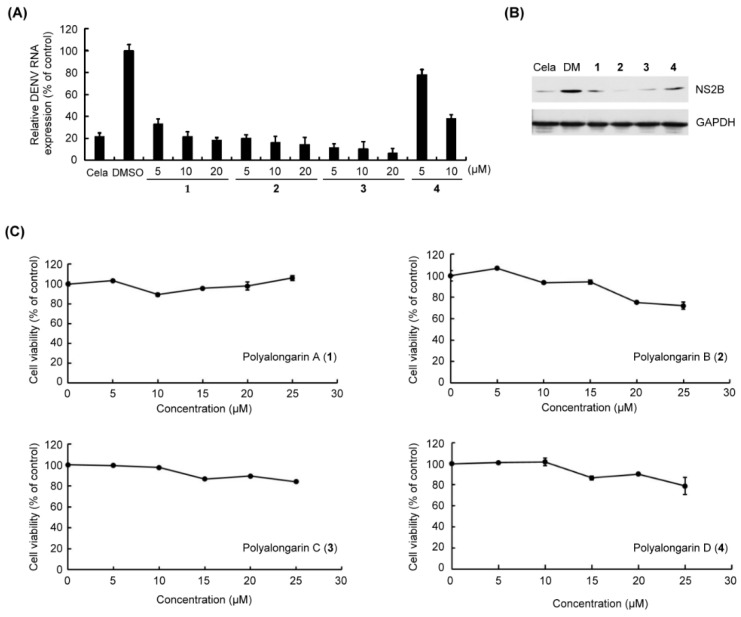
In vitro anti-DENV2 assay for **1**–**4**. (**A**) DENV2-infected/uninfected Huh-7 cells were incubated with celastrol (Cela, 0.2 μM, positive control) or indicated concentrations of **1**–**4** (5 to 25 μM) for 72 h. Total DENV2 RNA expression (%) was extracted and quantified. The DENV2 RNA level was measured by RT-qPCR. The DENV2 RNA expression was normalized by cellular GAPDH mRNA. (**B**) DENV2-infected Huh-7 cells were treated with 10 μM of **1**–**4**. The NS2B protein expression was analyzed by western blotting with anti-NS2B and anti-GAPDH antibodies (a loading control). (**C**) The cell viability (%) was determined by the XTT method. Treatment with 0.1% DMSO served as a mock control. The results are expressed as the means ± standard errors of the means (SEM) of triplicate experiments.

Concerning their biological activity, the anti-DENV2 activity of polyalongarins A–D (**1**–**4**) was examined using post-infection assay. The DENV2-infected Huh-7 cells were incubated with each of **1**–**4** at a concentration of 5, 10, or 20 μM for 72 hrs (0.2 μM celastrol and 0.1% DMSO served as positive control and mock control, respectively) [24]. It was defined active when a compound exhibited >50% inhibition of DENV at a given concentration specified above. All compounds **1**–**4** showed >50% inhibition of DENV2 RNA expression within the testing range (Figure 5), and the IC_50_ values for **1**–**4** were, respectively, determined to be 4.46, 2.80, 3.24, and 6.36 μM with sorted inhibitory effect in order as **2** > **3** > **1** > **4** in agreement with the western blotting analysis. In the meantime, the uninfected Huh-7 cells were incubated with each individual **1**–**4** at a range of concentrations for cytotoxicity evaluation, of which the result suggests no or very low cytotoxicity (Table 2). Based on previous reports [25], a compound that diminishes the DENV activity by 90% but presents a 50% cytotoxic concentration (CC_50_) > 15 μM is considered a “hit”. By this definition, compounds **1**–**4** are good hits without doubt.

### 2.3. Molecular Docking Analysis of Polyalongarins and DENV2 NS2B-NS3pro

**Figure 6:**
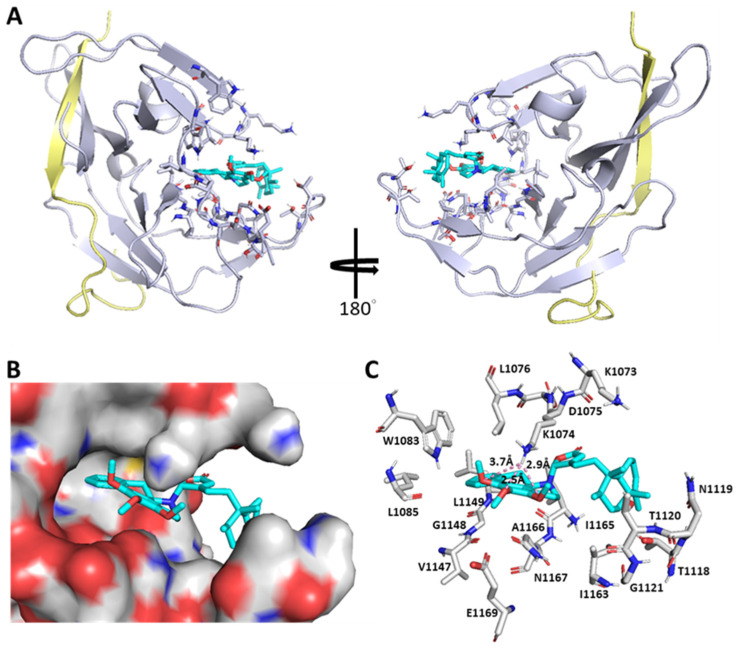
Computational modeling for polyalongarin B (**2**) bound to the allosteric site of NS2B-NS3pro. (**A**) DENV NS2B-NS3pro in complex with **2**. Gray, NS3pro; yellow, NS2B. (**B**) Compound **2** is located in the pocket of the allosteric site (shown in electrostatic surface). (**C**) Compound **2** interacts with the allosteric-site residues. Cyan, compound **2**. The default analytical distance for the ligand within its binding site is set less than 5 Å. Figures are prepared using PyMOL 2.0 software (*Warren L. DeLano*, PyMOL 2.0 software; Schrödinger, Inc. 2017).

The NS2B-NS3 protease is essential for flavivirus infections and their sequences are highly conserved (Figure S3 in the Supporting Information), highlighting the NS2B-NS3pro protease as an ideal antiviral target. To date, only a handful of apo/liganded structures of DENV NS2-NS3 proteases from the PDB database (Figure S4 in the Supporting Information) are available, indicating their difficulty in crystallization. For the docking study, compounds **1**–**4** were individually modeled into DENV2 NS2B-NS3pro (PDB ID: 6MO1) by using AutoDock 4.2 and AutoDock Vina under the PyRx suite for probing the most possible binding site and evaluating their interactions with NS2B-NS3pro. To our surprise, compound **2**, for instance, neither stays in the anticipated NS3pro active site (H51, D75, and S135) nor interacts with NS2B (residues 48–71) as shown in Figure 6. Instead, it enters into an unexpected binding pocket constituted by residues (Met1049–Asn1167), wherein Lys1074 adapts double conformations in cooperation with the “open–active” or “closed–inactive” form of NS2B-NS3pro. Namely, the ε-NH_2_ group of Lys1074 is in close proximity to three *O* atoms of 1′,2′,3′-trimethoxyl groups of **2** through a constellation of H-bonds in the closed-inactive model, thereby revealing that **2** is an allosteric inhibitor specific to the DENV2 NS2B-NS3 protease. In addition to **2**, the other clerodane–aporphine/proaporphine hybrids also display comparably as allosteric regulators of the NS2B-NS3 protease (Figure S5 in the Supporting Information) with an overall high anti-DENV2 activity but no observable cytotoxicity.

## 3. Discussion

Given plentiful examples on opium alkaloids and clerodane-type diterpenoids in literature, a plausible biosynthetic pathway for aporphine/proaporphine alkaloids and clerodanes can be proposed, in which hybrid products **1**–**4** are joined with a C–N bond that may be achieved by a 1,2-dehydroreticuline reductase (DRR) homologue-mediated nucleophilic addition reaction (Figure S2 in the Supporting Information). Such a coupling reaction is reminiscent of the biosynthesis of imidazoyl hybrid conidiogenone B, where an α,β-hydrolase Con-ABH puts an imidazole-containing alkaloid (meleagrin) and a diterpenoid (conidiogenone) together via an aza-Michael addition reaction [26]. The isomerization from *S-* to *R*-configuration of the chiral center C7′ in the coclaurine alkaloid intermediate is likely achievable by recruiting two enzymes, 1,2-dehydroreticuline synthase (DRS) and DRR [27,28], whereby clerodanes may be subject to structural reconfiguration into a normal (chair–chair–“normal”), *ent* (chair–chair–“antipodal”), *syn* (chair–boat–“normal”), or *syn-ent* (chair–boat–“antipodal”) conformation [20,29]. In bioactive evaluation, compounds **1**–**4** showed strong anti-DENV2 activity (EC_50_ values: 2.8–6.4 μM and CC_50_ values: 50.4–200 μM) as a result of suppressing NS2B-NS3pro expression. In molecular docking analysis (polyalongarins vs. DENV2 NS2B-NS3pro), the simulation reveals that these four ligands do not hold the catalytic triad (H51, D75, and S135) at bay but instated dock at the cave lined by specific residues (M1049, H1051, W1063, K1073, K1074, D1075, L1076, T1120, G1148, L1149, G1151, I1165, A1166, and N1167) (Figure 6), suggesting that polyalongarins are potent allosteric regulators inhibiting the protease activity of DENV2 NS2B-NS3. Lys1074 and Asn1167 residues are the allosteric domain of pocket in NS2B-NS3pro and critical for its activity [4,30]. Also, it has also been reported that aminopyrazine derivatives could bind to the pocket of NS2B-NS3pro and affects its activity [4].

The anti-DENV2 assay and the in silico analysis are in a position to gauge the order of inhibitory strength in **2** > **3** > **1** > **4**. Based on their structure–activity–relationship (NS2B-NS3pro and **1**–**4**), Lys1074, Leu1149, and Asn1167 are highlighted as particularly critical and potent for the allosteric regulation. Three OMe groups in aprophine of **2** may make **2,** getting a little closer to Lys1074. In aprophine of **3**, the additional 11′-OH and the absence of 3′-OMe may together usher this ligand to lean toward Asn1167. In contrast, the dioxomethylene substitution of **1** and the bulky spiro moiety of **4** may prohibit their interactions with Lys1074, thus resulting in relatively poor NS2B inhibitory activities (Figure S5). Together, our results indicate that the inhibitory target of compounds **1**–**4** is the NS2B-NS3 protease. The activity of NS2B-NS3pro has been reported to be critical for the replication of DENV [31] and therefore an attractive target for the development of anti-dengue drugs [32]. Here, we provide evidence that clerodane–aporphine/proaporphine-type hybrids isolated from the barks of *P. longifolia* var. *pendula* are unique and potent DENV NS2B-NS3 protease inhibitors favorabe for developing into anti-dengue agents.

## 4. Materials and Methods

### 4.1. General

In structural elucidation, the optical rotations were determined using a JASCO P-1020 polarimeter (Jasco Co., Tokyo, Japan); the ultraviolet spectra (UV) were recorded on a JASCO V530 UV/VIS spectrophotometer; the circular dichroism spectroscopy (CD) were recorded on a JASCO J-815 spectrophotometer; the infrared spectra (IR) were recorded on a Mattson Genesis II FT-IR spectrophotometer (Thermo Nicolet, Madison, WI, USA); electrospray ionization mass (ESIMS) and high-resolution ESIMS data were measured using a VG Biotech Quattro 5022 mass spectrometer (VG Biotech, UK); nuclear magnetic resonance (NMR) spectra were collected on a Varian Unity Inova-400 MHz NMR system; the single crystal X-ray diffraction of pure compounds was determined by Bruker APEX II X-ray single crystal diffractometer. In chemical isolation and separation, column chromatography (CC) was applied including silica gel 60 (70–230 and 230–400 mesh, Merck, Darmstadt, Germany), Sephadex LH-20 (Pharmacia), and pre-coated silica gel TLC plates (aluminum Merck Silica gel 60 F_254_). HPLC purification was performed on a Hitachi L-2,130 Intelligent pump with a Hitachi L2420 UV-Vis detector, equipped with a 250 × 20 mm i.d. preparative Ascentis Si (5 μm) and Purospher STAR RP-18 (5 μm). In biological evaluation, cell viability/cytotoxicity assay was evaluated using CellTiter 96 AQueous One Solution Cell Proliferation Assay (Promega, Madison, WI, USA) according to the manufacturer’s instructions, where the color intensity was detected at 490 nm using a 550 BioRad plate-reader for determining the number of viable cells in proliferation. Computational modeling analyzed by PyMOL 2.0 software (creator is Warren L. DeLano; published by Schrödinger, Inc. 2017; and the latest vision is PyMOL 2.5 vision).

### 4.2. Plant Material

The barks of *Polyalthia longifolia* (Sonn.) Thwaites var. *pendula* (synonym: *Monoon longifolium* var. *pendula*) were collected from Wanluan township, Pingtung County, Taiwan, in July 2015, and identified by one of the authors (Y.-S. Lin). A voucher specimen (No. ME2015-BST01) has been deposited in the Department of Biological Science and Technology, Meiho University, Taiwan.

### 4.3. Extraction and Isolation

The freeze-drying barks of *P. longifolia* var. *pendula* (9.5 kg) were grinded and extracted with 20 L of methanol (MeOH) at room temperature three times, and the combined extracts were concentrated in vacuum. The combined MeOH extract (310 g) was suspended into 3% aqueous solution of tartaric acid, and then partitioned with ethyl acetate (EtOAc). After adjusting the acidic solution to pH 9 by saturated Na_2_CO_3_ solution, the mixture was further partitioned with CH_2_Cl_2_. The CH_2_Cl_2_ extract was washed with double distilled water and concentrated in reduced pressure to obtain an alkaloid-containing extract (72 g). This crude alkaloid extract was partitioned between *n*-hexane and 75% MeOH aqueous (1:1) to afford a n-hexane layer (39 g) and a MeOH (aq.) layer (33 g). The MeOH layer was subjected to silica gel flash chromatography with a *n*-hexane/EtOAc solvent system (1:0, 20:1, 10:1, 8:1, 6:1, 5:1, 4:1, 3:1, 2:1, 1:1, 0:1), and ten fractions (PL1~PL10) were obtained. The PL2 fraction (3.2 g) was further separated by an open column prepared by silica gel to give five sub-fractions (PL2-1~PL2-5). The sub-fraction PL2-2 was re-suspended for crystallization with MeOH/CH_2_Cl_2_ solution, and spindle crystals were obtained as polyalongarin A (**1**, 16.1 mg). The sub-fraction PL2-3 was successively subjected to normal-phase HPLC (eluting with *n*-hexane/EtOAc = 5:1) and preparative TLC (eluting with *n*-hexane/acetone = 5:2) to purify polyalongarin B (**2**, 11.1 mg). The sub-fraction PL2-4 (2.1 g) was chromatographed by a Sephadex LH-20 column with elution of CH_2_Cl_2_/MeOH (1:1), and seven fractional fractions (PL2-4-1~PL2-4-7) were given. Of them, polyalongarin D (**4**, 4.7 mg) was isolated after purification of PL2-4-3 (480 mg) via reverse-phase C_18_ HPLC (MeOH/H_2_O/acetonitrile = 55:40:5). Finally, the sub-fraction PL2-6 (4.9 g) was fractionated by using Sephadex LH-20 column chromatography, which was eluted with CH_2_Cl_2_/MeOH (1:1) and then further purified by normal-phase HPLC with *n*-hexane/EtOAc (2:1) to obtained polyalongarin C (**3**, 13.1 mg).

### 4.4. Spectral Measurements

Polyalongarin A (**1**): Colorless needle crystal; [α]${}_{D}^{25}$ −181.0 (*c* 0.05, CHCl_3_); UV (CDCl_3_) λ_max_ (log ε) 273 (3.41), 225 (3.93) nm; IR (KBr) *ν*_max_ 2939, 2860, 1755, 1447, 1252, 1220, 1139, 1047 cm^−1^; ^1^H-NMR (500 MHz, CDCl_3_) and ^13^C-NMR (125 MHz, CDCl_3_) spectroscopic data, see Table 1; ESIMS *m*/*z* 566 [M + H]^+^; HRESIMS *m*/*z* 566.32658 [M + H]^+^ (calcd. for C_37_H_44_NO_4_, 566.32649).

Polyalongarin B (**2**): White amorphous powder; [α]${}_{D}^{25}$ −36.0 (*c* 0.05, CHCl_3_); UV (CDCl_3_) λ_max_ (log ε) 273 (1.042), 213 (3.41) nm; IR (KBr) *ν*_max_ 3346, 2362, 2296, 1639, 1267, 1055, 1014 cm^−1^; ^1^H-NMR (500 MHz, CDCl_3_) and ^13^C-NMR (125 MHz, CDCl_3_) spectroscopic data, see Table 1; ESIMS *m*/*z* 612 [M + H]^+^; HRESIMS *m*/*z* 612.36844 [M + H]^+^ (calcd. for C_39_H_50_NO_5_, 612.36835).

Polyalongarin C (**3**): White amorphous powder; [α]${}_{D}^{25}$ +35.4 (*c* 0.05, CHCl_3_); UV (CDCl_3_) λ_max_ (log ε) 302 (3.09), 281 (3.14), 217 (3.60) nm; IR (KBr) *ν*_max_ 3349, 2959, 2362, 1740, 1644, 1251, 1135, 1068 cm^−1^; ^1^H-NMR (500 MHz, CDCl_3_) and ^13^C-NMR (125 MHz, CDCl_3_) spectroscopic data, see Table 1; ESIMS *m*/*z* 628 [M + H]^+^; HRESIMS *m*/*z* 628.36295 [M + H]^+^ (calcd. for C_39_H_50_NO_6_, 628.32326).

Polyalongarin D (**4**): White amorphous powder; [α]${}_{D}^{25}$ −61.0 (*c* 0.05, CHCl_3_); UV (CDCl_3_) λ_max_ (log ε) 285 (2.63), 234 (3.34), 213 (3.72); IR (KBr) *ν*_max_ 3736, 3388, 2929, 2355, 2285, 1747, 1657, 1540, 1089 cm^−1^; ^1^H-NMR (500 MHz, CDCl_3_) and ^13^C-NMR (125 MHz, CDCl_3_) spectroscopic data, see Table 1; ESIMS *m*/*z* 598 [M + H]^+^; HRESIMS *m*/*z* 598.35281 [M + H]^+^ (calcd. for C_38_H_49_NO_5_, 598.35270).

### 4.5. X-ray Crystallographic Data of Polyalongarin A (***1***)

A colorless crystal of polyalongarin A (**1**) was obtained by simple evaporation from CH_2_Cl_2_/MeOH solution. The data was collected on a Bruker APEX II X-ray single crystal diffractometer with MoKα radiation (λ = 1.54178 Å) at 200 K in the Instrumentation Center of National Taiwan Normal University, Taiwan. Crystal data: crystal size 0.20 × 0.11 × 0.05 mm^3^, C_37_H_43_NO_4_, monoclinic, a = 12.2259(2) Å, b = 9.50360(10) Å, c = 13.0577(2) Å, V = 1494.23(4) Å^3^, space group P21, *Z* = 4, density (calculated) 1.257 Mg/m^3^, absorption coefficient 0.635 mm^−1^, F(000) = 608, and 1761 reflections were collected for crystal structural analysis by 5242 independent reflections [R_int_ = 0.0485] with *I* > 2*σ* (*I*). The data were solved by using the “direct method” (normal model), and the chemical structure was refined by the “full-matrix least-squares” procedure on *F*^2^ values. All non-hydrogen atoms were refined anisotropically within thermal parameters, and the hydrogen atom positions were idealized geometrically and attached to ride on the parent carbon atoms with C–H distances (0.98–1.00 Å). The final indices (I > 2σ(I)) were *R*1 = 0.0383, *wR*2 = 0.0942 with goodness-of-fit = 1.044. The absolute structure parameter is -002(17). Crystal data and structure refinement are shown on Table S1 in the Supporting Information. The final X-ray model is shown in Figure 3. The crystal data of polyalongarin A (**1**) have been deposited in the Cambridge Crystallographic Data Centre, CCDC number: 1939242.

### 4.6. Cytotoxic Assay

Huh-7 cells were seeded in 96-well plates at a density 5 × 10^4^ cells/well in 120 μL of culture medium and incubated for 6 hr in a humidified CO_2_ (5%) atmosphere. Then, respectively, treated Huh-7 cells with 0.1% DMSO (positive control) and analytes at indicated concentrations, followed by incubation at 37 °C for an additional 72 hr. Then, the XTT method was applied to cell viability evaluation: cells were dyed by 2,3-bis(2-methyloxy-4-nitro-5-sulfophenyl)-2*H*-tetrazolium-5-carboxanilide and recorded by an enzyme-linked immunosorbent assay (ELISA) plate reader (at UV 490 nm). Three independent experiments were performed at each treatment concentration, and all data are presented as mean ± standard error of the mean (SEM).

### 4.7. In Vitro Anti-DENV2 Assay

Huh-7 human liver carcinoma cell line, purchased from Bioresources Collection and Research Center, Taiwan, were maintained in Dulbecco’s modified Eagle’s medium (DMEM) supplemented with 10% fetal bovine serum (FBS), 1% non-essential amino acids (NEAA), and 1% penicillin and streptomycin at 37 °C in a humidified 5% CO_2_ incubator. The DENV serotype 2 (strain 16681) was directly from the Centers for Disease Control, Department of Health, Taiwan, and was amplified in Aedes albopictus C6/36 cell line (CRL-1660, ATCC, USA). C6/36 cells were cultured in RPMI 1640 medium supplemented with 2% FBS. Huh-7 cells were seeded at a density of 5 × 10^4^ cells/mL in the 24-well plate for 16–20 h and then infected with DENV2 at a multiplicity of infection (MOI) of 0.1 PFU/cell for 2 h at 37 °C. The viral titer was determined in a plaque assay in the Huh-7 cells. Then, DENV2-infected Huh-7 cells were washed with PBS and refilled, respectively, with DMEM−2% FBS medium containing 2 μM celastrol (positive control), 0.1% DMSO (mock control), and various indicating concentration of test compounds. After incubation at 37 °C for 72 h, DENV2-infected Huh-7 cells were harvested and cell lysates were prepared for quantification of DENV RNA by RT-qPCR. Total cellular RNA was extracted using Total RNA Miniprep Purification Kit (GMbiolab, Taiwan) according to the manufacturer’s instructions.

### 4.8. Western Blotting Analysis

Cell lysates (20 μg) were loaded onto and assessed by SDS-PAGE, and transferred to an electroblotting PVDF membrane that had been incubated overnight with rabbit polyclonal antibodies against NS2B (1:3,000; GeneTex, Irvine, CA, USA) and anti-GAPDH antibodies (1:10,000; GeneTex, Irvine, CA, USA) serving as loading control.

### 4.9. In Silico Molecular Docking by PyRx

The molecular docking studies were performed by using PyRx 0.8 software [33], a suite of established open-source function including: AutoDock 4.2 and AutoDock Vina [34] are used as a docking software; AutoDockTools is used to generate input files; whereas Open Babel is used for importing SDF files, removing salts and energy minimization. AutoDock 4.2, AutoDock vina, and AutoDock Tools 1.5.7 are created by the Center for Computational Structural Biology in the Scripps Research Institute. Prior to molecular docking, the protein molecules were prepared by using a standard protocol for the structural preparation tool of AutoDock 4.2 that included addition of hydrogen atoms and assignment of charges (Compute Gasteiger–Marsili:Ligand). AutoDock 4.2 and AutoDock Vina, implementing a Lamarckian Genetic Algorithm (LGA), were employed for molecular docking studies. AutoDock Tools 1.5.7 was used to generate a grid box around the active site with other parameters set as default in the input PDBQT files of protein. All the docking conformations were clustered together ground on RMSD, and the one with the most favorable free energy or the highest percentage frequency was selected as representative. 3D coordinates of ligands, polyalongarins A–D (**1**–**4**), were obtained using Open Babel for MMFF94 energy minimization. The dataset of compounds **1**–**4** were subjected to structure-based virtual screening against the selected protein, DENV2 NS2B-NS3 protease, and the protein was kept rigid during docking calculation. Finally, the virtually screened best conformation poses and binding affinities of each ligands were ensured (Tables S3 and S4 in the Supporting Information), and the interactions between protein and ligands were visualized and illustrated using PyMOL 2.0 software (Figure S5 in the Supporting Information). In these computational simulations of protein–ligand interactions, the defaulted distance of ligand within the active site is less than 5Å; protein is presented in a cartoon or hydrophobicity surface model; the residues of active site and ligands are drawn in sticks.

## 5. Conclusions

In this study, we presented four hybrid polyalongarins A–D (**1**–**4**) isolated from the barks of Taiwanese *P. longifolia* (Sonn.) Thwaites var. *pendula* which exhibited amazing inhibitory activity against DENV2 with EC_50_ ranging from 2.8–6.4 μM and CC_50_ ranging from 50.4–200 μM. The inhibitory target of **1**–**4** was revealed to be NS2B by proteomic analysis. On the basis of in silico simulation, estimated binding affinities and predicted interactions between the allosteric-site residues of the DENV2 NS2B-NS3 protease (PDB ID: 6MO1) and compounds **1**–**4**, we conclude that the clerodane–aporphine/proaporphine type hybrids are unique and potent DENV NS2B-NS3 protease inhibitors suitable for developing into anti-Dengue agents.

## Figures and Tables

**Table 1:** ^1^H- and ^13^C- NMR spectroscopic data of Polyalongarin A-D (**1**–**4** in CDCl^3^).

No	Polyalongarin A (1)		Polyalongarin B (2)		Polyalongarin C (3)		Polyalongarin D (4)	
	^1^H (mult, Hz)	^13^C (mult.)	^1^H (mult, Hz)	^13^C (mult.)	^1^H (mult, Hz)	^13^C (mult.)	^1^H (mult, Hz)	^13^C (mult.)
1	1.52 (m, 2H)	18.4 (CH_2_)	1.54 (m, 2H)	18.4 (CH_2_)	1.52 (m, 2H)	18.2 (CH_2_)	1.48 (m, 2H)	18.3 (CH_2_)
2	1.98 (m) 2.10 (m)	26.9 (CH_2_)	2.00 (m) 2.10 (m)	26.9 (CH_2_)	1.95 (m, 2H)	26.7 (CH_2_)	1.86 (m) 2.03 (m)	26.9 (CH_2_)
3	5.18 (brs)	120.2 (CH)	5.17 (brs)	120.2 (CH)	5.14 (brs)	120.3 (CH)	5.16 (brs)	120.1 (CH)
4		144.4 (C)		144.4 (C)		144.3 (C)		144.6 (C)
5		38.1 (C)		38.1 (C)		38.1 (C)		38.2 (C)
6	1.20 (m) 1.73 (m)	36.6 (CH_2_)	1.20 (m) 1.73 (m)	36.5 (CH_2_)	1.19 (m) 1.72 (m)	36.6 (CH_2_)	1.14 (m) 1.71 (m)	36.6 (CH_2_)
7	1.37 (m) 1.44 (m)	27.3 (CH_2_)	1.45 (m, 2H)	27.3 (CH_2_)	1.42 (m) 1.44 (m)	27.3 (CH_2_)	1.42 (m, 2H)	27.2 (CH_2_)
8	1.45 (m)	36.4 (CH)	1.47 (m)	36.4 (CH)	1.50 (m)	36.4 (CH)	1.40 (m)	36.4 (CH)
9		38.7 (C)		38.6 (C)		38.7 (C)		38.6 (C)
10	1.36 (dd, 10.5, 2.0)	46.5 (CH)	1.36 (dd, 10.5, 2.0)	46.6 (CH)	1.32 (m)	46.4 (CH)	1.31 (m)	46.4 (CH)
11	1.62 (m) 1.70 (m)	35.0 (CH_2_)	1.63 (m)	34.8 (CH_2_)	1.53 (m) 1.79 (m)	35.2 (CH_2_)	1.58 (m) 1.68 (m)	34.7 (CH_2_)
			1.67 (m)					
12	2.25 (m, 2H)	22.2 (CH_2_)	2.23 (m, 2H)	22.1 (CH_2_)	2.12 (m) 2.30 (m)	22.0 (CH_2_)	2.05 (m, 2H)	21.9 (CH_2_)
13		169.8 (C)		169.8 (C)		169.9 (C)		169.0 (C)
14	5.98 (s)	118.7 (CH)	5.98 (s)	118.5 (CH)	5.98 (s)	118.7 (CH)	5.98 (s)	118.6 (CH)
15		171.5 (C)		171.5 (C)		171.5 (C)		171.4 (C)
16	6.19 (s)	93.9 (CH)	6.17 (s)	93.9 (CH)	6.17 (s)	93.8 (CH)	5.64 (s)	95.2 (CH)
17	0.79 (d, 4.5)	16.0 (CH_3_)	0.79 (d, 6.0)	16.0 (CH_3_)	0.78 (d, 5.5)	15.9 (CH_3_)	0.75 (d, 5.5)	15.9 (CH_3_)
18	1.58 (s)	19.9 (CH_3_)	1.57 (s)	19.9 (CH_3_)	1.57 (s)	19.9 (CH_3_)	1.57 (s)	19.9 (CH_3_)
19	1.01 (s)	18.0 (CH_3_)	1.01 (s)	17.9 (CH_3_)	1.00 (s)	18.0 (CH_3_)	1.00 (s)	18.0 (CH_3_)
20	0.78 (s)	18.3 (CH_3_)	0.79 (s)	18.2 (CH_3_)	0.79 (s)	18.2 (CH_3_)	0.77 (s)	18.2 (CH_3_)
1’		142.8 (C)		150.2 (C)		145.0 (C)		144.6 (C)
2’		146.9 (C)		145.4 (C)		152.2 (C)		153.6 (C)
3’	6.56 (s)	107.5 (CH)		150.2 (C)	6.58 (s)	110.4 (CH)	6.65 (s)	111.8 (CH)
4’		126.6 (C)		131.0 (C)		127.4 (C)		127.4 (C)
5’	2.63 (m) 2.94 (m)	29.4 (CH_2_)	2.75 (br d, 14.0) 2.80 (m)	24.0 (CH_2_)	2.74 (m) 2.98 (m)	29.4 (CH_2_)	2.80 (m, 2H)	27.3 (CH_2_)
6’	2.72 (m) 2.81 (m)	41.4 (CH_2_)	2.63 (tq, 11.5, 3.5) 2.83 (m)	40.9 (CH_2_)	2.70 (m) 2.80 (m)	41.2 (CH_2_)	2.81 (m, 2H)	42.4 (CH_2_)
7’	4.20 (dd, 10.5, 3.5)	56.3 (CH)	4.06 (br d, 14.0)	56.8 (CH)	4.10 (br d, 14.0)	56.7 (CH)	4.51 (dd, 9.0, 6.5)	59.9 (CH)
8’	2.83 (dd, 9.0, 3.5)	33.7 (CH_2_)	2.73 (br d, 11.0)	33.8 (CH_2_)	2.68 (m)	33.3 (CH_2_)	2.30 (dd, 11.0, 9.0)	46.8 (CH_2_)
	3.33 (dd, 10.5, 9.0)		3.24 (dd, 13.5, 4.0)		3.14 (br d, 11.0)		2.55 (dd, 11.0, 6.5)	
9’		134.0 (C)		134.4 (C)		125.9 (C)	6.90 (d, 10.0)	152.8 (CH)
10’	7.25 (m)	128.4 (CH)	7.24 (d, 7.5)	128.1 (CH)	6.81 (s)	114.3 (CH)	6.44 (d, 10.0)	128.6 (CH)
11’	7.25 (m)	127.8 (CH)	7.22 (dd, 8.0, 7.5)	127.2 (CH)		146.0 (C)		186.0 (C)
12’	7.34 (m)	127.4 (CH)	7.33 (dd, 8.0, 7.5)	127.3 (CH)		145.8 (C)	6.33 (d, 10.0)	127.8 (CH)
13’	8.08 (d, 8.0)	126.9 (CH)	8.24 (d, 7.5)	127.8 (CH)	8.05 (s)	111.2 (CH)	7.04 (d, 10.0)	149.4 (CH)
14’		130.9 (C)		131.7 (C)		123.8 (C)		51.1 (C)
15’		116.6 (C)		122.9 (C)		128.6 (C)		132.8 (C)
16’		126.5 (C)		122.8 (C)		117.1 (C)		134.1 (C)
17’	5.96 (d, 1.4)	100.9 (CH_2_)						
	6.11 (d, 1.4)							
1’-OMe			3.95 (s)	60.9 (CH_3_)	3.66 (s)	60.2 (CH_3_)	3.62 (s)	56.4 (CH_3_)
2’-OMe			3.70 (s)	60.4 (CH_3_)	3.89 (s)	55.8 (CH_3_)	3.82 (s)	61.1 (CH_3_)
3’-OMe			3.92 (s)	60.6 (CH_3_)				
12’-OMe					3.92 (s)	56.0 (CH_3_)		

Data were recorded at 500 MHz (^1^H) and 125 (^13^C) MHz, and coupling constants (J) in Hz were given in parentheses. The assignments were determined by ^1^H, ^13^C, COSY, HMQC, and HMBC NMR spectra.

**Table 2:** Anti-DENV-2 activity of polyalongarins A–D (**1**–**4**).

Compounds	IC_50_ (μM) ^a^	CC_50_ (μM) ^b^	SI ^c^
polyalongarin A (**1**)	4.46 ± 0.12	>200	44.8
polyalongarin B (**2**)	2.80 ± 0.23	50.44 ± 10.20	18
polyalongarin C (**3**)	3.24 ± 0.16	69.11 ± 13.97	21.3
polyalongarin D (**4**)	6.36 ± 0.09	94.47 ± 10.16	14.9
Celastrol ^d^	0.12 ± 0.01		

^a^ The IC_50_ is the concentration of the compound resulting in a 50% inhibition in virus production. ^b^ The CC_50_ is the concentration of the compound causing a 50% growth inhibition of uninfected Huh-7 cells. ^c^ SI: selectivity index. SI = CC_50_/IC_50_. ^d^ Positive control.

## Data Availability

Not applicable.

## Supplementary Materials

The following supporting information can be downloaded at: https://www.mdpi.com/article/10.3390/ph15101218/s1, Figure S1: The ω_16-N,C-16,C-13,C-12_ torsional angles of *cis*-**1** (A), *trans*-1 (B), *cis*-**3m**-16R (C), and *trans*-**3m**-16*R* (D). Figure S2: Proposed biosynthetic pathways of **1**–**4**. Figure S3: Protein sequence alignments of various flavivirus NS2B-NS3 proteases including DENV serotypes **1**-**4**, Zika virus, West Nile virus (WNV), and Murray Valley encephalitis virus (MVEV). Figure S4: Protein crystal structures of NS2B-NS3 proteases in DENV serotypes **1**–**4**. Figure S5: AutoDock 4.2 of binding simulation between DENV2 NS2B-NS3 protease and **1**–**4**. Table S1: Crystal data and structure refinement for **1**. Table S2: Atomic coordinates and equivalent isotropic displacement parameters for **1**. U(eq) is defined as one third of the trace of the orthogonalized U tensor. Table S3: The Predicted Binding Affinity and RMSD bounds of **1**–**4**. Table S4: Python Shell Information of **1**-**4** in Different Models.; Spectra: Mass, IR, UV, CD, and NMR spectra of compounds **1**–**4**. References [35,36,37] are cited in the Supplementary Materials.
