# Supplementary material for: Novel Aporphine- and Proaporphine–Clerodane Hybrids Identified from the Barks of Taiwanese Polyalthia longifolia (Sonn.) Thwaites var. pendula with Strong Anti-DENV2 Activity
Source: Pharmaceuticals (Basel). 2022 Sep 30;15(10):1218. doi: 10.3390/ph15101218 (PMC9610793; doi:10.3390/ph15101218)
Supplement: Supplementary file 1 [file pharmaceuticals-15-01218-s001.zip › pharmaceuticals-1893312-supplementary.pdf]

Supporting Information

Four Novel Aporphine- and Proaporphine-Clerodane Hybrids from the Barks of Taiwanese *Polyalthia longifolia* var. *pendula* with Strong Anti-DENV2 Activity

I-Wen Lo [1], Geng-You Liao [2], Jin-Ching Lee [3], Chi-I Chang [4], Yang-Chang Wu [5,6], Yen-Yu Chen [7], Shang-Pin Liu [8], Huey-Jen Su [9], Chih-I Liu [9], Chia-Yi Kuo [10], Zheng-Yu Lin [10], Tsung-Lin Li [1,*], Yun-Sheng Lin [10,*] and Chia-Ching Liaw [11,12,*]

[1] *Genomics Research Center, Academia Sinica, Taipei 115201, Taiwan.*

[2] *Institute of Physiology, School of Medicine, National Yang Ming Chiao Tung University, Taipei 112304, Taiwan*

[3] *Department of Marine Biotechnology and Resources, National Sun Yat-sen University, Kaohsiung 804201, Taiwan.*

[4] *Department of Biological Science and Technology, National Pingtung University of Science and Technology, Pingtung 912301, Taiwan*

[5] *Chinese Medicine Research and Development Center, China Medical University Hospital, Taichung 404332, Taiwan*

[6] *Graduate Institute of Integrated Medicine, College of Chinese Medicine, China Medical University, Taichung 404333, Taiwan.*

[7] *Department of Education and Research, Taipei City Hospital, Taipei 103212, Taiwan*

[8] *Bachelor of Program in Scientific Agriculture, National Pingtung University of Science and Technology, Pingtung 912301, Taiwan.*

[9] *Department of Nursing, Meiho University, Pingtung 912009, Taiwan.*

[10] *Department of Biological Science and Technology, Meiho University, Pingtung 912009, Taiwan.*

[11] *Division of Chinese Material Medica Development, National Research Institute of Chinese Medicine,*

Taipei 112026, Taiwan

[12] *Department of Biochemical Science and Technology, National Chiayi University, Chiayi 600355, Taiwan*

* Corresponding author: Chia-Ching Liaw, National Research Institute of Chinese Medicine, Taipei 112026, Taiwan. E-mail address: liawcc@nricm.edu.tw (C.C. Liaw). Tsung-Lin Li, Genomics Research Center, Academia Sinica, Taipei 115201, Taiwan. E-mail address: tlli@gate.sinica.edu.tw (T.L. Li). Yun-Sheng Lin, Department of Biological Science and Technology, Meiho University, Pingtung 912009, Taiwan. E-mail address: x00010106@meiho.edu.tw (Y.S. Lin).

Figure S1. The $\omega_{16\text{-N,C-16,C-13,C-12}}$ torsional angles of *cis*-**1** (A), *trans*-**1** (B), *cis*-**3m**-16*R* (C), and *trans*-**3m**-16*R* (D).

Figure S2. Proposed biosynthetic pathways of **1-4**.

Figure S3. Protein sequence alignments of various flavivirus NS2B-NS3 proteases including DENV serotypes **1-4**, Zika virus, West Nile virus (WNV), and Murray Valley encephalitis virus (MVEV).

Figure S4. Protein crystal structures of NS2B-NS3 proteases in DENV serotypes 1-4.

Figure S5. AutoDock 4.2 of binding simulation between DENV2 NS2B-NS3 protease and **1-4**.

Table S1. Crystal data and structure refinement for **1**.

Table S2. Atomic coordinates and equivalent isotropic displacement parameters for **1**. U(eq) is defined as one third of the trace of the orthogonalized U tensor.

Table S3. The Predicted Binding Affinity and RMSD bounds of **1-4**.

Table S4. Python Shell Information of **1-4** in Different Models.

References

Spectra of compounds 1-4

Figure PA-1. ESI-MS spectrum of polyalongarin A (**1**)

Figure PA-2. HR-ESI-MS spectrum of polyalongarin A (**1**)

Figure PA-3. UV spectrum of polyalongarin A (**1**)

Figure PA-4. IR spectrum of polyalongarin A (**1**)

Figure PA-5. CD spectrum of polyalongarin A (**1**)

Figure PA-6. ^{1}H-NMR spectrum of polyalongarin A (**1**) in $CDCl_3$ (500 MHz)

Figure PA-7. ^{13}C-NMR spectrum of polyalongarin A (**1**) in $CDCl_3$ (125 MHz)

Figure PA-8. DEPT-135 and DEPT-90 spectra of polyalongarin A (**1**)

Figure PA-9. HMQC spectrum of polyalongarin A (**1**)

Figure PA-10. COSY spectrum of polyalongarin A (**1**)

Figure PA-11. HMBC spectrum of polyalongarin A (**1**)

Figure PA-12. NOESY spectrum of polyalongarin A (**1**)

Figure PB-1. ESI-MS spectrum of polyalongarin B (**2**)

Figure PB-2. HR-ESI-MS spectrum of polyalongarin B (**2**)

Figure PB-3. UV spectrum of polyalongarin B (**2**)

Figure PB-4. IR spectrum of polyalongarin B (**2**)

Figure PB-5. CD spectrum of polyalongarin B (**2**)

Figure PB-6. ^{1}H-NMR spectrum of polyalongarin B (**2**) in $CDCl_3$ (500 MHz)

Figure PB-7. ^{13}C-NMR spectrum of polyalongarin B (**2**) in $CDCl_3$ (125 MHz)

Figure PB-8. DEPT-135 and DEPT-90 spectra of polyalongarin B (**2**)

Figure PB-9. HMQC spectrum of polyalongarin B (**2**)

Figure PB-10. COSY spectrum of polyalongarin B (**2**)

Figure PB-11. HMBC spectrum of polyalongarin B (**2**)

Figure PB-12. NOESY spectrum of polyalongarin B (**2**)

Figure PC-1. ESI-MS spectrum of polyalongarin C (**3**)

Figure PC-2. HR-ESI-MS spectrum of polyalongarin C (**3**)

Figure PC-3. UV spectrum of polyalongarin C (**3**)

Figure PC-4. IR spectrum of polyalongarin C (**3**)

Figure PC-5. CD spectrum of polyalongarin C (**3**)

Figure PC-6. ^{1}H-NMR spectrum of polyalongarin C (**3**) in $CDCl_3$ (500 MHz)

Figure PC-7. ^{13}C-NMR spectrum of polyalongarin C (**3**) in $CDCl_3$ (125 MHz)

Figure PC-8. DEPT-135 and DEPT-90 spectra of polyalongarin C (**3**)

Figure PC-9. HMQC spectrum of polyalongarin C (**3**)

Figure PC-10. COSY spectrum of polyalongarin C (**3**)

Figure PC-11. HMBC spectrum of polyalongarin C (**3**)

Figure PC-12. NOESY spectrum of polyalongarin C (**3**)

Figure PD-1. ESI-MS spectrum of polyalongarin D (**4**)

Figure PD-2. HR-ESI-MS spectrum of polyalongarin D (**4**)

Figure PD-3. UV spectrum of polyalongarin D (**4**)

Figure PD-4. IR spectrum of polyalongarin D (**4**)

Figure PD-5. CD spectrum of polyalongarin D (**4**)

Figure PD-6. ^{1}H-NMR spectrum of polyalongarin D (**4**) in $CDCl_3$ (500 MHz)

Figure PD-7. ^{13}C-NMR spectrum of polyalongarin D (**4**) in $CDCl_3$ (125 MHz)

Figure PD-8. DEPT-135 and DEPT-90 spectra of polyalongarin D (**4**)

Figure PD-9. HMQC spectrum of polyalongarin D (**4)**

Figure PD-10. COSY spectrum of polyalongarin D (**4**)

Figure PD-11. HMBC spectrum of polyalongarin D (**4**)

Figure PD-12. NOESY spectrum of polyalongarin D (**4**)

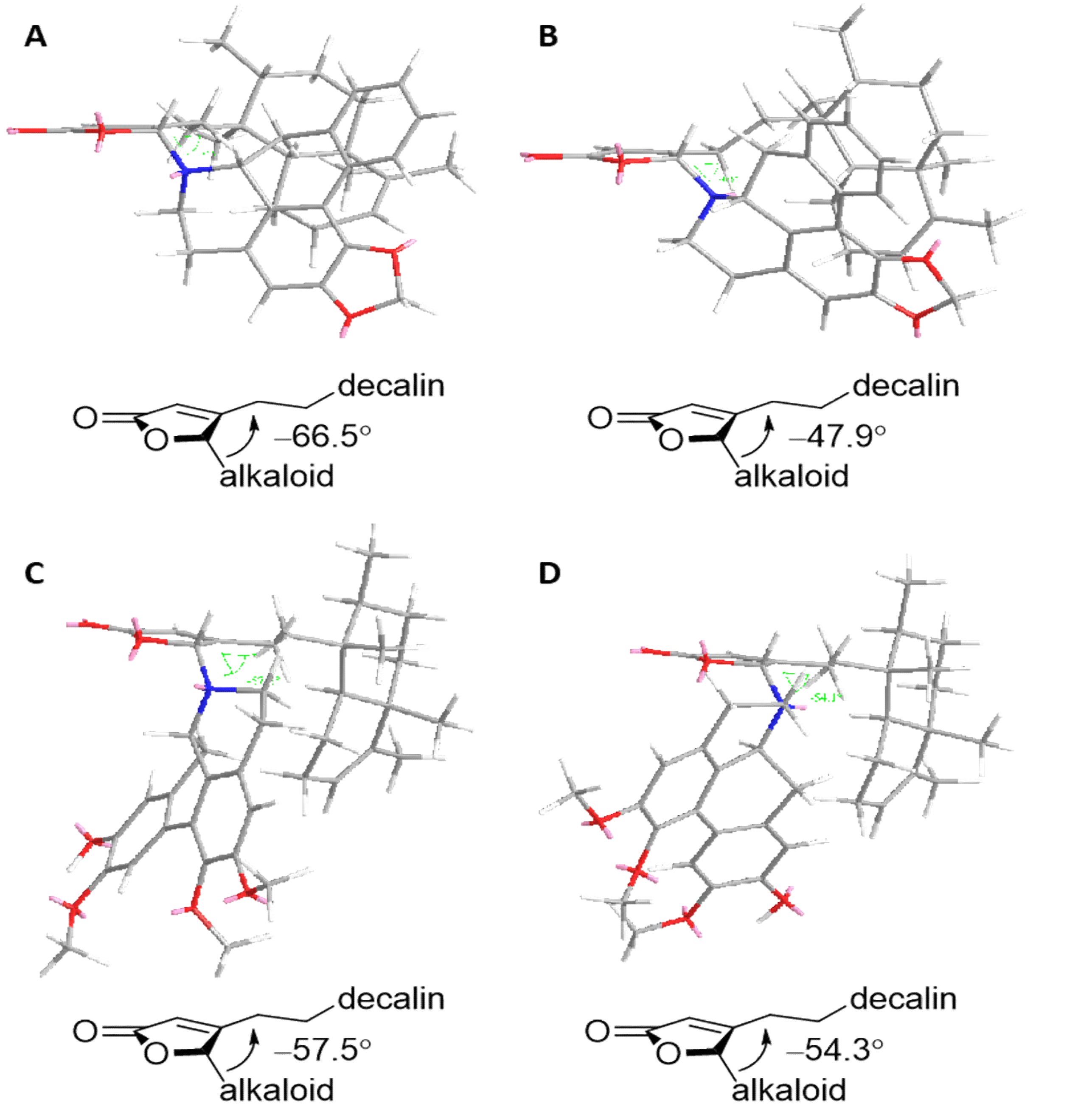

Figure S1. The $\omega_{16\text{-N,C-16,C-13,C-12}}$ torsional angles of *cis*-**1** (A), *trans*-**1** (B), *cis*-**3m**-16*R* (C), and *trans*-**3m**-16*R* (D). The dihedral angles are –66.5°, –47.9°, –57.5°, and –54.3°, respectively calculated after MMFF94 minimization by Chem3D 16.0 with the *β*-oriented H-16. The negative $\omega_{16\text{-N,C-16,C-13,C-12}}$ torsional angles are counterclockwise in both *cis*- and *trans*-form of **3m**-16*R* (*cis*- and *trans*-form here are the configurations of H-7′ and the lone pair on 16-N atom), suggesting that the orientation of the lone pair on N atom does not alter the exciton-coupled system in a manner akin to *cis*-**1** or *trans*-**1**. The dihedral angle between aprophine and ethyl-tetramethyl-octahydronaphthalene (decalin) of **3m**-16*R* remains the same, while the helical conformation of the five-membered γ-lactone ring and aprophine is altered to *P*-configuration by *β*-directed H-7′.

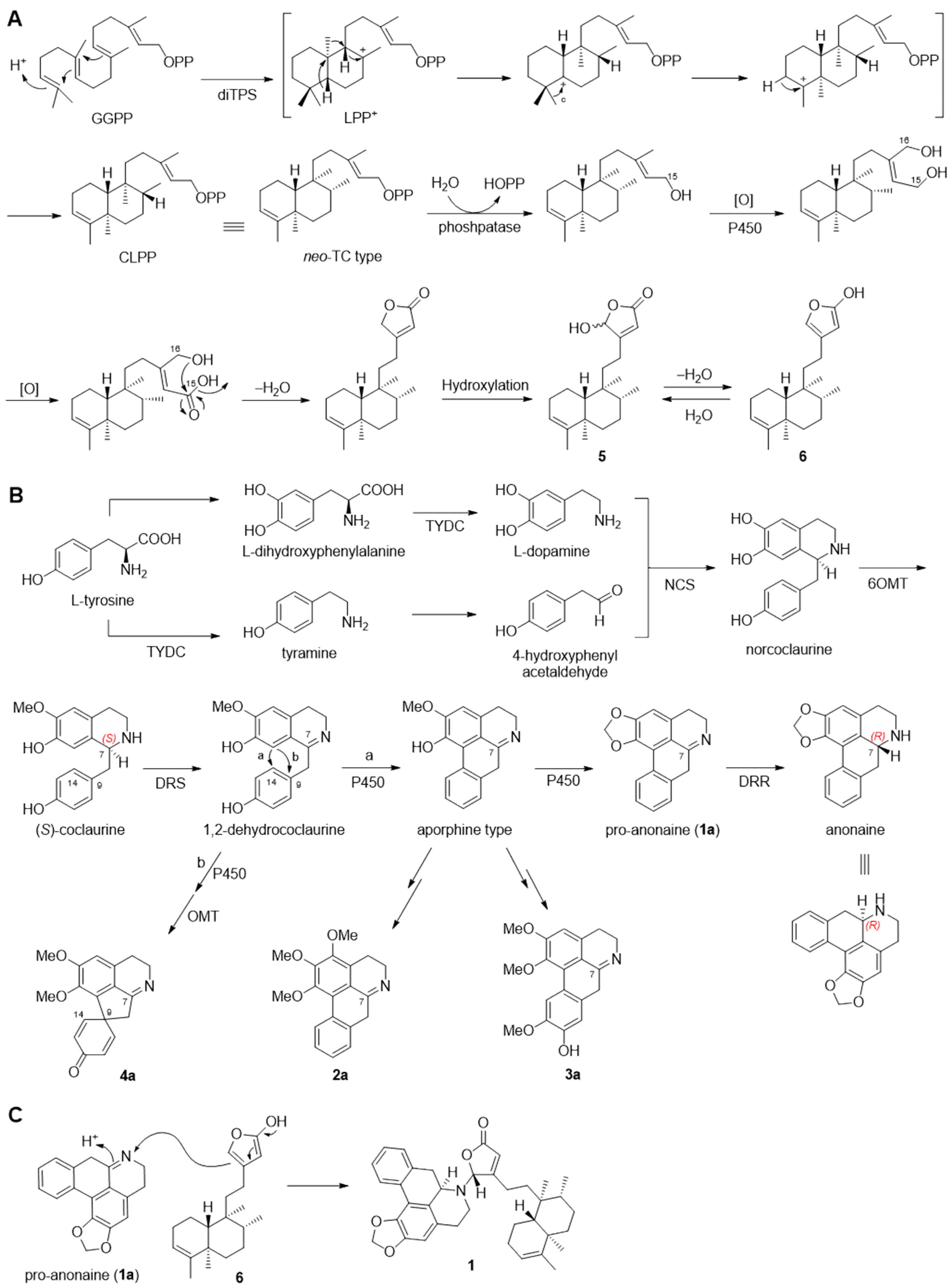

A
GGPP
diTPS
LPP+
CLPP
neo-TC type
H2O
HOPP
phoshpatase
[O]
P450
–H2O
Hydroxylation
5
6
B
L-tyrosine
L-dihydroxyphenylalanine
TYDC
L-dopamine
tyramine
4-hydroxyphenyl acetaldehyde
NCS
norcoclaurine
6OMT
(S)-coclaurine
DRS
1,2-dehydrococlaurine
aporphine type
pro-anonaine (1a)
DRR
anonaine
OMT
4a
2a
3a
C
pro-anonaine (1a)
1

Figure S2. Proposed biosynthetic pathways of **1-4**.[1] (A) Formation of clerodane-type diterpenoids: The common diterpenoid precursor (*E*,*E*,*E*)-geranylgeranyl pyrophosphate (GGPP) is cyclized by a class II diterpene synthetase (diTPS), which protonates the terminal C=C bond of GGPP and cyclizes to the bicyclic (*trans*-decalin) intermediate, labda-13*E*-en-8-yl$^+$ pyrophosphate (LPP$^+$), via sequential anti addition from the internal double bonds of GGPP. Next, a cascade of 1,2-hydride and methyl shifts produce clerodienyl pyrophosphate (CLPP), and then phosphatase removes the diphosphate, leading a fully rearranged clerodane type skeleton backbone. After cytochrome P450 monooxygenase (P450) oxidation, C-16 is hydroxylated and 15,16-dihydroxyl clerodane is formed. Further oxidation at C-15 to form a carboxylic acid. The final clerodane-type diterpenoids, 16-hydroxycleroda-3,13(14)*Z*-dien-15,16-olide (**5**), is given by the following dehydration, hydroxylation, and dehydration. Further dehydration affords the 15-hydroxyfuran-containg clerodane (**6**). (B) Formation of aporphine/proaporphine alkaloids: L-tyrosine is hydroxylated and decarboxylated by DOPA decarboxylase (TYDC) to form L-dopamine and 4-hydroxyphenyl acetaldehyde. These two moieties are condensed to be a benzylisoquinoline-type alkaloid, norcoclaurine, by norcoclaurine synthase (NCS). Norcoclaurine is further methylated by norcoclaurine 6-*O*-methyltransferase (6OMT) to form (*S*)-coclaurine, which is the common precursor of aporphine and proaporphine alkaloids. After the oxidation of (*S*)-coclaurine to 1,2-dehydrococlaurine by 1,2-dehydroreticuline synthase (DRS), following the oxidative coupling aromatization at C-14 (pathway a) produces dehydrogenated aporphines **1a**−**3a** while the dienone-phenol rearrangement at C-9 (pathway b) gives proaporphine **4a**. Then, a series of P450 oxidation, *O*-methylation, and/or hydroxylation are occurred, and these pro-chiral compounds are reduced by 1,2-dehydroreticuline reductase (DRR), leading the production of 7*R* alkaloids, such as anonaine. (C) These clerodane-alkaloid hybrids are assumed to be made from DRR homologues by electron transfer-induced nucleophilic addition reaction, for instance, polyalongarin A (**1)** is a merged by anonaine (**1a**) and **6**.

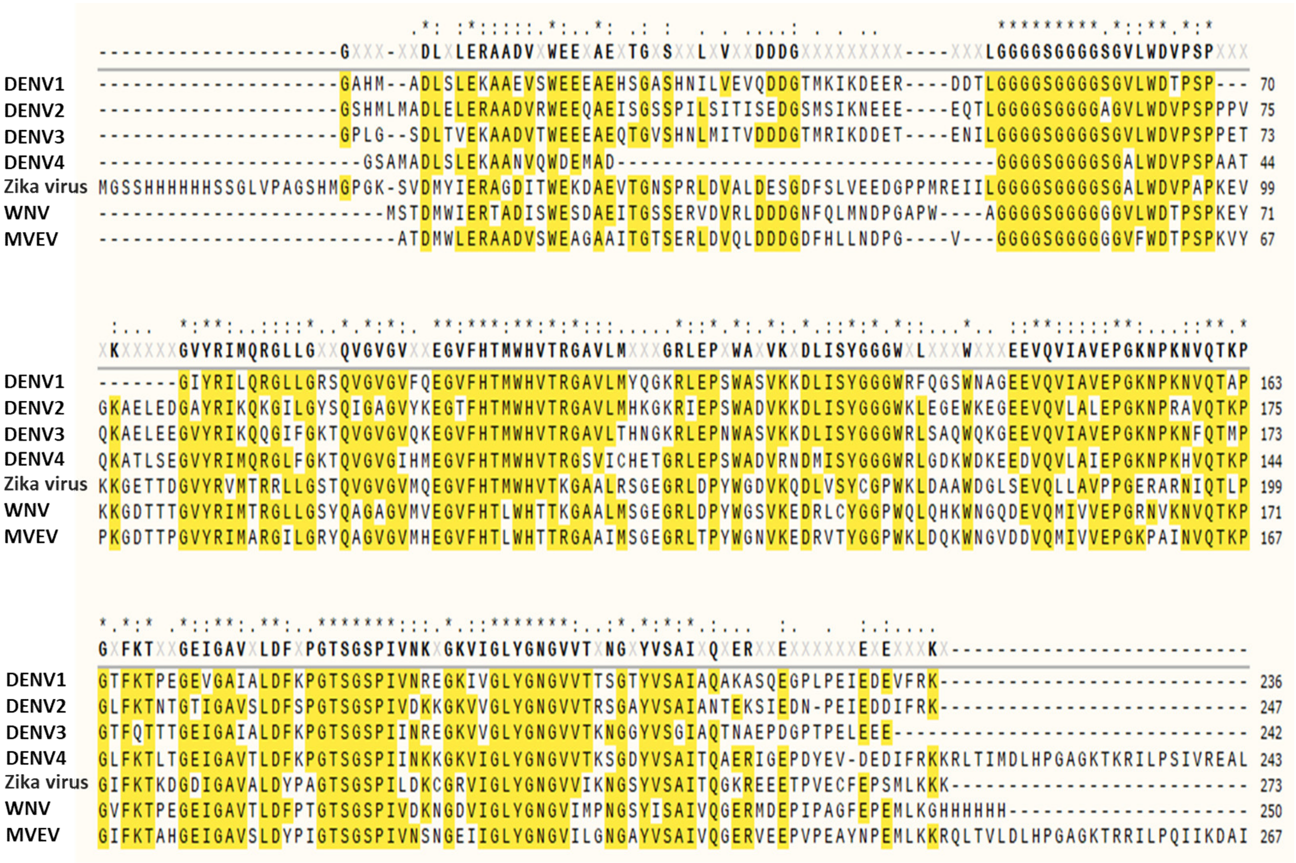

Figure S3. Protein sequence alignments of various flavivirus NS2B-NS3 proteases including DENV serotypes 1-4, Zika virus, West Nile virus (WNV), and Murray Valley encephalitis virus (MVEV). The PDB IDs of these proteases are 3L6P, 6MO1, 3U1I, 2VBC, 5T1V, 2IJO, and 2WV9, respectively. The consensus sequences are highlighted in yellow background. The fully conserved amino acids are marked in asterisk (*), the highly conserved ones are marked in colon (:), and the middle conserved ones are marked in dot (.).

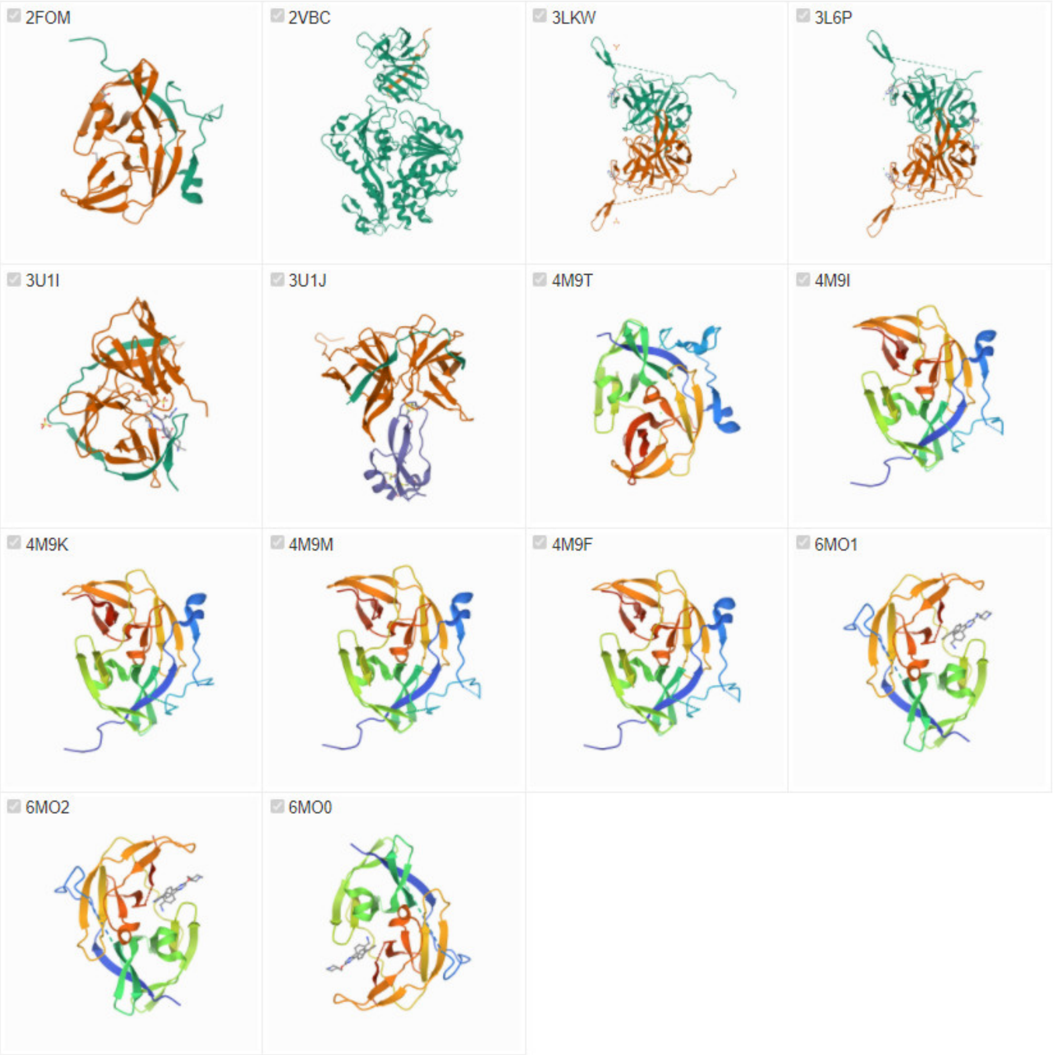

PDB ID	Structure Title
2FOM	Dengue Virus 2 NS2B/NS3 Protease
2VBC	Crystal structure of the NS3 protease-helicase from Dengue virus 4
3LKW	Crystal Structure of Dengue Virus 1 NS2B/NS3 protease active site mutant
3L6P	Crystal Structure of Dengue Virus 1 NS2B/NS3 protease
3U1I	Dengue virus 3 protease covalently bound to a peptide
3U1J	Aprotinin bound to Dengue virus 3 protease
4M9T	NS2B-NS3 protease from dengue virus 2 in the presence of DTNB, a covalent allosteric inhibitor
4M9I	A125C NS2B-NS3 protease from dengue virus 2 at pH 5.5
4M9K	NS2B-NS3 protease from dengue virus 2 at pH 5.5
4M9M	NS2B-NS3 protease from dengue virus 2 at pH 8.5
4M9F	Dengue virus 2 NS2B-NS3 protease A125C variant at pH 8.5
6MO1	Structure of dengue virus 2 protease with an allosteric Inhibitor that blocks replication
6MO2	Structure of dengue virus 2 protease with an allosteric Inhibitor that blocks replication
6MO0	Structure of dengue virus 2 protease with an allosteric Inhibitor that blocks replication

Figure S4. Published protein crystal structures of NS2B-NS3 proteases in DENV serotypes 1–4.

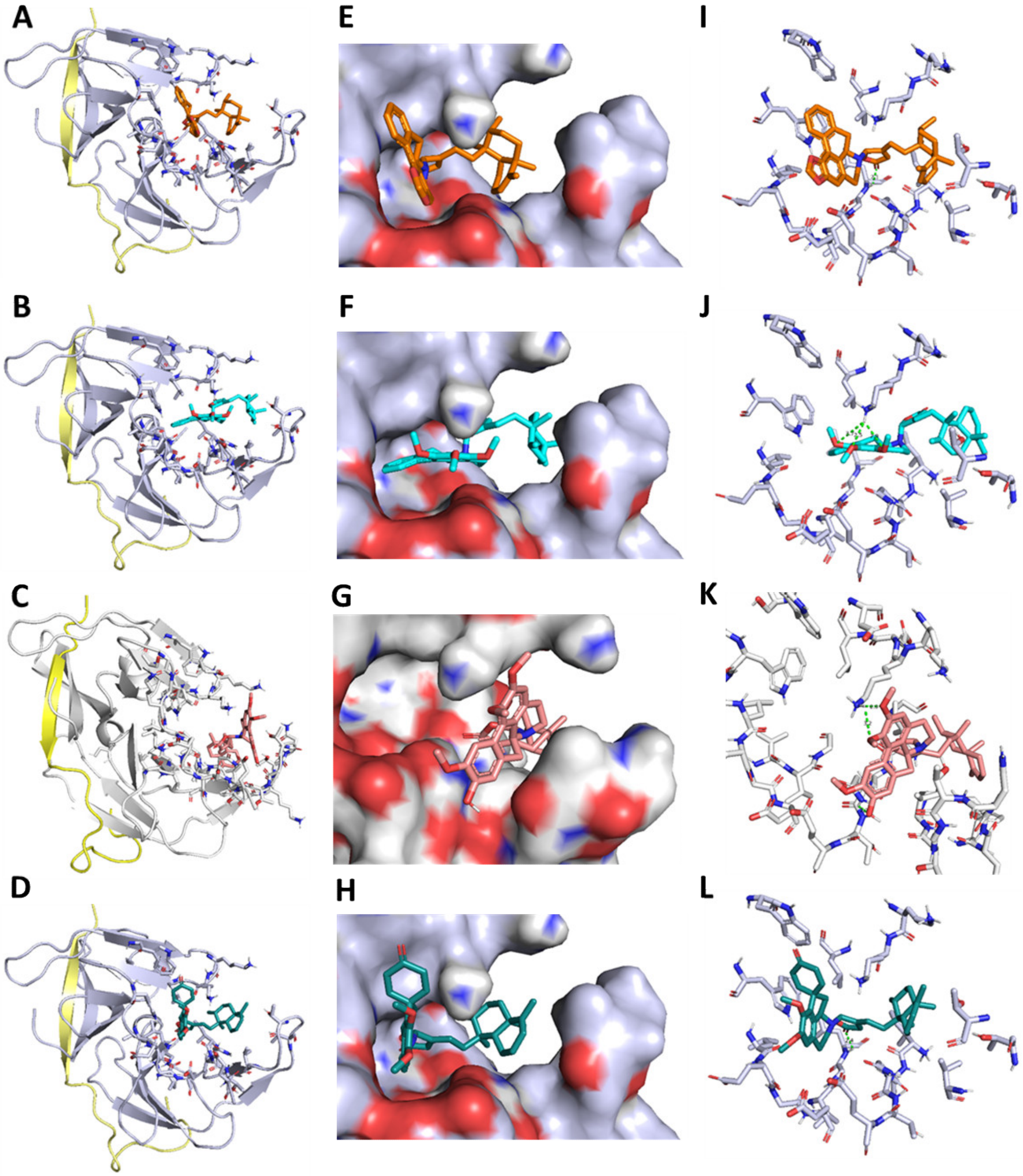

Figure S5. AutoDock 4.2 of binding simulation between DENV2 NS2B-NS3 protease (PDB ID: 6MO1) and compounds **1**–**4**. (A–D) Protein-polyalongarins A–D (**1**–**4**) complexes. Gray, NS3pro; yellow, NS2B; **1**, orange; **2**, cyan; **3**, peach; **4**, green. (E–H) Compounds **1**–**4** are individually located in the pocket of allosteric-site in the hydrophobicity surface model. (I–L) The hydrogen bond(s) between **1**–**4** and NS2B-NS3 protease in the allosteric pocket are shown in green dashed lines. All figures are made in

PyMOL 2.0.[2] Polyalongarins A-D (**1**-**4**) neither stay in the NS3pro active site (H51, D75, and S135) nor interact with NS2B (residues 48–71). Each of them enter into a pocket, in which Lys1074 regulates "opened-active" or "closed-inactive" forms of NS2B-NS3pro.[3] In the DENV NS2B-NS3-polyalongarin A (**1**) complex, a H-bond between the α-NH group of Leu1149 and the O atom of C=O esteric carbonyl group (C-15) of **1** is observed within 2.1 Å. In the complex of DENV NS2B-NS3 protease and polyalongarin B (**2**), the ε-NH_2 group of Lys1074 associates with three O atoms of 1′,2′,3′-trimethoxyl groups of **2** through H-bonds within 3.7, 2.5, and 2.9 Å, respectively. In the DENV NS2B-NS3-polyalongarin C (**3**) complex, three H-bonds are found from the ε-NH_2 group of Lys1074 to the O atom of 1′-OMe (3.2 Å) and 2′-OMe (2.3 Å); and the γ-NH_2 group of Asn1167 to the O atoms of 11′-OH (2.2 Å). In the complex of DENV NS2B-NS3 protease and polyalongarin D (**4**), only one H-bond between the α-NH group of Leu1149 and the O atom of C=O esteric carbonyl group (C-15) of **4** is observed within 2.4 Å.

Table S1. Crystal data and structure refinement for **1**.

Identification code	d18480b	
Empirical formula	C37 H43 N O4	
Formula weight	565.72	
Temperature	200(2) K	
Wavelength	1.54178 Å	
Crystal system	Monoclinic	
Space group	P 21	
Unit cell dimensions	a = 12.2259(2) Å	α= 90°.
	b = 9.50360(10) Å	β= 99.9780(10)°.
	c = 13.0577(2) Å	γ = 90°.
Volume	1494.23(4) $Å^3$	
Z	2	
Density (calculated)	1.257 Mg/m^3	
Absorption coefficient	0.635 mm^{-1}	
F(000)	608	
Crystal size	0.20 x 0.11 x 0.05 mm^3	
Theta range for data collection	3.44 to 66.69°.	
Index ranges	-14<=h<=14, -11<=k<=11, -15<=l<=15	
Reflections collected	17601	
Independent reflections	5242 [R(int) = 0.0485]	
Completeness to theta = 66.69°	99.5 %	
Absorption correction	multi-scan	
Max. and min. transmission	0.9689 and 0.8835	
Refinement method	Full-matrix least-squares on F^2	
Data / restraints / parameters	5242 / 1 / 383	
Goodness-of-fit on F^2	1.044	
Final R indices [I>2sigma(I)]	R1 = 0.0357, wR2 = 0.0905	
R indices (all data)	R1 = 0.0383, wR2 = 0.0942	
Absolute structure parameter	-0.02(17)	
Largest diff. peak and hole	0.147 and -0.211 e.$Å^{-3}$	

Table S2. Atomic coordinates and equivalent isotropic displacement parameters for **1**. U(eq) is defined as one third of the trace of the orthogonalized U tensor.

	x	y	z	U(eq)
C(1)	4920(1)	8975(2)	1248(1)	34(1)
C(2)	6001(1)	8318(2)	1203(1)	32(1)
C(3)	6170(1)	7253(2)	1858(1)	29(1)
C(4)	7113(1)	6237(2)	2089(1)	34(1)
C(5)	8185(1)	6777(2)	1771(2)	33(1)
C(6)	9224(1)	5804(2)	2008(1)	29(1)
C(7)	10124(2)	6517(2)	1494(2)	43(1)
C(8)	9603(1)	5686(2)	3207(2)	38(1)
C(9)	10032(2)	7052(3)	3748(2)	60(1)
C(10)	10484(2)	4535(2)	3495(2)	45(1)
C(11)	10142(2)	3111(2)	3028(2)	42(1)
C(12)	9822(1)	3157(2)	1833(1)	31(1)
C(13)	10877(1)	3372(2)	1339(2)	41(1)
C(14)	9319(2)	1747(2)	1433(2)	39(1)
C(15)	9891(2)	437(2)	1875(2)	58(1)
C(16)	8422(2)	1656(2)	703(2)	47(1)
C(17)	7801(2)	2870(2)	170(2)	50(1)
C(18)	8415(2)	4250(2)	411(1)	38(1)
C(19)	8915(1)	4310(2)	1570(1)	27(1)
C(20)	5231(1)	7192(2)	2473(1)	30(1)
C(21)	4252(2)	5222(2)	1531(1)	34(1)
C(22)	4247(2)	3640(2)	1649(1)	39(1)
C(23)	3799(1)	3189(2)	2604(1)	31(1)
C(24)	3689(1)	4145(2)	3390(1)	28(1)
C(25)	3987(1)	5690(2)	3296(1)	28(1)
C(26)	4505(1)	6269(2)	4356(1)	37(1)
C(27)	3777(1)	6000(2)	5157(1)	32(1)
C(28)	3729(2)	6977(2)	5938(1)	38(1)
C(29)	3100(2)	6714(2)	6710(1)	42(1)
C(30)	2526(2)	5465(2)	6697(1)	41(1)
C(31)	2564(2)	4482(2)	5922(1)	37(1)
C(32)	3194(1)	4736(2)	5137(1)	30(1)
C(33)	3272(1)	3719(2)	4297(1)	29(1)
C(34)	2968(1)	2324(2)	4323(1)	32(1)
C(35)	2249(2)	248(2)	4682(2)	47(1)
C(36)	3095(2)	1373(2)	3549(1)	35(1)
C(37)	3512(2)	1762(2)	2682(1)	37(1)
N(1)	4744(1)	5848(2)	2533(1)	30(1)
O(1)	4466(1)	9952(2)	761(1)	48(1)
O(2)	4451(1)	8278(1)	1980(1)	36(1)
O(3)	2566(1)	1602(1)	5112(1)	42(1)
O(4)	2784(1)	36(2)	3812(1)	51(1)

Table S3. The Predicted Binding Affinity and RMSD bounds of **1−4**.

Ligand & Energy Minimization	Binding Affinity (kcal/mol)	Mode	RMSD lower bound	RMSD upper bound
cpd_1_uff_E=949.78	-10.2	0	0	0
cpd_1_uff_E=949.78	-10.2	1	0.954	2.48
cpd_1_uff_E=949.78	-10	2	1.116	2.038
cpd_1_uff_E=949.78	-10	3	3.771	9.962
cpd_1_uff_E=949.78	-9.8	4	2.28	8.337
cpd_1_uff_E=949.78	-9.7	5	2.789	4.971
cpd_1_uff_E=949.78	-9.6	6	6.388	11.439
cpd_1_uff_E=949.78	-9.5	7	2.516	4.412
cpd_1_uff_E=949.78	-9.4	8	4.716	8.426
cpd_2_uff_E=1094.59	-9.2	0	0	0
cpd_2_uff_E=1094.59	-8.7	1	1.209	2.171
cpd_2_uff_E=1094.59	-8.3	2	1.708	3.228
cpd_2_uff_E=1094.59	-7.9	3	2.441	5.109
cpd_2_uff_E=1094.59	-7.9	4	1.48	3.303
cpd_2_uff_E=1094.59	-7.8	5	1.2	1.974
cpd_2_uff_E=1094.59	-7.6	6	2.591	5.318
cpd_2_uff_E=1094.59	-7.2	7	3.701	9.774
cpd_2_uff_E=1094.59	-7.2	8	4.396	9.393
cpd_3_uff_E=961.60	-8.9	0	0	0
cpd_3_uff_E=961.60	-8.7	1	6.587	11.565
cpd_3_uff_E=961.60	-8.1	2	6.22	10.061
cpd_3_uff_E=961.60	-8.1	3	5.914	10.884
cpd_3_uff_E=961.60	-8.0	4	8.32	12.125
cpd_3_uff_E=961.60	-8.0	5	7.674	11.566
cpd_3_uff_E=961.60	-7.9	6	6.616	10.599
cpd_3_uff_E=961.60	-7.9	7	0.964	2.433
cpd_3_uff_E=961.60	-7.7	8	3.134	9.493
cpd_4_uff_E=945.54	-9.8	0	0	0
cpd_4_uff_E=945.54	-9.4	1	2.831	9.192
cpd_4_uff_E=945.54	-9.3	2	5.743	10.916
cpd_4_uff_E=945.54	-9.0	3	8.264	11.236
cpd_4_uff_E=945.54	-8.9	4	4.42	7.09
cpd_4_uff_E=945.54	-8.9	5	3.112	9.452
cpd_4_uff_E=945.54	-8.6	6	6.809	12.177
cpd_4_uff_E=945.54	-8.6	7	5.625	11.213
cpd_4_uff_E=945.54	-8.6	8	2.549	9.904

Table S4. Python Shell Information of **1–4** in Different Models.

Python 2.6.5 (r265:79096, Mar 19 2010, 21:48:26) [MSC v.1500 32 bit (Intel)] on win32

Type "help", "copyright", "credits" or "license" for more information.

adding gasteiger charges to peptide

cpd_1_uff_E=949.78_out_model1 : -10.2 , 0.0 , 0.0

cpd_1_uff_E=949.78_out_model2 : -10.2 , 0.954 , 2.48

cpd_1_uff_E=949.78_out_model3 : -10.0 , 1.116 , 2.038

cpd_1_uff_E=949.78_out_model4 : -10.0 , 3.771 , 9.962

cpd_1_uff_E=949.78_out_model5 : -9.8 , 2.28 , 8.337

cpd_1_uff_E=949.78_out_model6 : -9.7 , 2.789 , 4.971

cpd_1_uff_E=949.78_out_model7 : -9.6 , 6.388 , 11.439

cpd_1_uff_E=949.78_out_model8 : -9.5 , 2.516 , 4.412

cpd_1_uff_E=949.78_out_model9 : -9.4 , 4.716 , 8.426

cpd_2_uff_E=1094.59_out_model1 : -9.2 , 0.0 , 0.0

cpd_2_uff_E=1094.59_out_model2 : -8.7 , 1.209 , 2.171

cpd_2_uff_E=1094.59_out_model3 : -8.3 , 1.708 , 3.228

cpd_2_uff_E=1094.59_out_model4 : -7.9 , 2.441 , 5.109

cpd_2_uff_E=1094.59_out_model5 : -7.9 , 1.48 , 3.303

cpd_2_uff_E=1094.59_out_model6 : -7.8 , 1.2 , 1.974

cpd_2_uff_E=1094.59_out_model7 : -7.6 , 2.591 , 5.318

cpd_2_uff_E=1094.59_out_model8 : -7.2 , 3.701 , 9.774

cpd_2_uff_E=1094.59_out_model9 : -7.2 , 4.396 , 9.393

cpd_3_uff_E=961.60_out_model1 : -8.9 , 0.0 , 0.0

cpd_3_uff_E=961.60_out_model2 : -8.7 , 6.615 , 11.486

cpd_3_uff_E=961.60_out_model3 : -8.0 , 8.306 , 12.001

cpd_3_uff_E=961.60_out_model4 : -8.0 , 0.954 , 2.474

cpd_3_uff_E=961.60_out_model5 : -8.0 , 5.978 , 9.748

cpd_3_uff_E=961.60_out_model6 : -7.9 , 9.125 , 13.527

cpd_3_uff_E=961.60_out_model7 : -7.8 , 11.507 , 16.863

cpd_3_uff_E=961.60_out_model8 : -7.8 , 8.653 , 10.883

cpd_3_uff_E=961.60_out_model9 : -7.8 , 2.032 , 4.046

cpd_4_uff_E=945.54_out_model1 : -9.8 , 0.0 , 0.0

cpd_4_uff_E=945.54_out_model2 : -9.4 , 2.831 , 9.192

cpd_4_uff_E=945.54_out_model3 : -9.3 , 5.743 , 10.916

cpd_4_uff_E=945.54_out_model4 : -9.0 , 8.264 , 11.236

cpd_4_uff_E=945.54_out_model5 : -8.9 , 4.42 , 7.09

cpd_4_uff_E=945.54_out_model6 : -8.9 , 3.112 , 9.452

cpd_4_uff_E=945.54_out_model7 : -8.6 , 6.809 , 12.177

cpd_4_uff_E=945.54_out_model8 : -8.6 , 5.625 , 11.213

cpd_4_uff_E=945.54_out_model9 : -8.6 , 2.549 , 9.904

References

(1) (a) Misra, P.; Sashidhara, K. V.; Singh, S. P.; Kumar, A.; Gupta, R.; Chaudhaery, S. S.; Gupta, S. S.; Majumder, H. K.; Saxena, A. K.; Dube, A. 16α-Hydroxycleroda-3,13(14)Z-dien-15,16-olide from *Polyalthia longifolia*: a safe and orally active antileishmanial agent inflammation. *Br. J. Pharmacol.* **2010**, *159*, 1143–1150. (b) Potter, K. C.; Zi, J. C.; Hong, Y. J.; Schulte, S.; Malchow, B.; Tantillo, D. J.; Peters, R. J. Blocking Deprotonation with Retention of Aromaticity in a Plant *ent*-Copalyl Diphosphate Synthase Leads to Product Rearrangement. *Angew. Chem. Int. Ed.* **2016**, *55*, 634–638. (c) Yang, M.; Zhu, L. P.; Li, L.; Li, J. J.; Xu, L. M.; Feng, J.; Liu, Y. L. Digital Gene Expression Analysis Provides Insight into the Transcript Profile of the Genes Involved in Aporphine Alkaloid Biosynthesis in Lotus (*Nelumbo nucifera*). *Front. Plant Sci.* **2017**, *8*, 80. (d) Hirata K.; Poeaknapo C.; Schmidt J. Zenk M.H. 1,2-Dehydroreticuline synthase, the branch point enzyme opening the morphinan biosynthetic pathway. *Phytochemistry* **2004**, *65*, 1039–1046. (e) De-Eknamkul W. and Zenk M.H. Purification and properties of 1,2-dehydroreticuline reductase from *Papaver somniferum* seedlings. *Phytochemistry* **1992**, *31*, 813–821.

(2) The PyMOL Molecular Graphics System, Version 2.0 Schrödinger, LLC. (https://pymol.org/2/)

(3) (a) Erbel, P.; Schiering, N.; D'Arcy, A.; Renatus, M.; Kroemer, M.; Lim, S. P.; Yin, Z.; Keller, T. H.; Vasudevan, S. G.; Hommel, U. Structural basis for the activation of flaviviral NS3 proteases from dengue and West Nile virus. *Nat. Struct. Mol. Biol.* **2006**, *13*, 372−373. (b) Yildiz, M.; Ghosh, S.; Bell, J. A.; Sherman, W.; Hardy, J. A. Allosteric Inhibition of the NS2B-NS3 Protease from Dengue Virus. *ACS Chem. Biol.* **2013**, *8*, 2744−2752. (c) Yao, Y.; Huo, T.; Lin, Y. L.; Nie, S. Y.; Wu, F. R.; Hua, Y. D.; Wu, J. Y.; Kneubehl, A. R.; Vogt, M. B.; Rico-Hesse, R.; Song, Y. C. Discovery, X-ray Crystallography and Antiviral Activity of Allosteric Inhibitors of Flavivirus NS2B-NS3 Protease. *J. Am. Chem. Soc.* **2019**, *141*, 6832−6836.

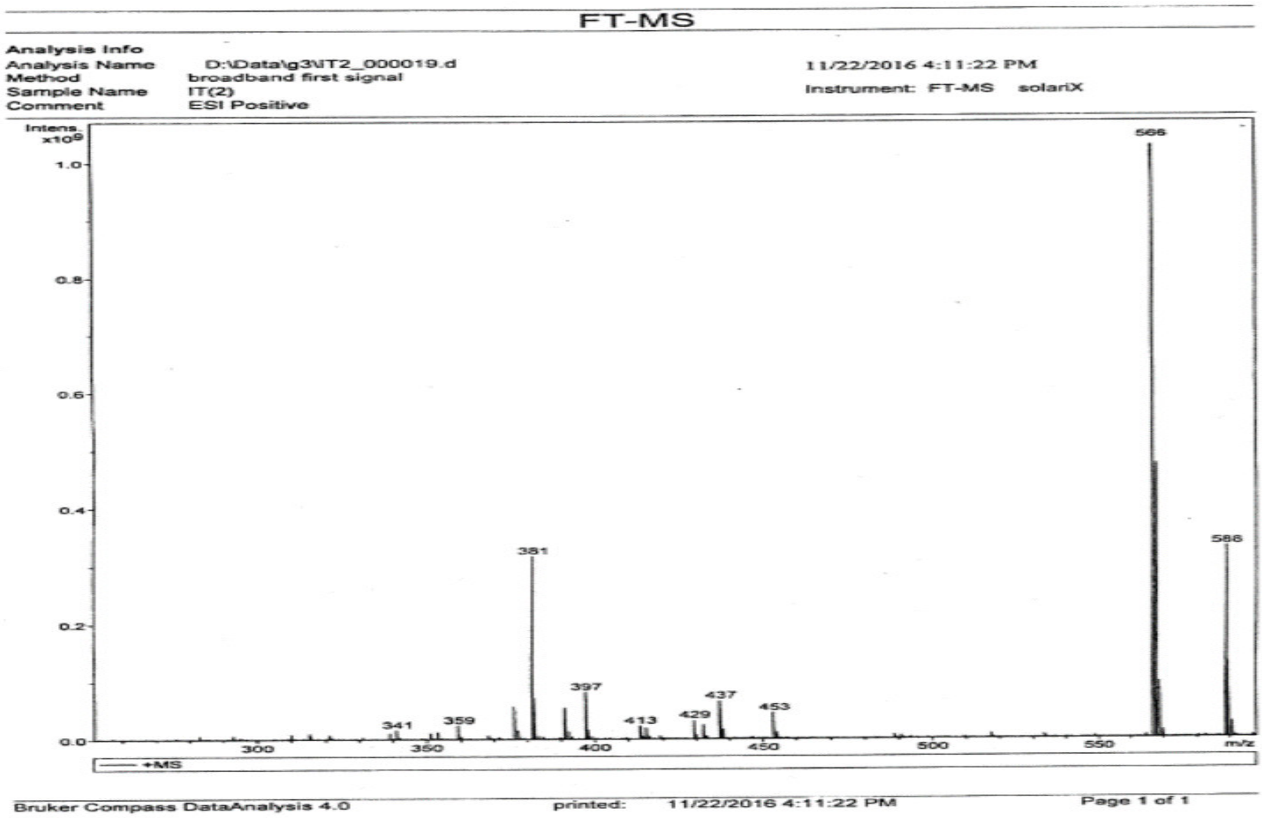

Figure PA-1. ESI-MS spectrum of polyalongarin A (**1**)

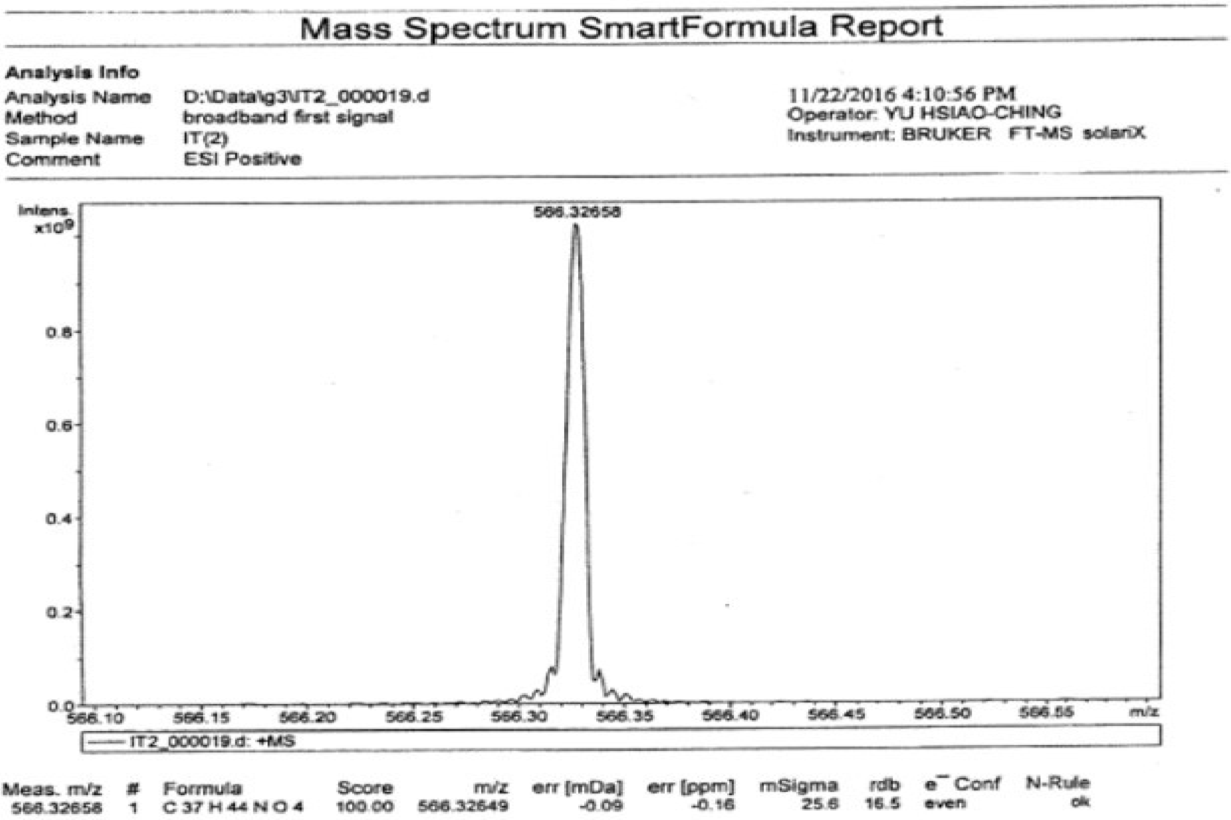

Figure PA-2. HR-ESI-MS spectrum of polyalongarin A (**1**)

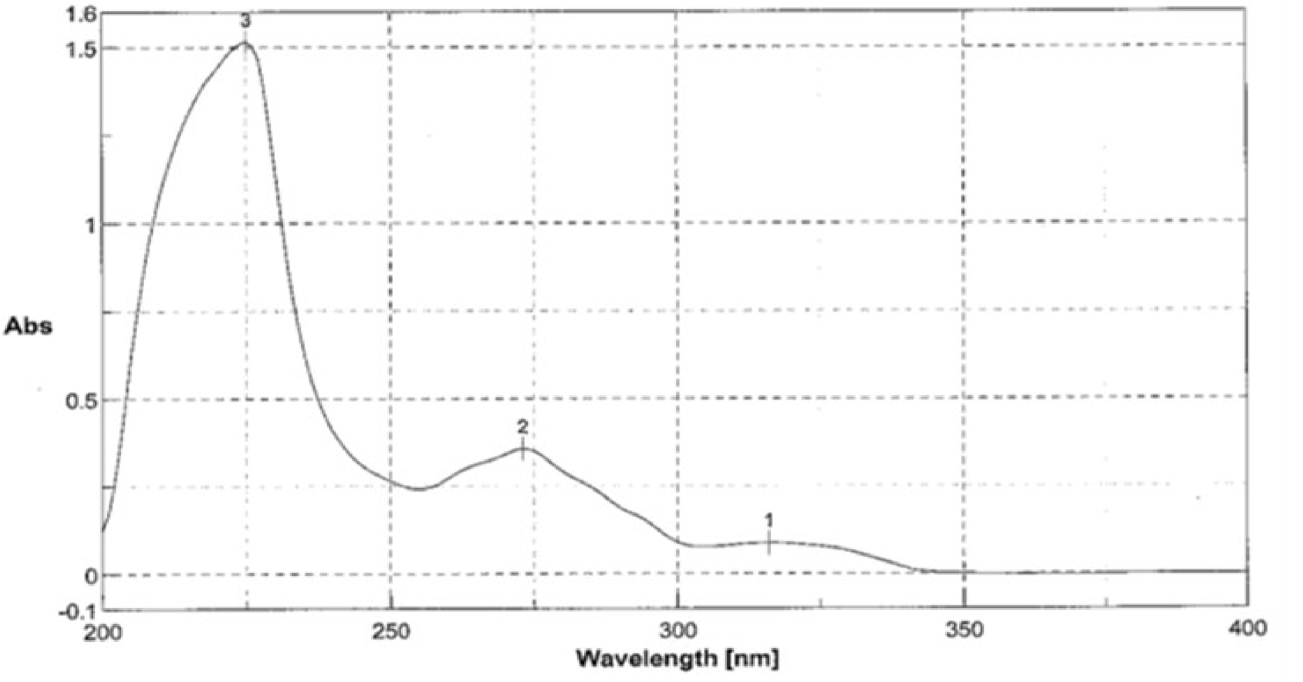

No.	nm	Abs	No.	nm	Abs	No.	nm	Abs
1	316	0.08756	2	273	0.35633	3	225	1.51558

Figure PA-3. UV spectrum of polyalongirin A (**1**)

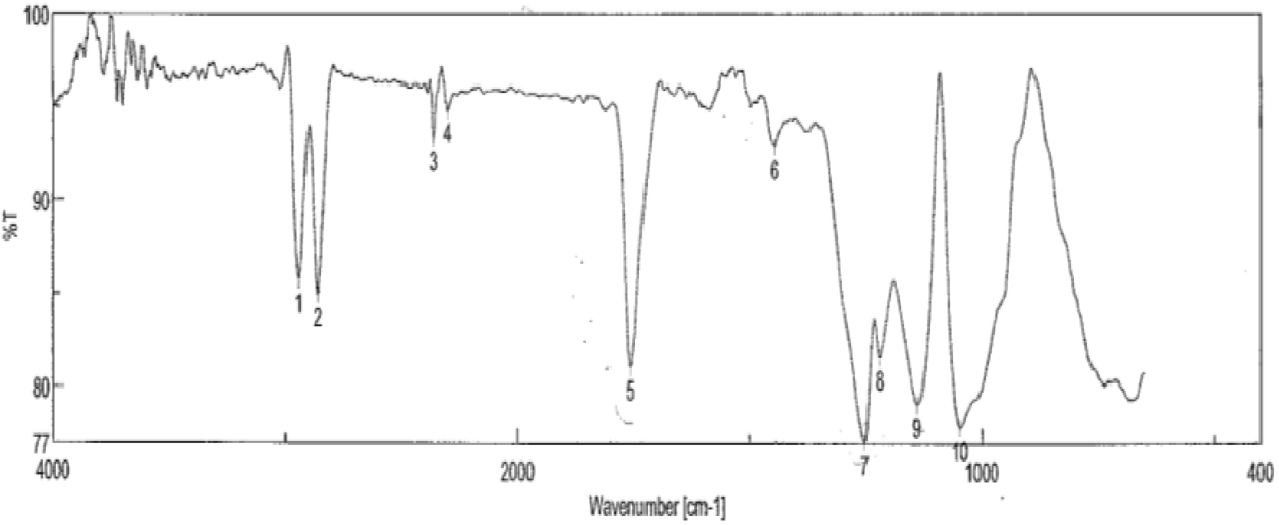

[Result of Peak Picking]

No.	Position	Intensity	No.	Position	Intensity	No.	Position	Intensity
1	2939.95	85.7378	2	2860.88	84.9666	3	2361.41	93.251
4	2300.66	94.8141	5	1755.87	81.0985	6	1447.31	92.8378
7	1252.54	77.0856	8	1220.72	81.6289	9	1139.72	79.1067
10	1047.16	77.8573						

Figure PA-4. IR spectrum of polyalongarin A (**1**)

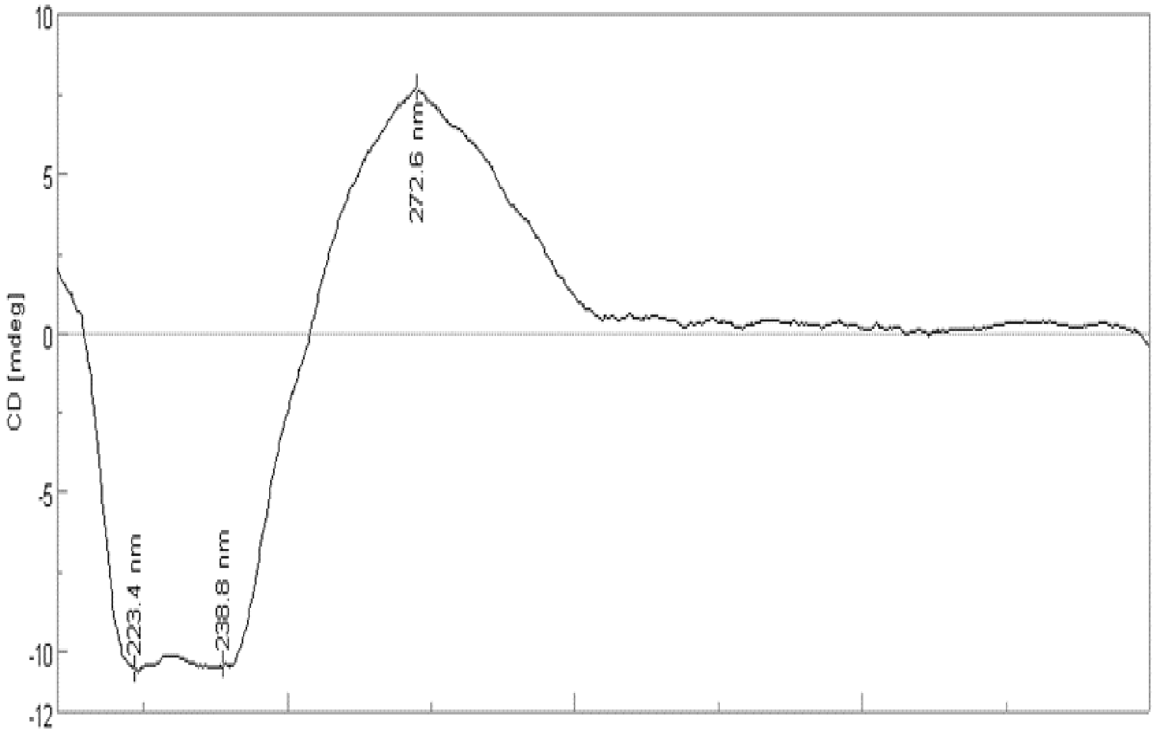

Figure PA-5. CD spectrum of polyalongarin A (**1**)

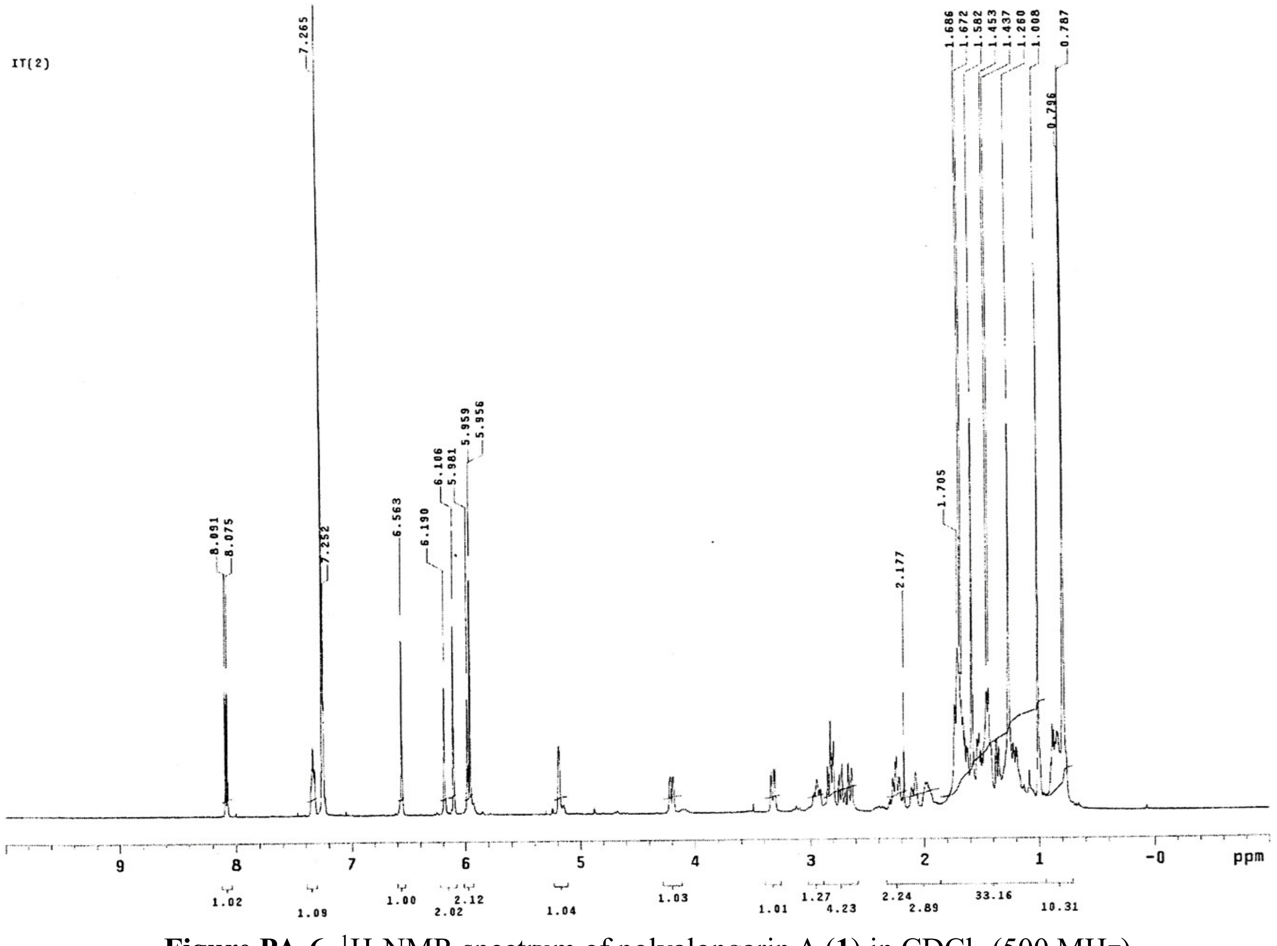

Figure PA-6. ^{1}H-NMR spectrum of polyalongarin A (**1**) in $CDCl_3$ (500 MHz)

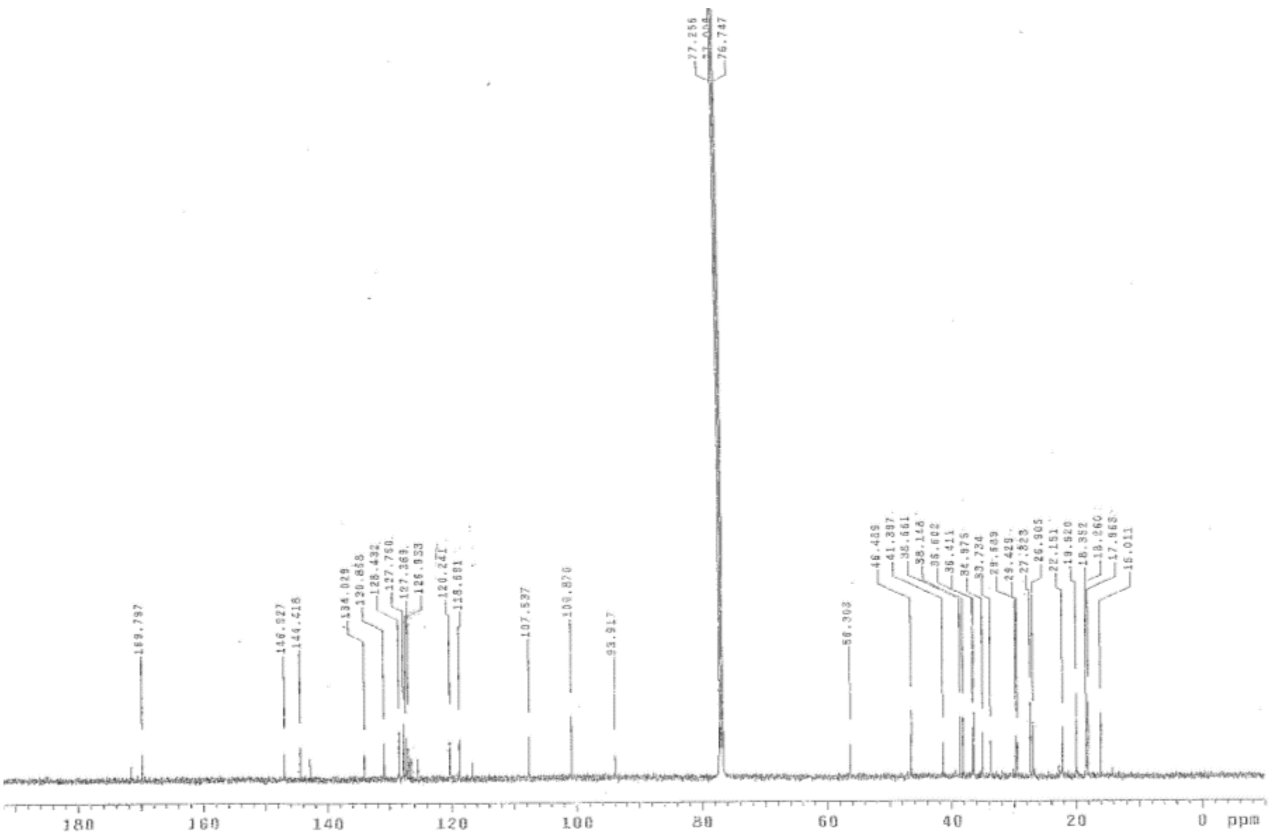

Figure PA-7. ^{13}C-NMR spectrum of polyalongarin A (**1**) in $CDCl_3$ (125 MHz)

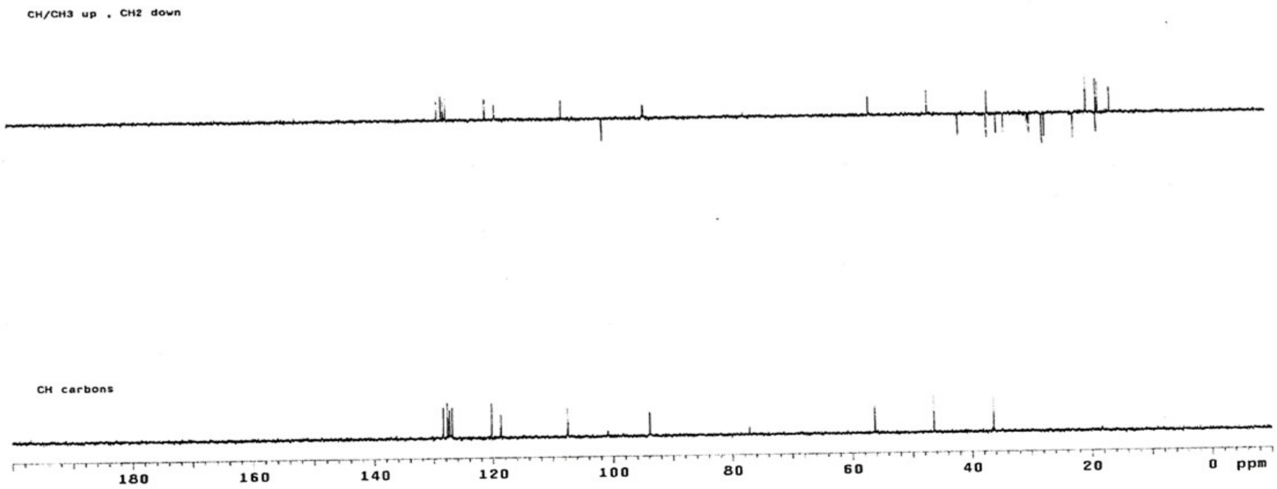

Figure PA-8. DEPT-135 and DEPT-90 spectra of polyalongarin A (**1**)

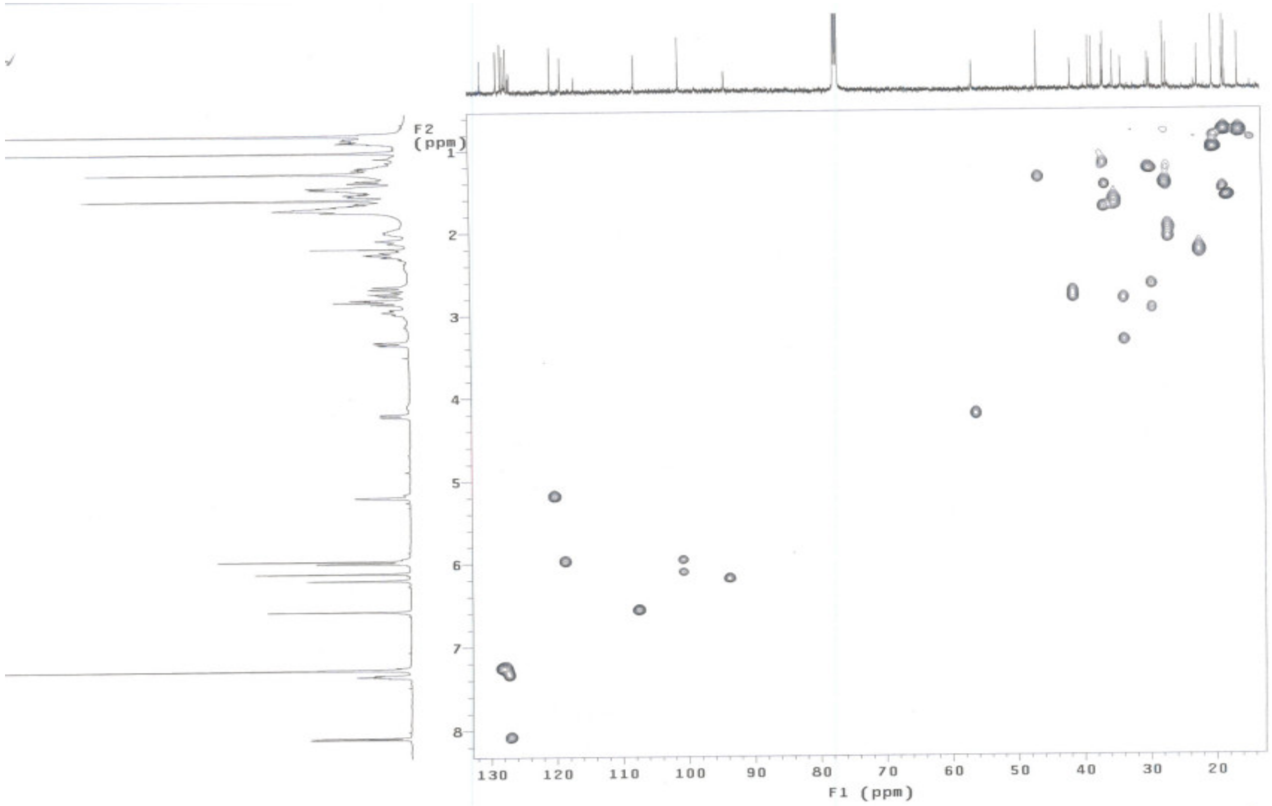

Figure PA-9. HMQC spectrum of polyalongarin A (**1**)

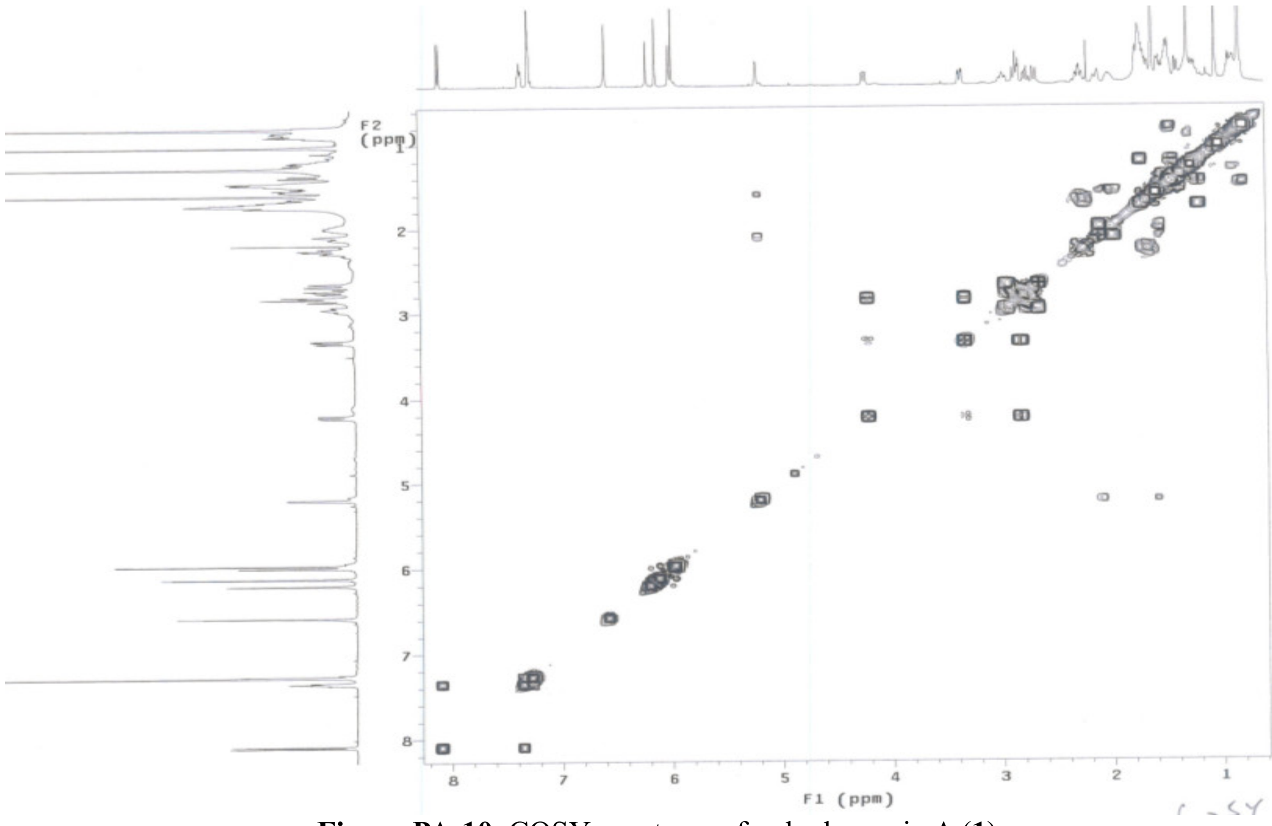

Figure PA-10. COSY spectrum of polyalongarin A (**1**)

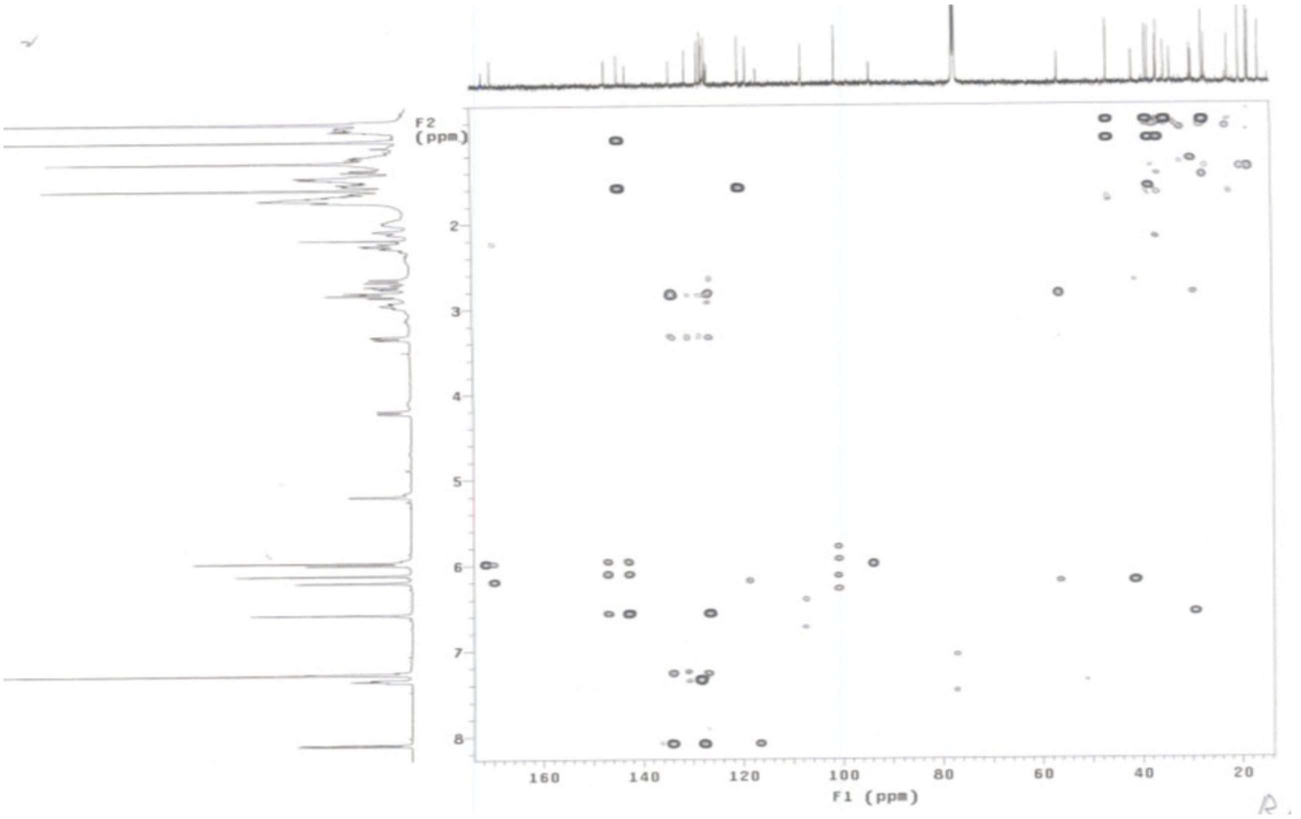

Figure PA-11. HMBC spectrum of polyalongarin A (**1**)

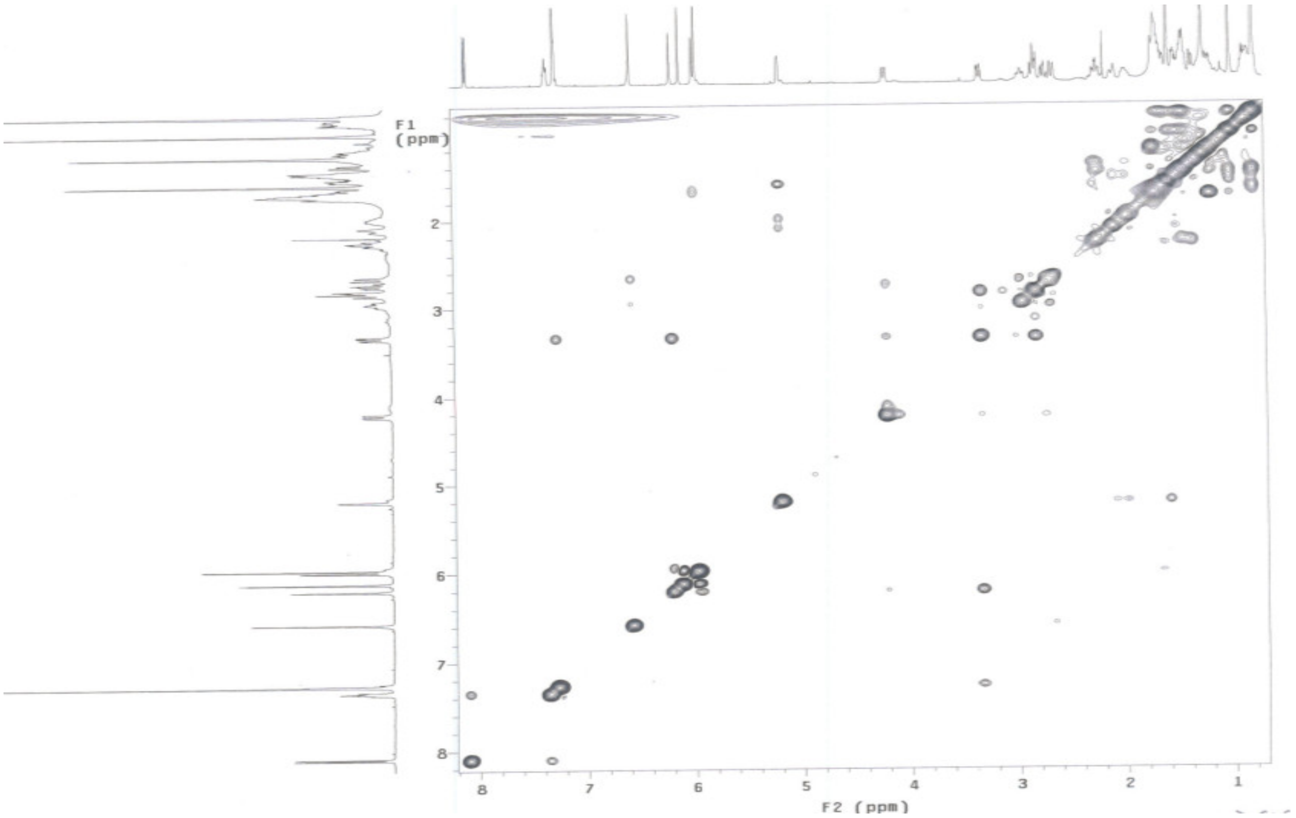

Figure PA-12. NOESY spectrum of polyalongarin A (**1**)

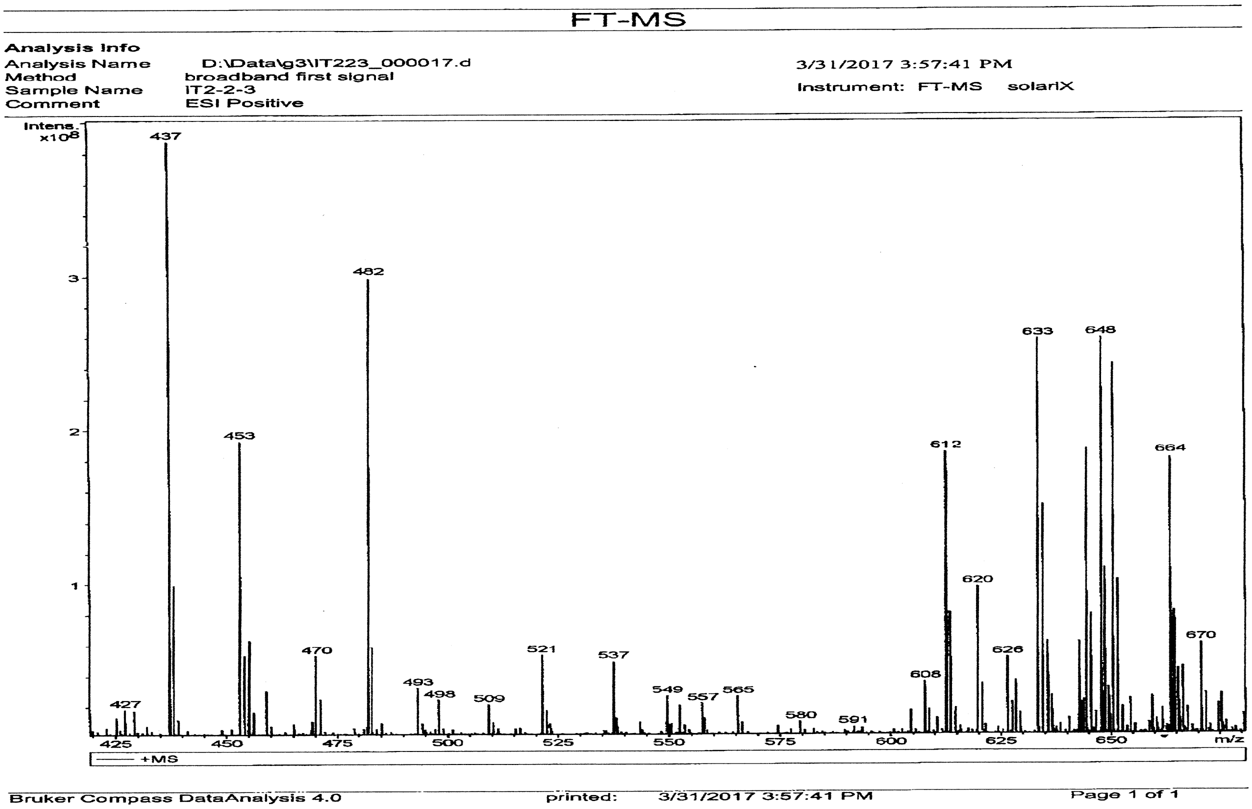

Figure PB-1. ESI-MS spectrum of polyalongarin B (**2**)

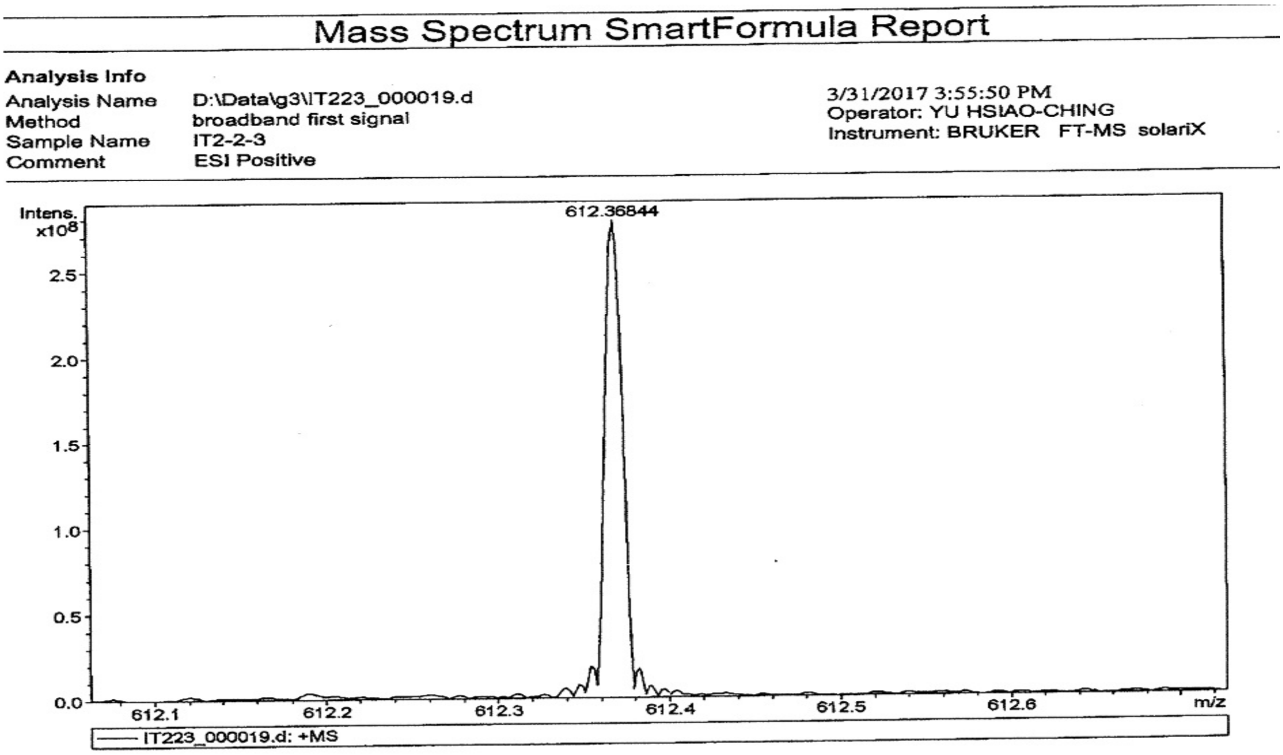

Meas. m/z	#	Formula	Score	m/z	err [mDa]	err [ppm]	mSigma	rdb	e⁻ Conf	N-Rule
612.36844	1	C 39 H 50 N O 5	100.00	612.36835	-0.09	-0.15	15.2	15.5	even	ok

Figure PB-2. HR-ESI-MS spectrum of polyalongarin B (**2**)

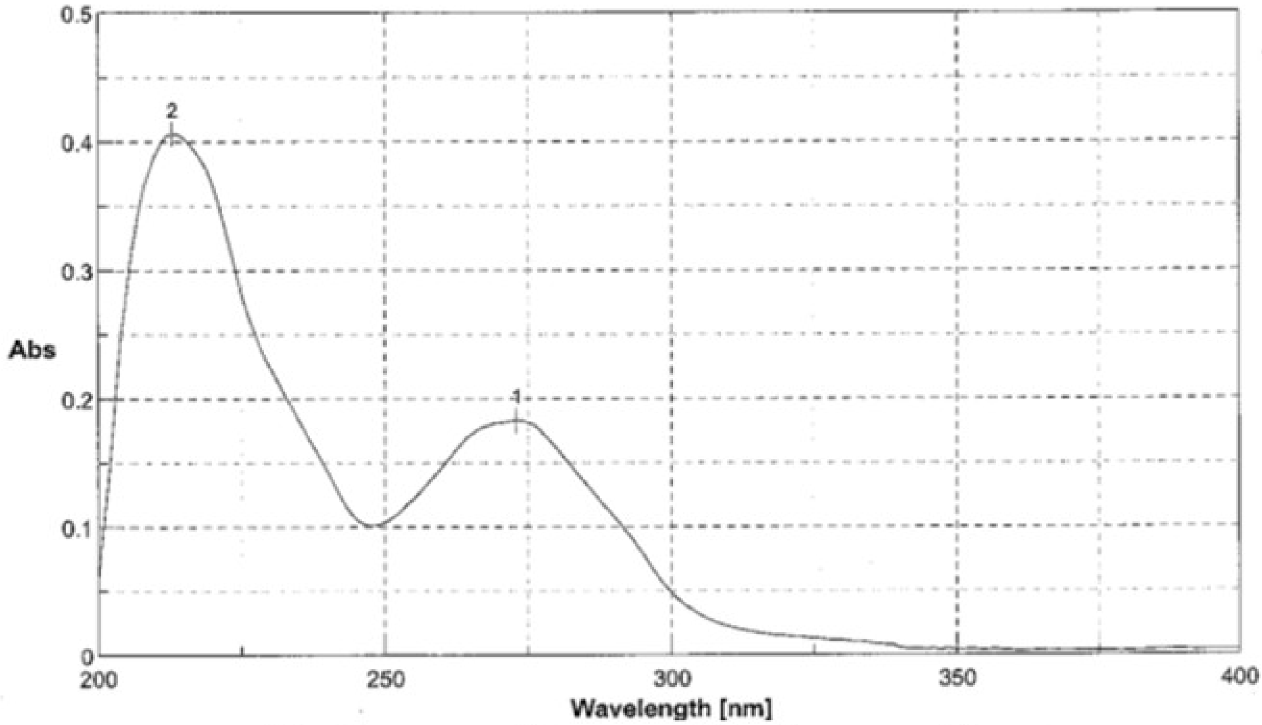

No.	nm	Abs	No.	nm	Abs
1	273	0.1832	2	213	0.40598

Figure PB-3. UV spectrum of polyalongarin B (**2**)

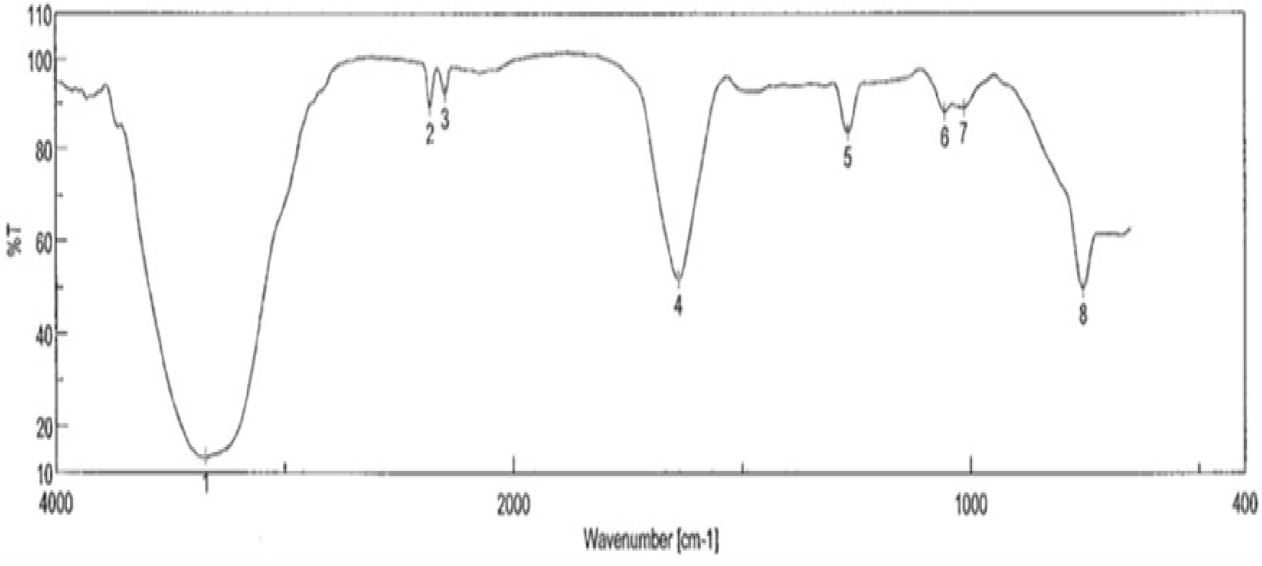

[Result of Peak Picking]

No.	Position	Intensity	No.	Position	Intensity	No.	Position	Intensity
1	3346.85	13.2722	2	2362.37	89.056	3	2296.8	92.2294
4	1639.2	51.9965	5	1267.97	83.9934	6	1055.84	88.3503
7	1014.37	89.2479	8	753.066	49.9424			

Figure PB-4. IR spectrum of polyalongarin B (**2**)

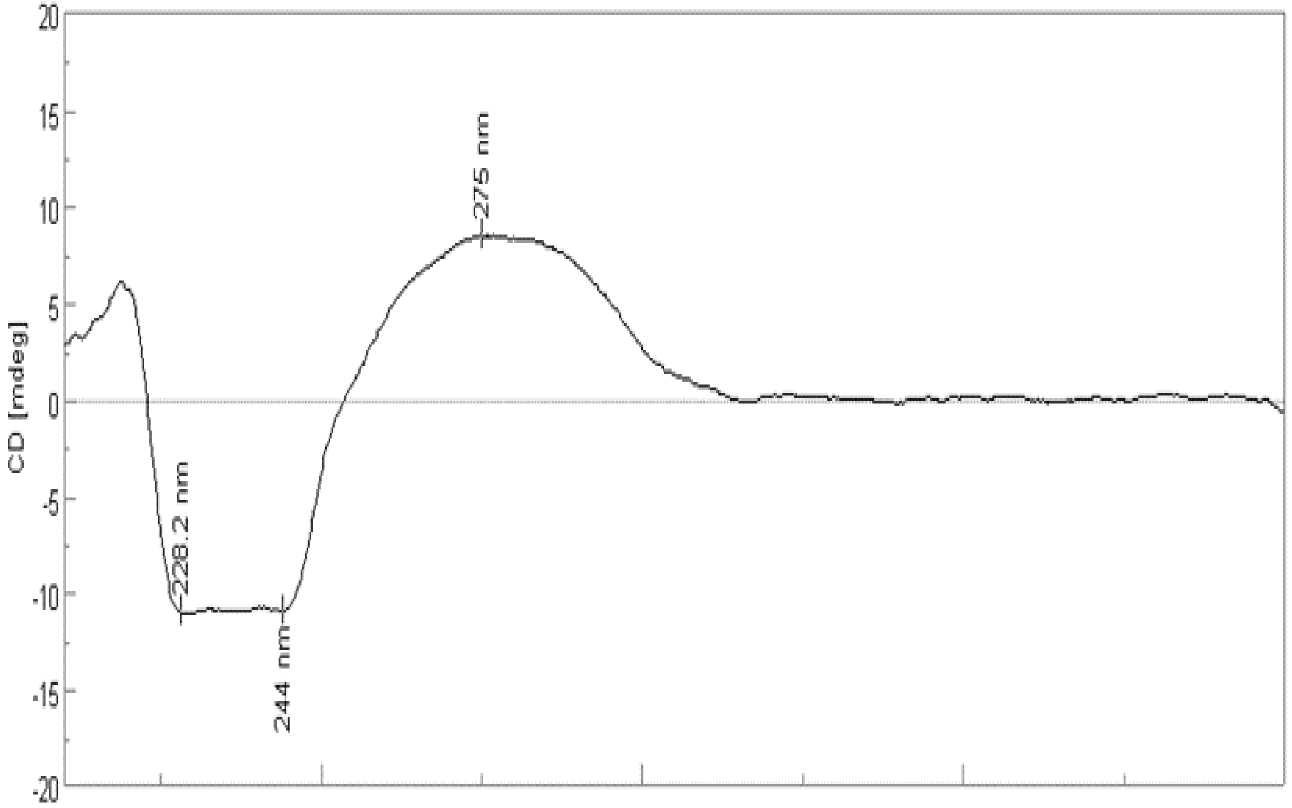

Figure PB-5. CD spectrum of polyalongarin B (**2**)

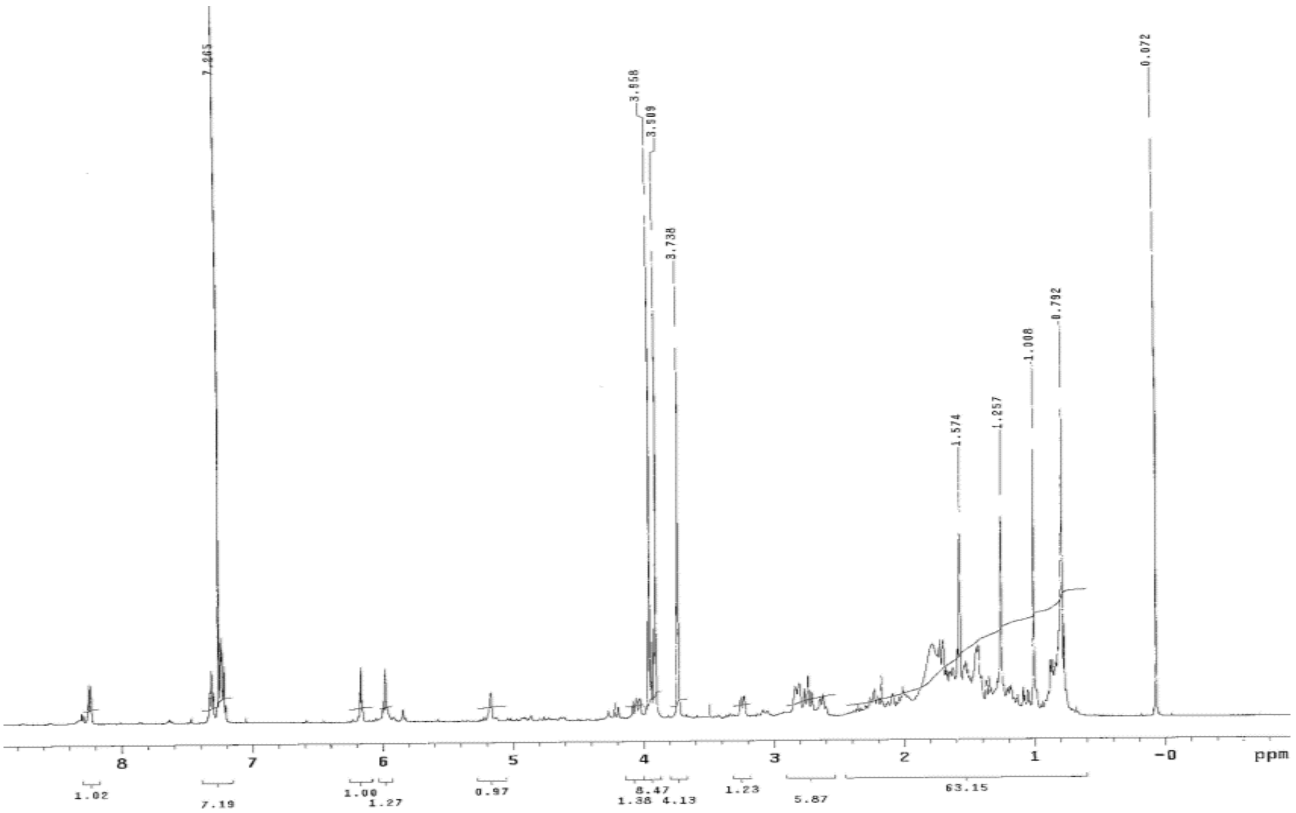

Figure PB-6. ^{1}H-NMR spectrum of polyalongarin B (**2**) in $CDCl_3$ (500 MHz)

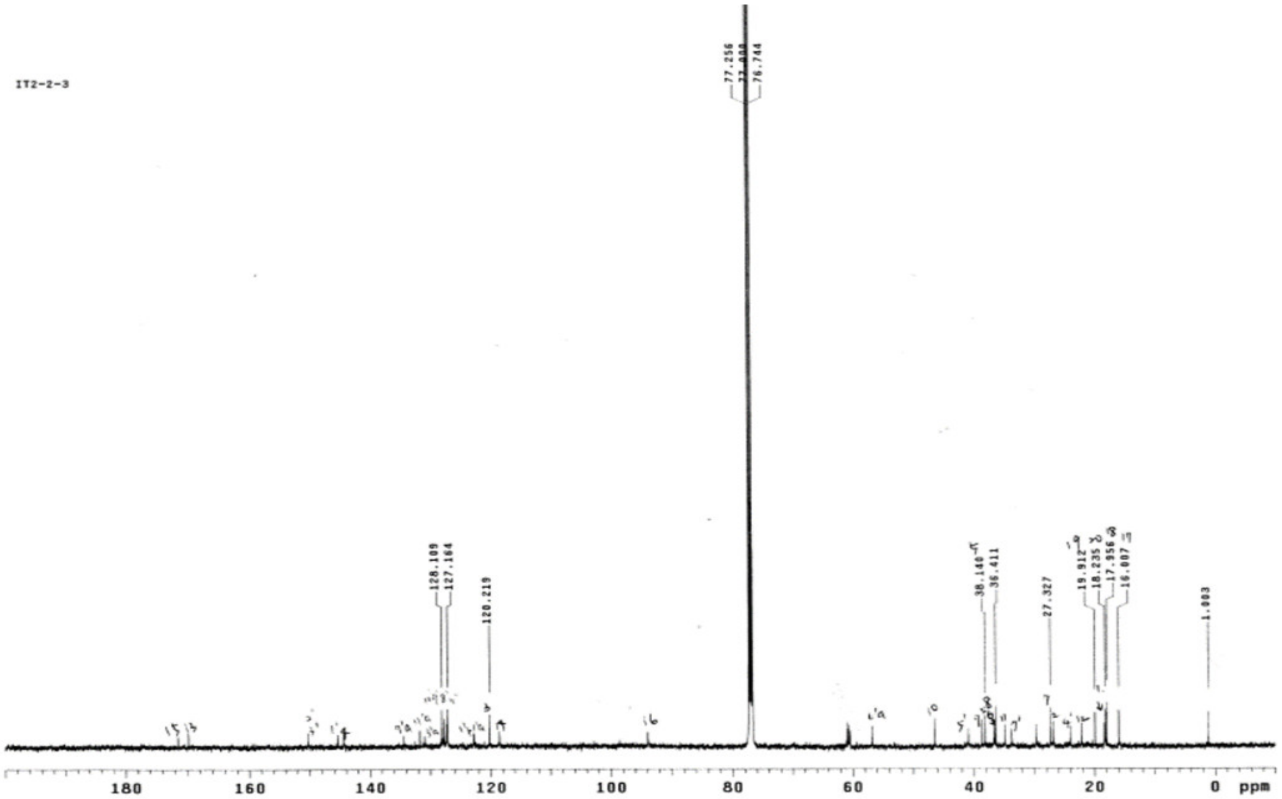

Figure PB-7. ^{13}C-NMR spectrum of polyalongarin B (**2**) in $CDCl_3$ (125 MHz)

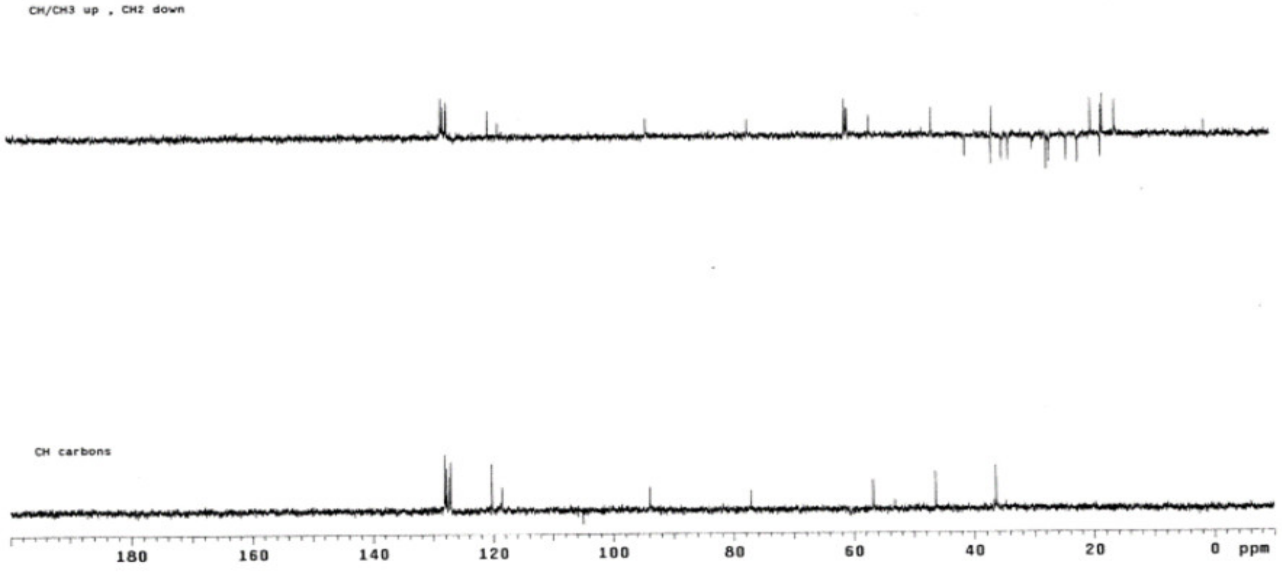

Figure PB-8. DEPT-135 and DEPT-90 spectra of polyalongarin B (**2**)

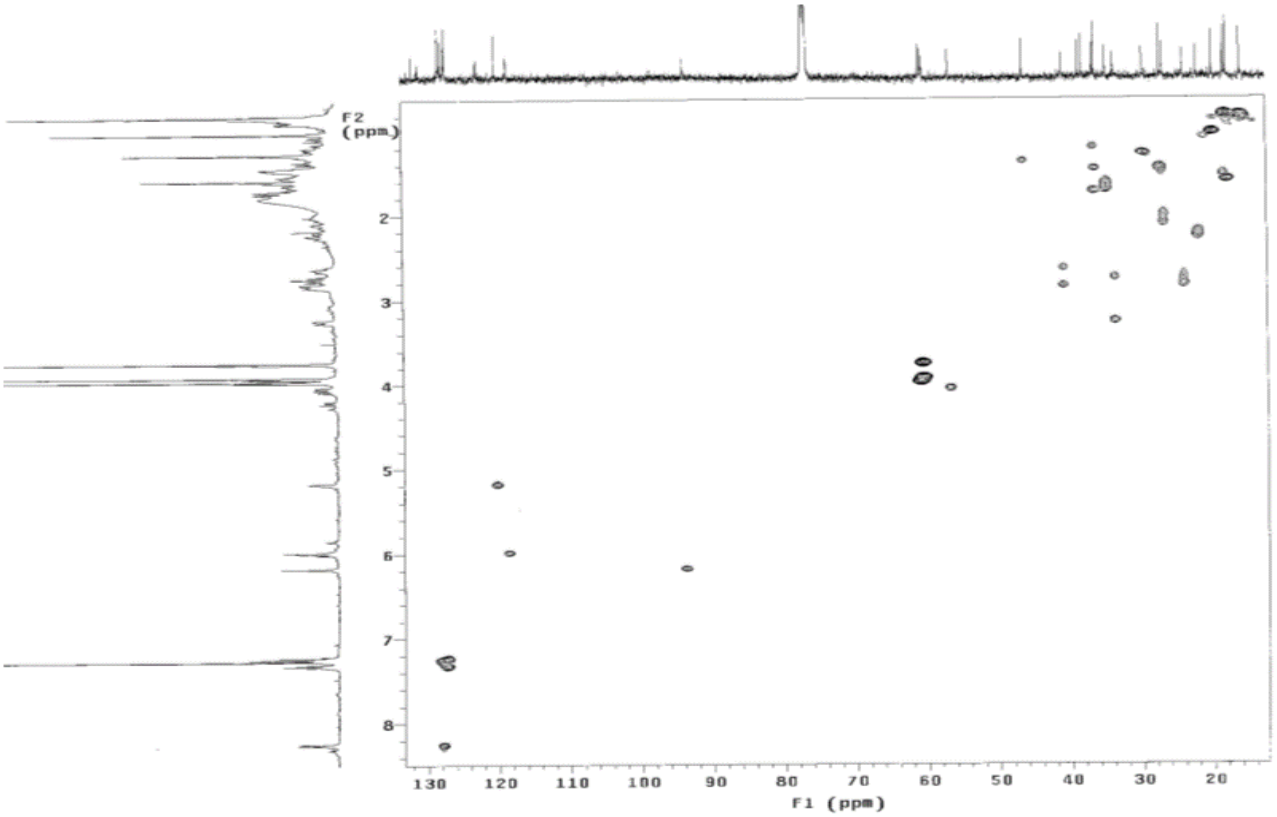

Figure PB-9. HMQC spectrum of polyalongarin B (**2**)

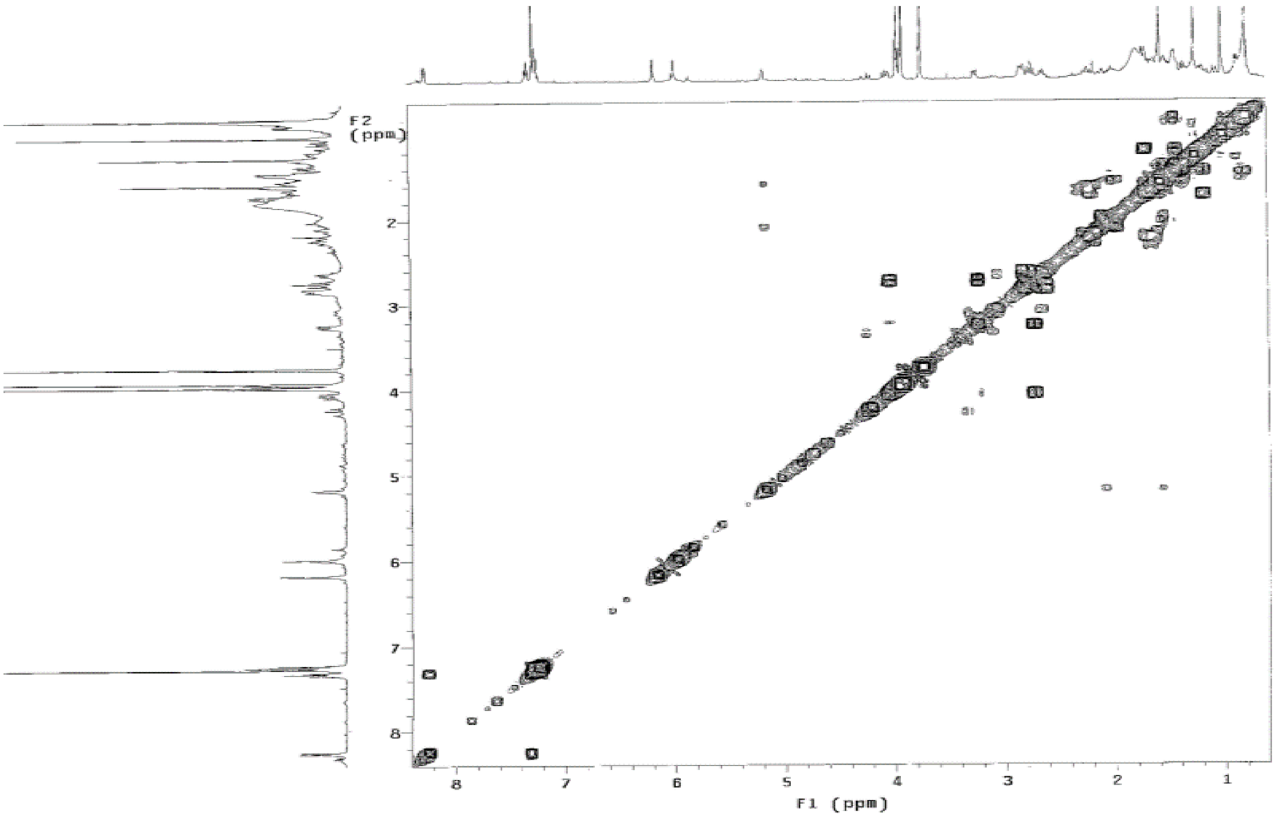

Figure PB-10. COSY spectrum of polyalongarin B (**2**)

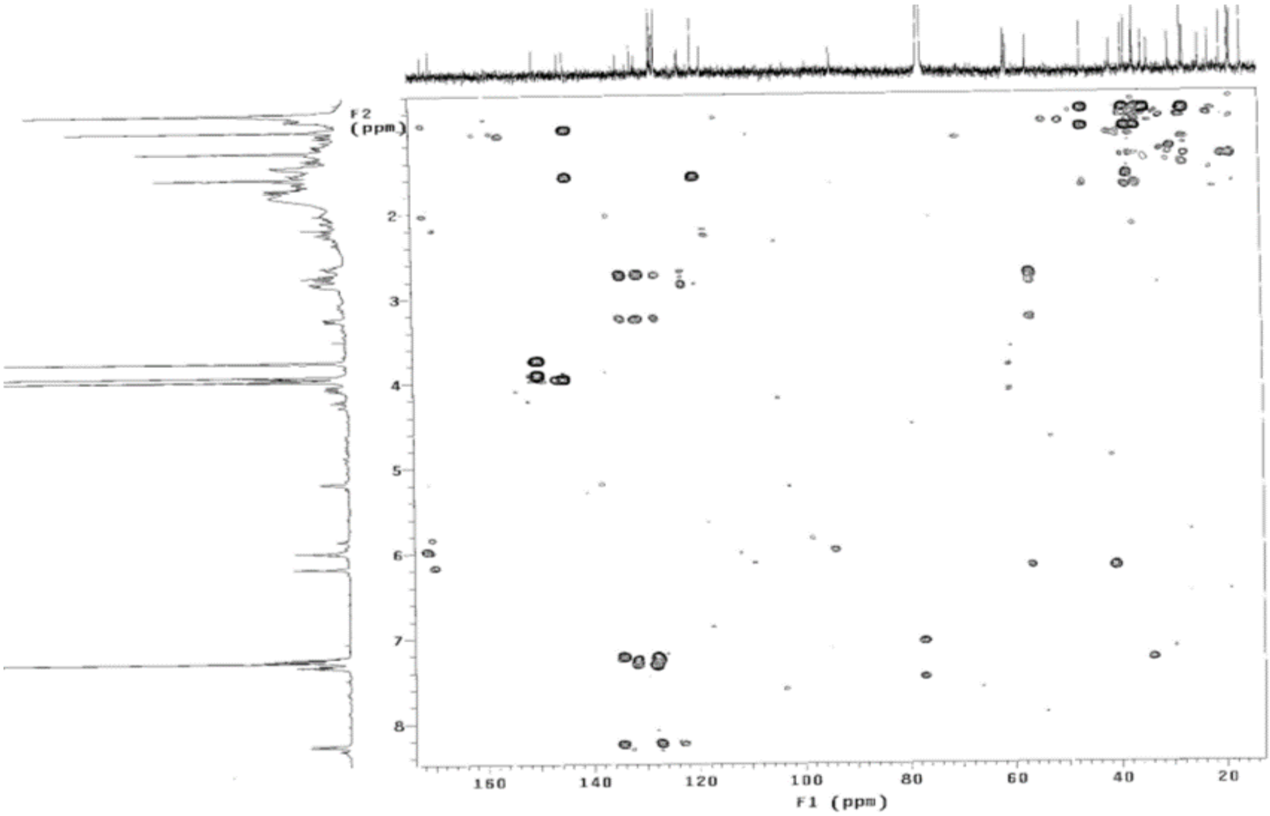

Figure PB-11. HMBC spectrum of polyalongarin B (**2**)

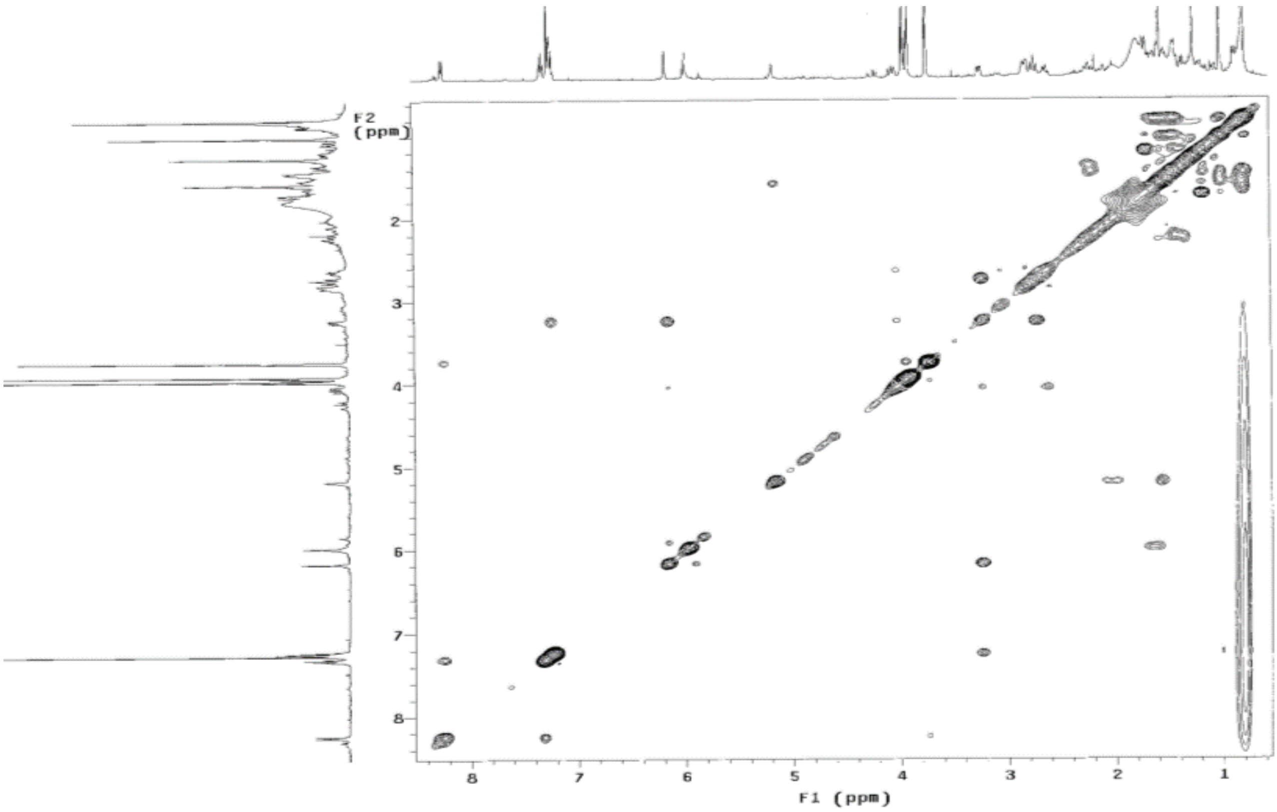

Figure PB-12. NOESY spectrum of polyalongarin B (**2**)

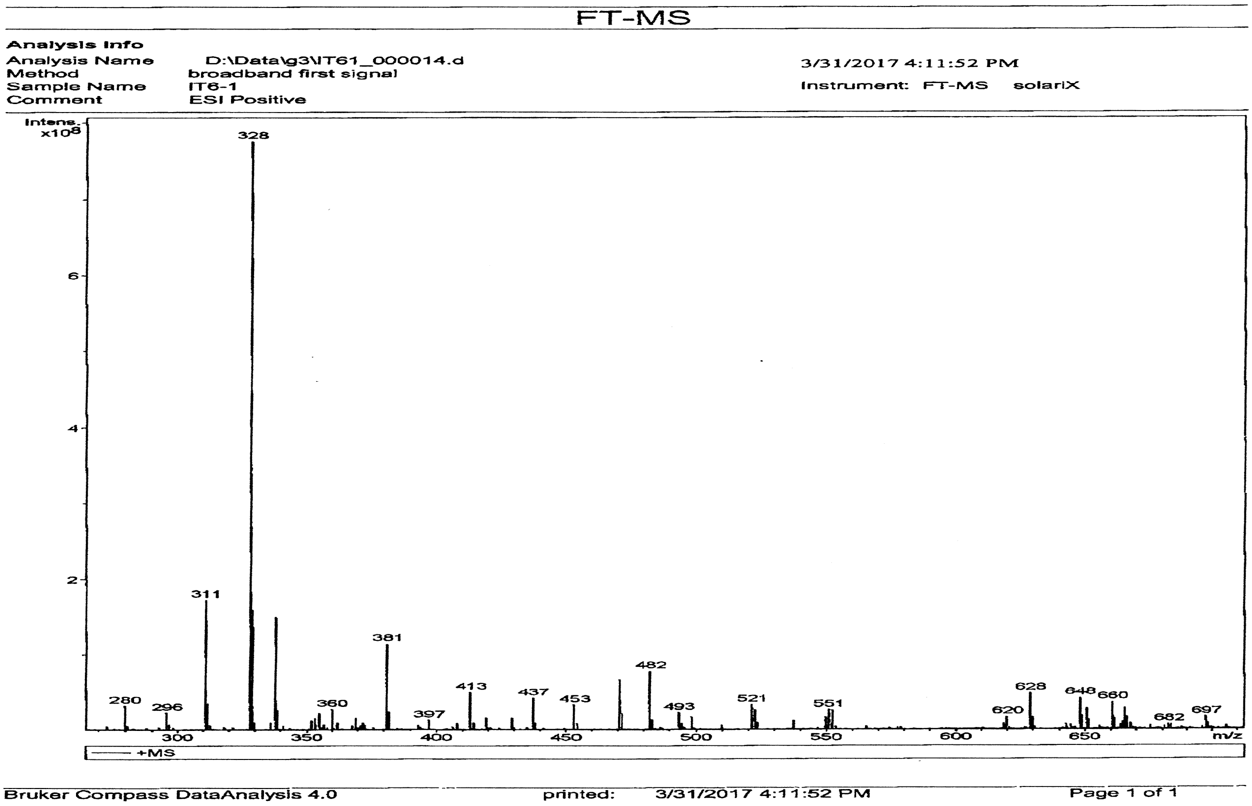

Figure PC-1. ESI-MS spectrum of polyalongarin C (**3**)

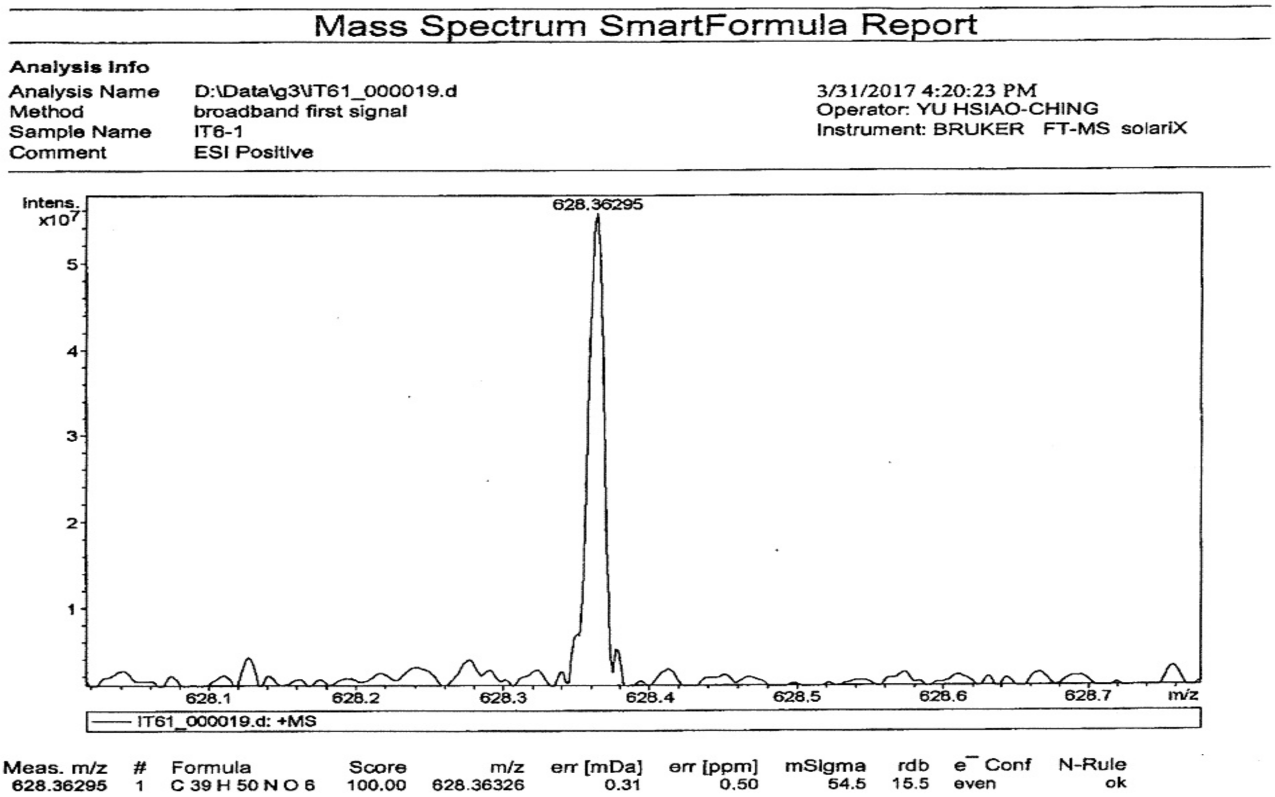

Figure PC-2. HR-ESI-MS spectrum of polyalongarin C (**3**)

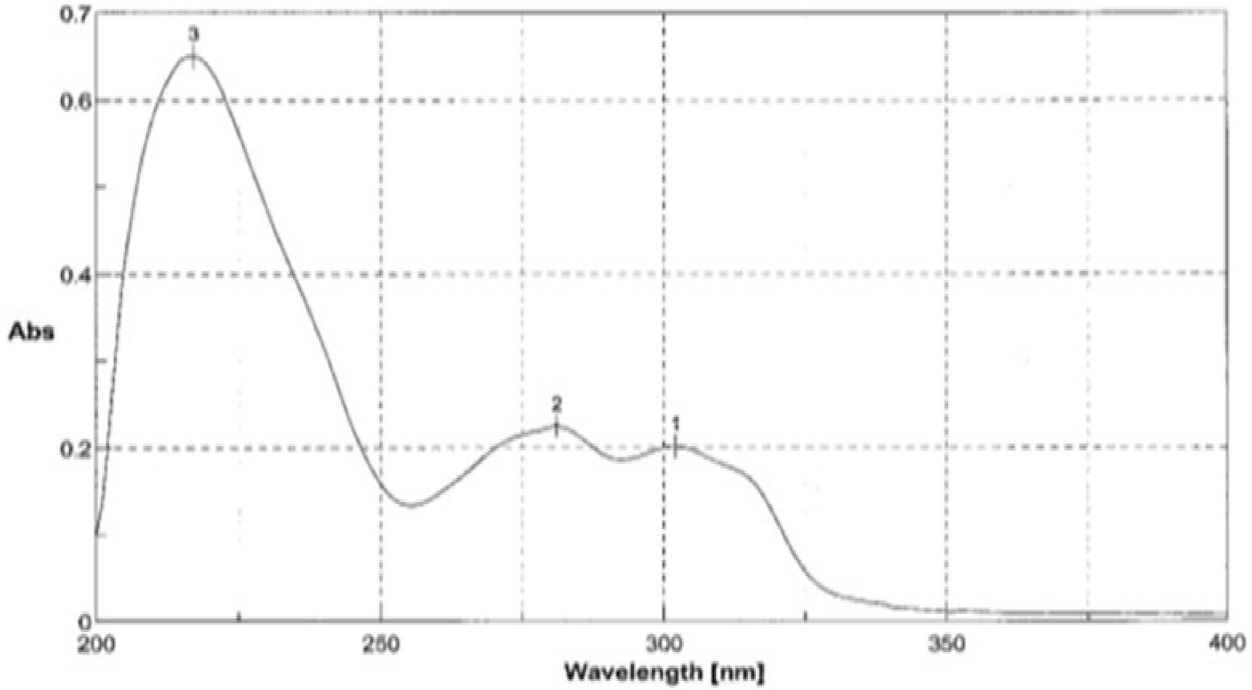

No.	nm	Abs	No.	nm	Abs	No.	nm	Abs
1	302	0.20191	2	281	0.22443	3	217	0.65022

Figure PC-3. UV spectrum of polyalongarin C (**3**)

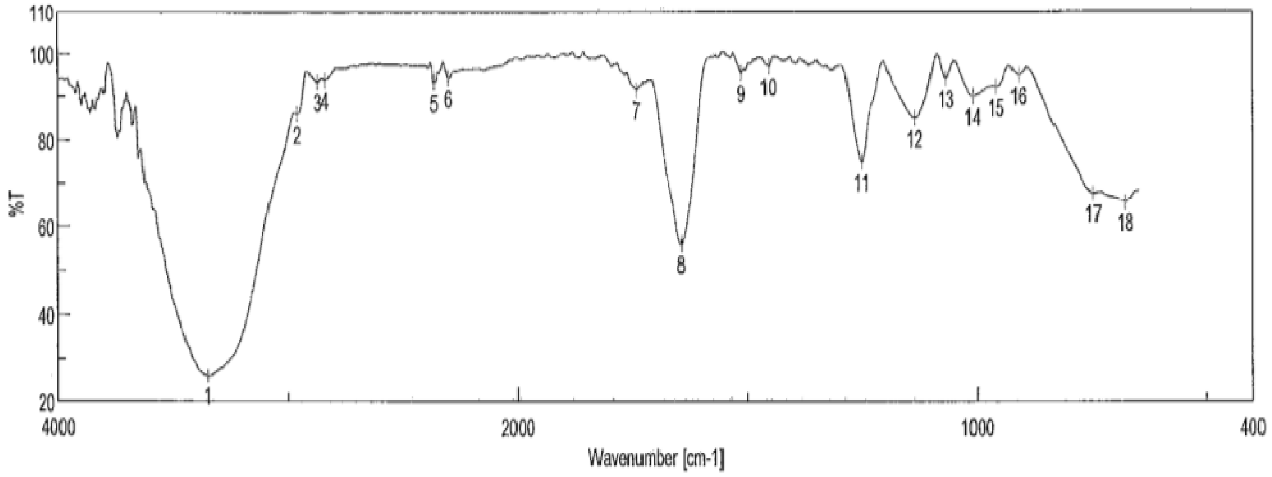

[Result of Peak Picking]

No.	Position	Intensity	No.	Position	Intensity	No.	Position	Intensity
1	3349.75	26.153	2	2959.23	85.9833	3	2871.49	93.3423
4	2838.7	93.7338	5	2362.37	93.312	6	2301.63	94.3505
7	1740.44	91.7453	8	1644.02	56.1846	9	1513.85	95.6288
10	1454.06	97.2024	11	1251.58	75.2242	12	1135.87	85.2725
13	1068.37	94.4238	14	1008.59	90.2655	15	958.448	92.4517
16	909.272	95.1685	17	746.317	67.8551	18	675.928	65.7945

Figure PC-5. IR spectrum of polyalongarin C (**3**)

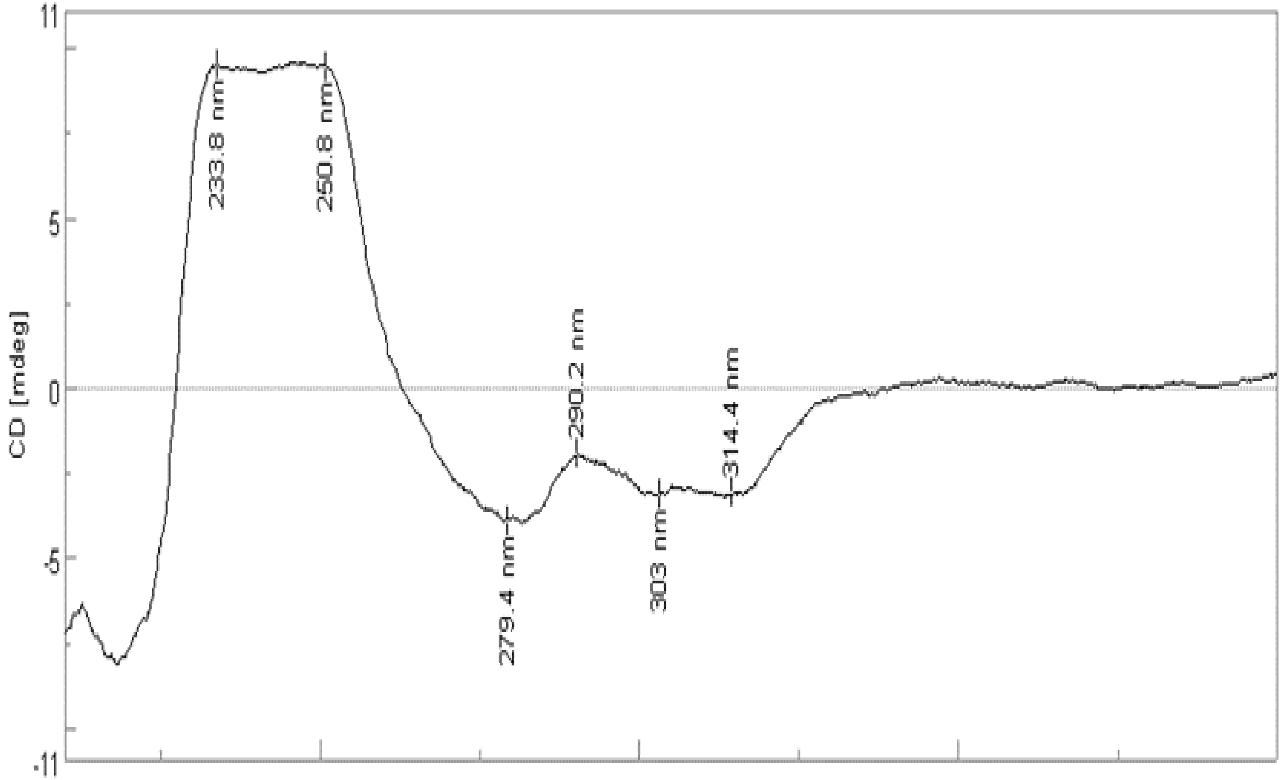

Figure PC-5. CD spectrum of polyalongarin C (**3**)

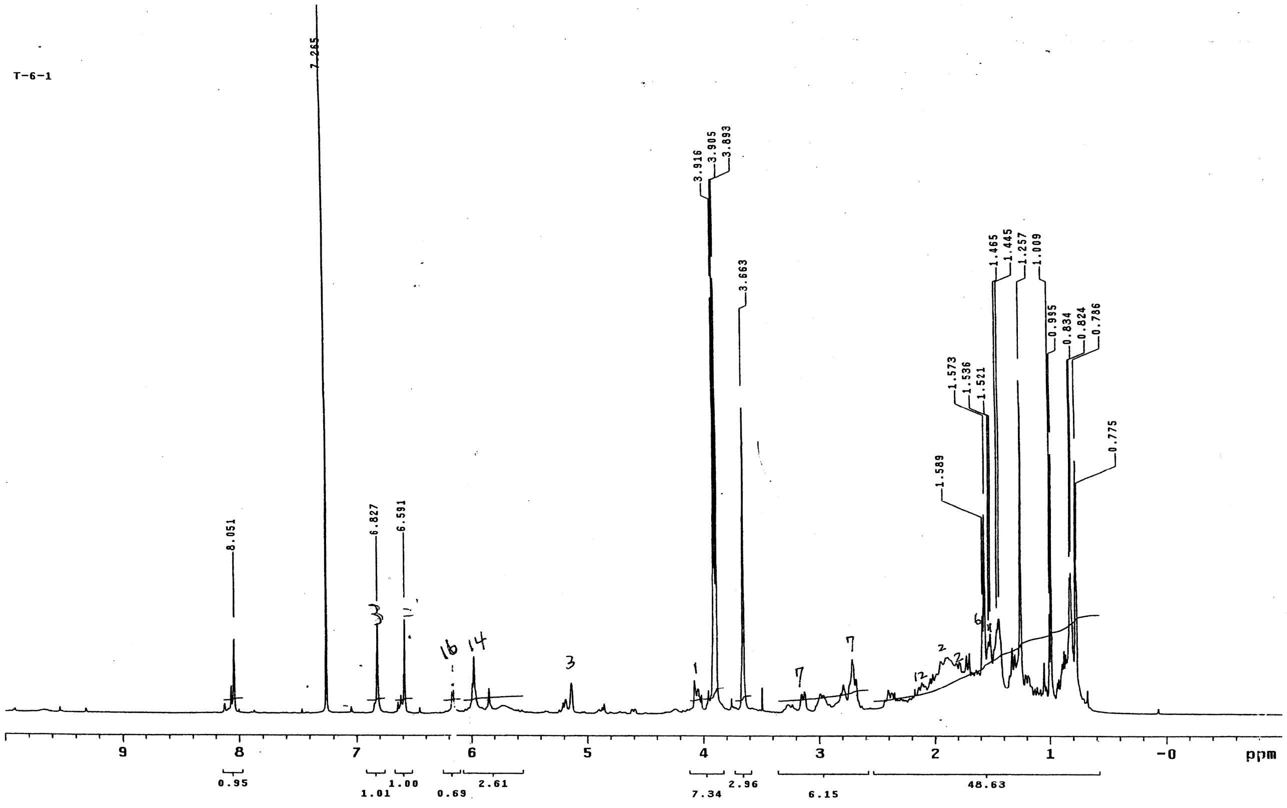

Figure PC-6. ^{1}H-NMR spectrum of polyalongarin C (**3**) in $CDCl_3$ (500 MHz)

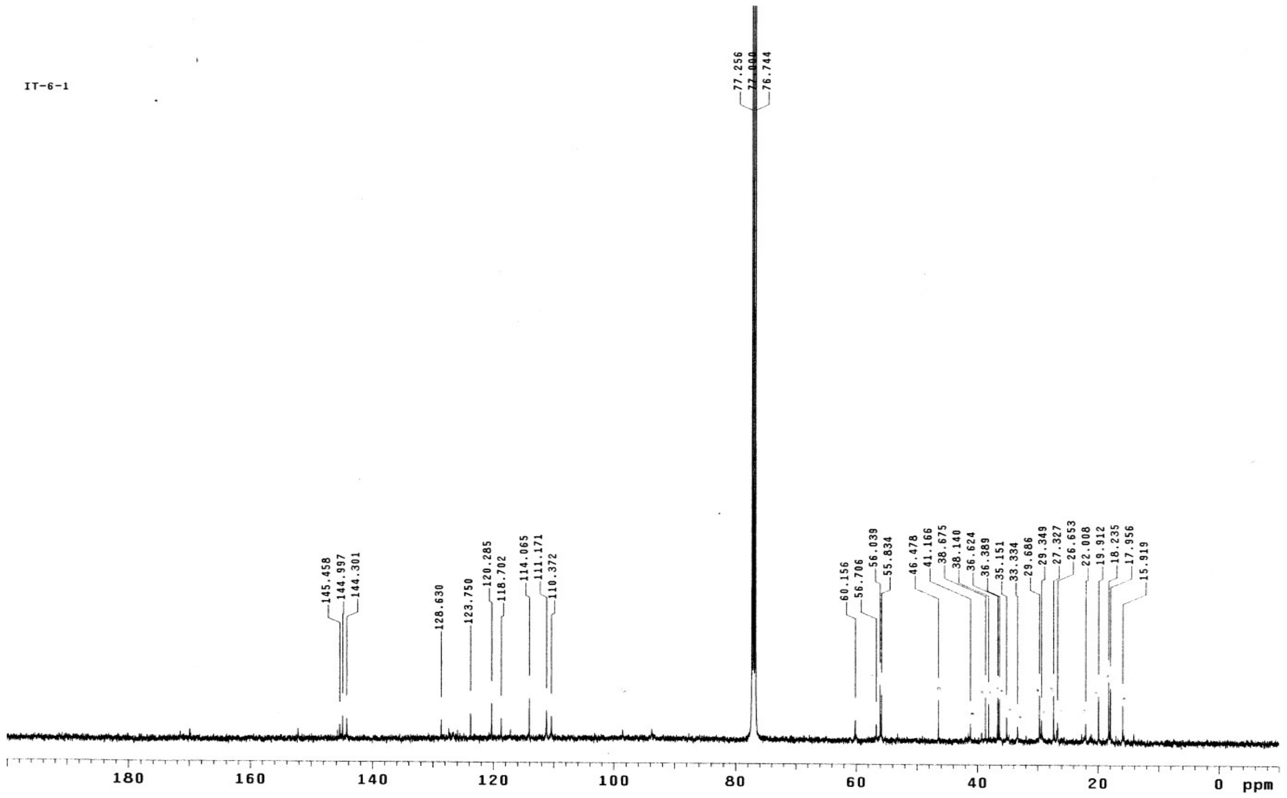

Figure PC-7. ^{13}C-NMR spectrum of polyalongarin C (**3**) in $CDCl_3$ (125 MHz)

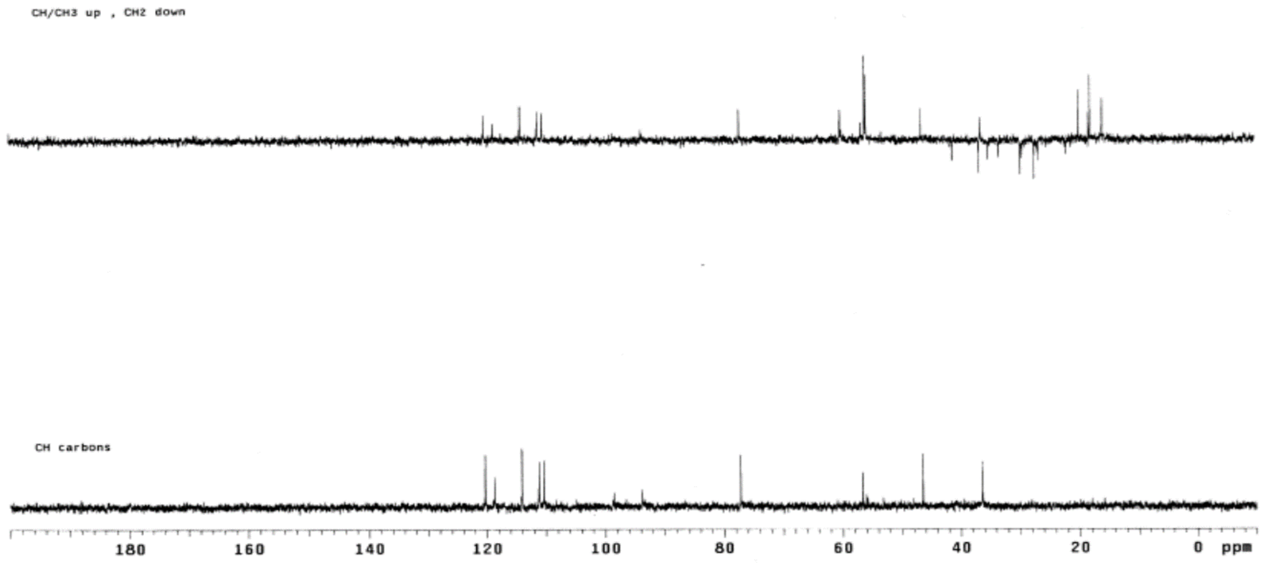

Figure PC-8. DEPT-135 and DEPT-90 spectra of polyalongarin C (**3**)

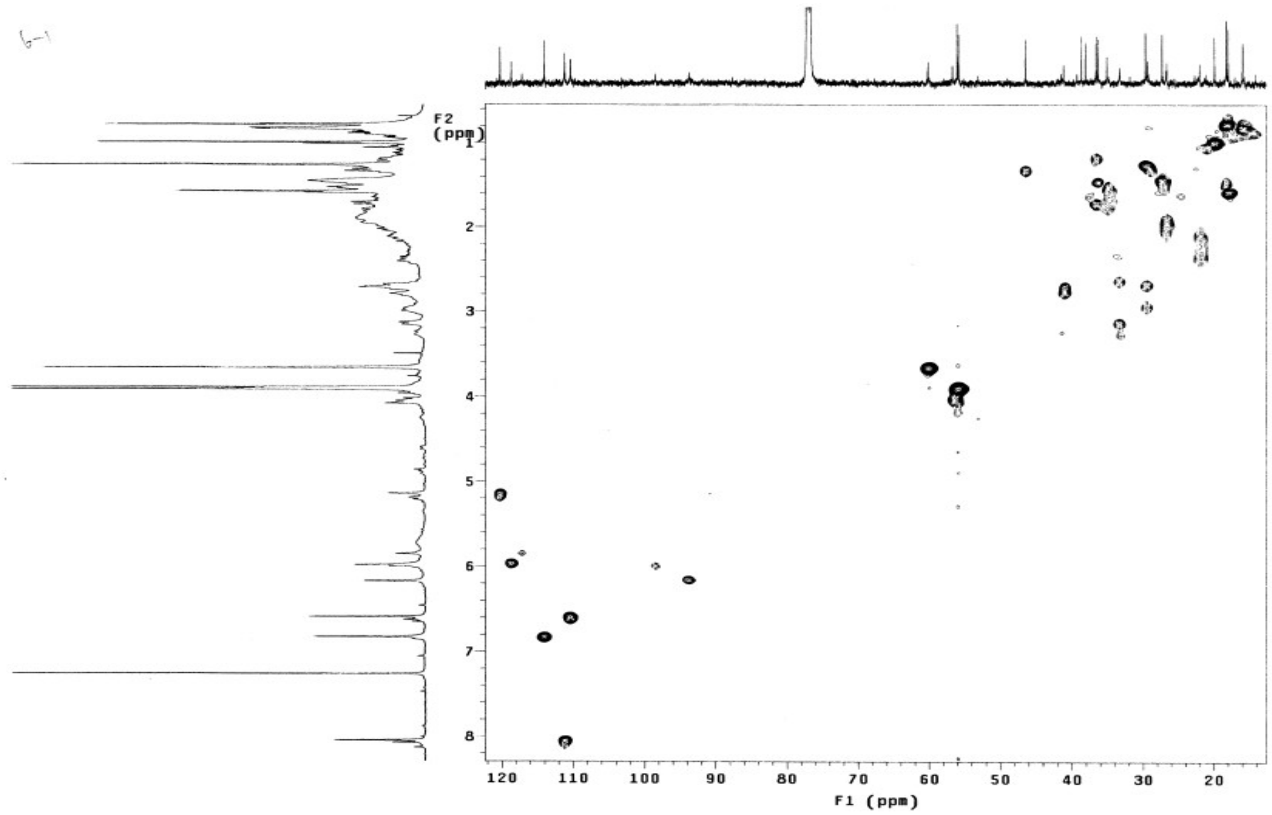

Figure PC-9. HMQC spectrum of polyalongarin C (**3**)

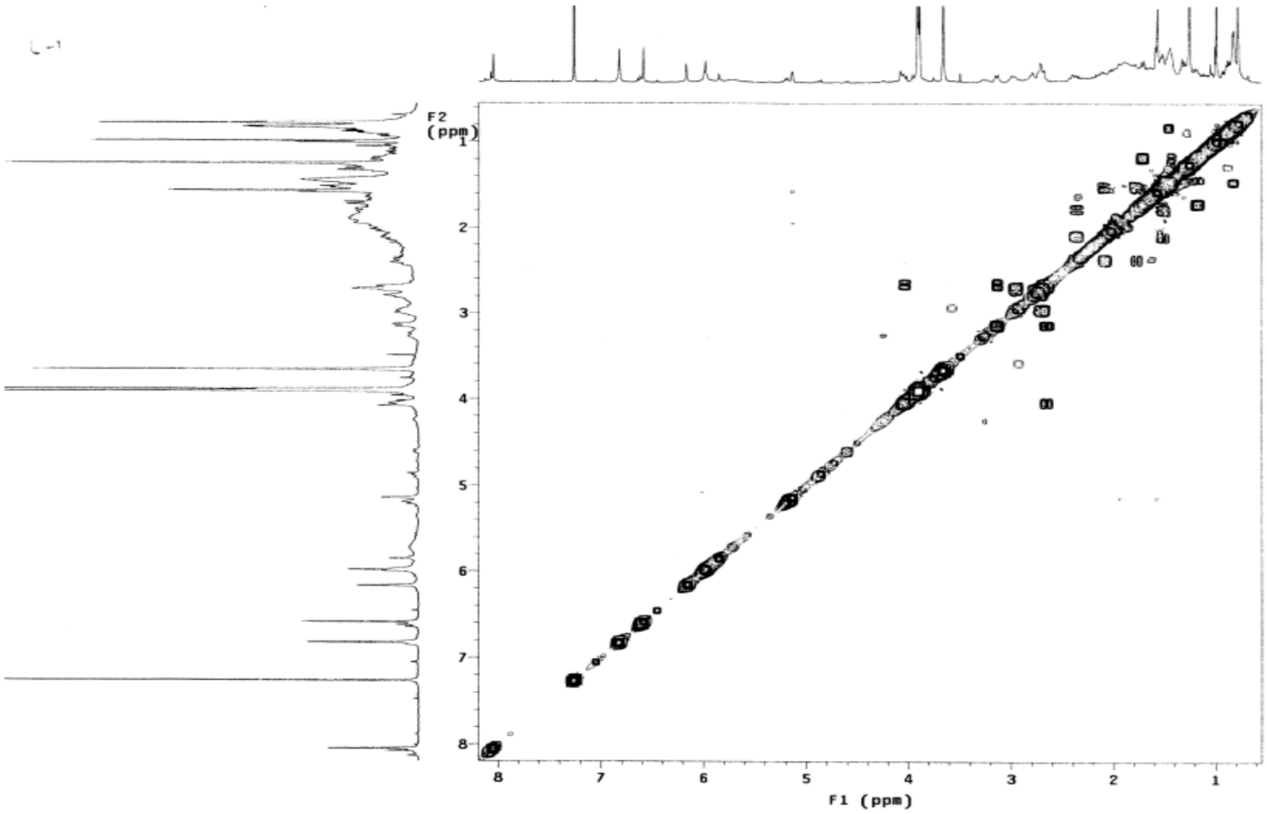

Figure PC-10. COSY spectrum of polyalongarin C (**3**)

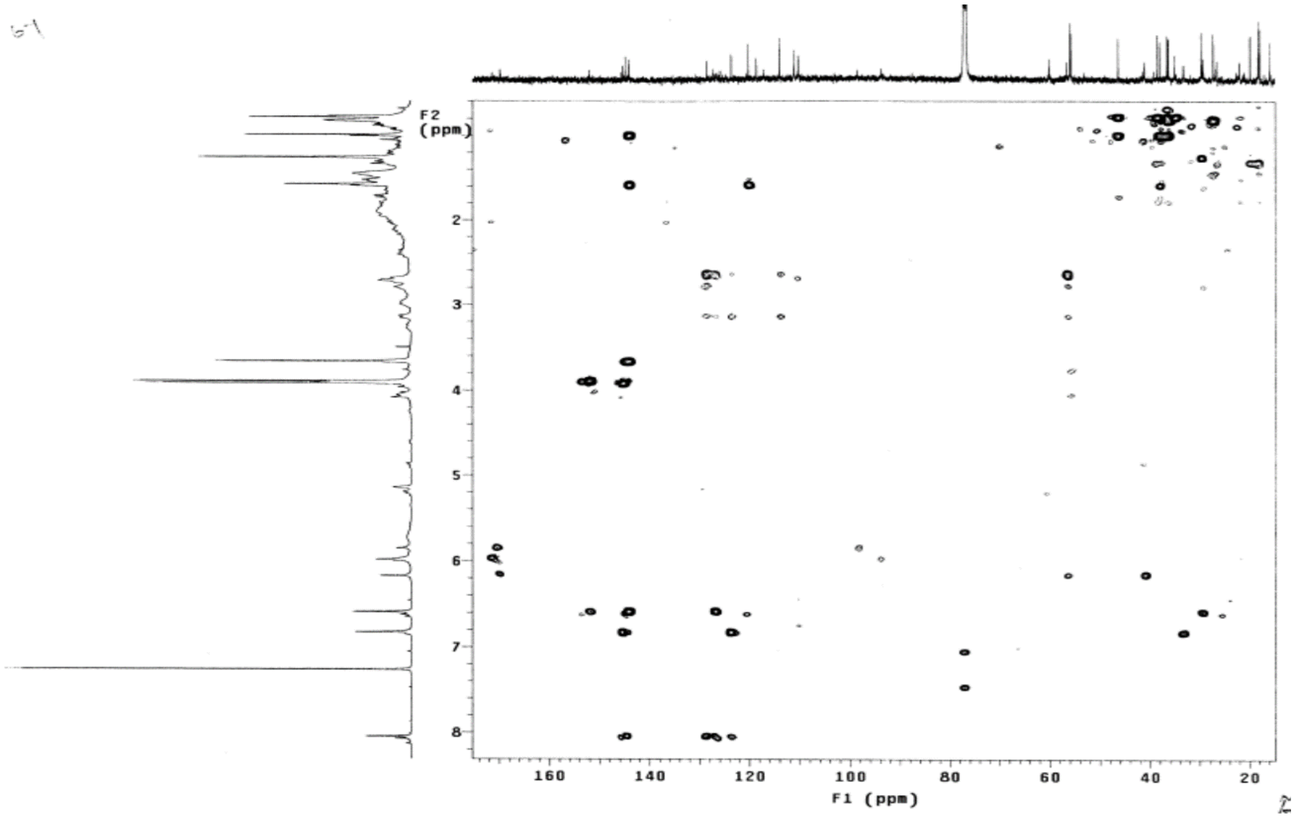

Figure PC-11. HMBC spectrum of polyalongarin C (**3**)

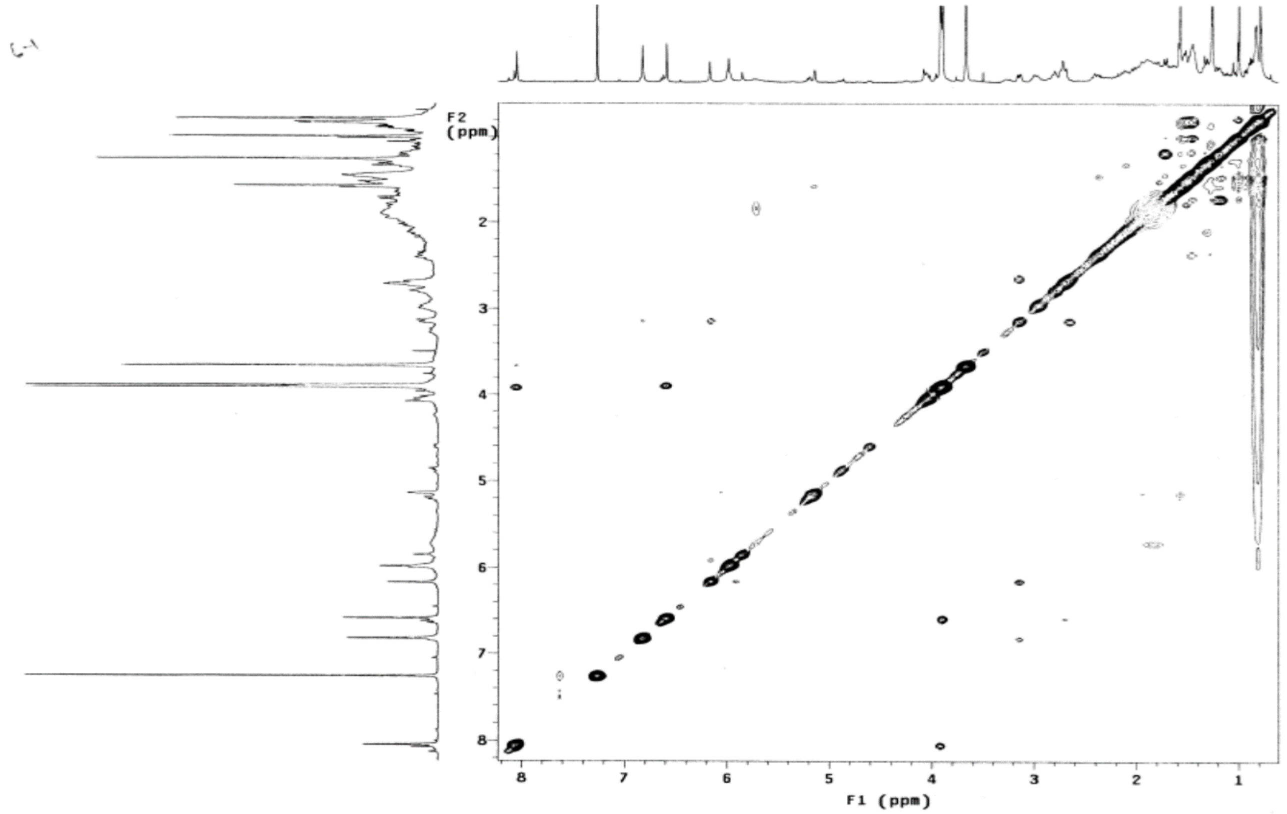

Figure PC-12. NOESY spectrum of polyalongarin C (**3**)

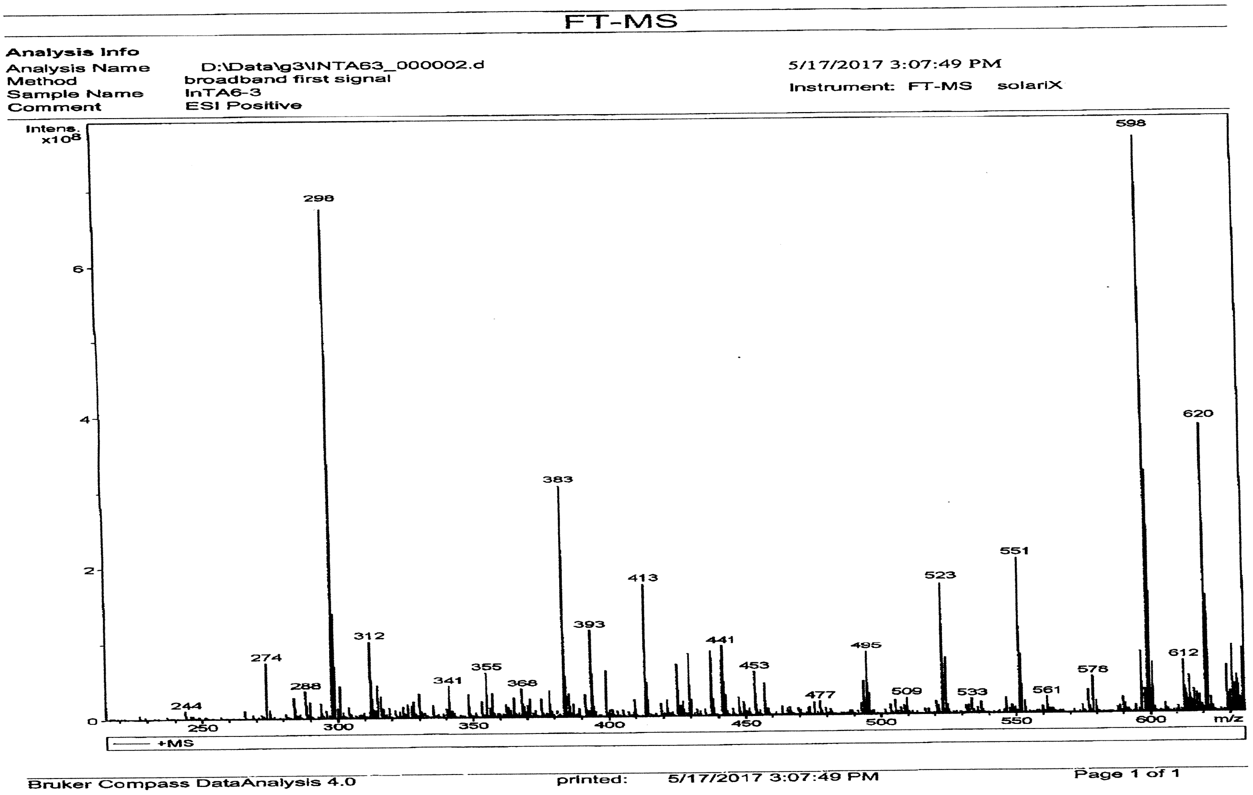

Figure PD-1. ESI-MS spectrum of polyalongarin D (**4**)

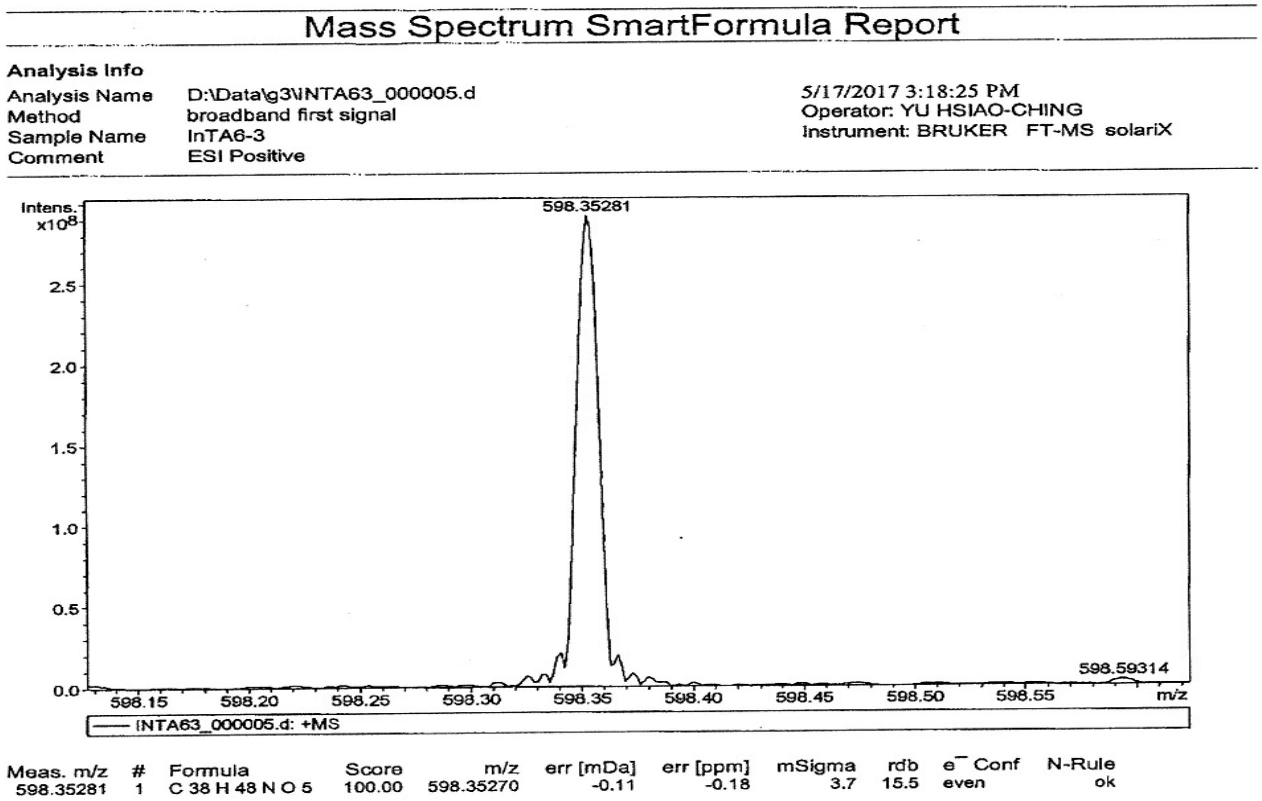

Figure PD-2. HR-ESI-MS spectrum of polyalongarin D (**4**)

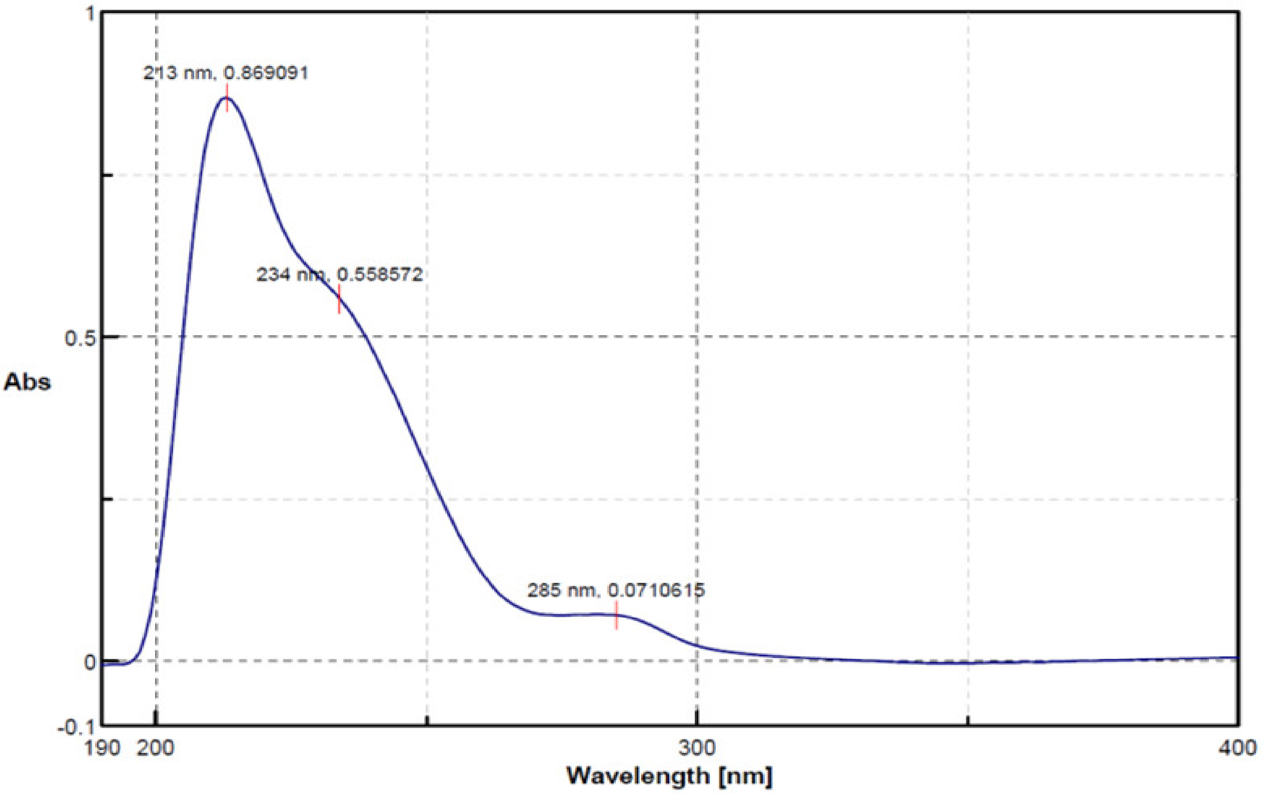

Figure PD-3. UV spectrum of polyalongarin D (**4**)

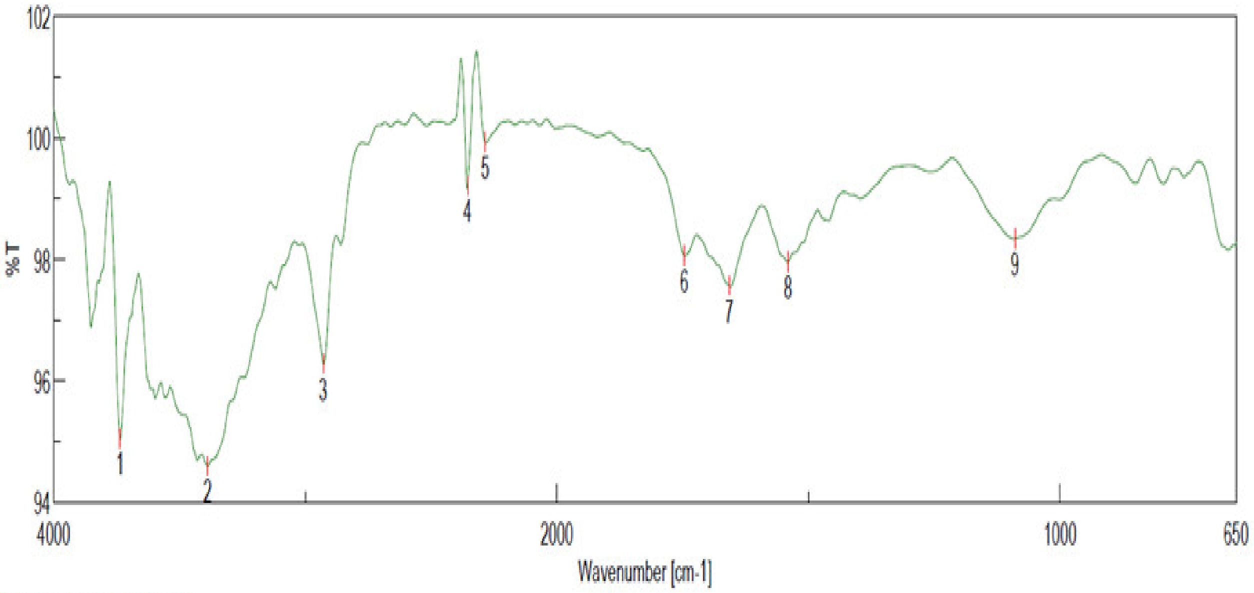

[Result of Peak Picking]

No.	Position	Intensity	No.	Position	Intensity	No.	Position	Intensity
1	3736.4	95.0491	2	3388.32	94.5968	3	2929.34	96.2745
4	2355.62	99.205	5	2285.23	99.9143	6	1747.19	98.0573
7	1657.52	97.5618	8	1540.85	97.9485	9	1089.58	98.337

Figure PD-4. IR spectrum of polyalongarin D (**4**)

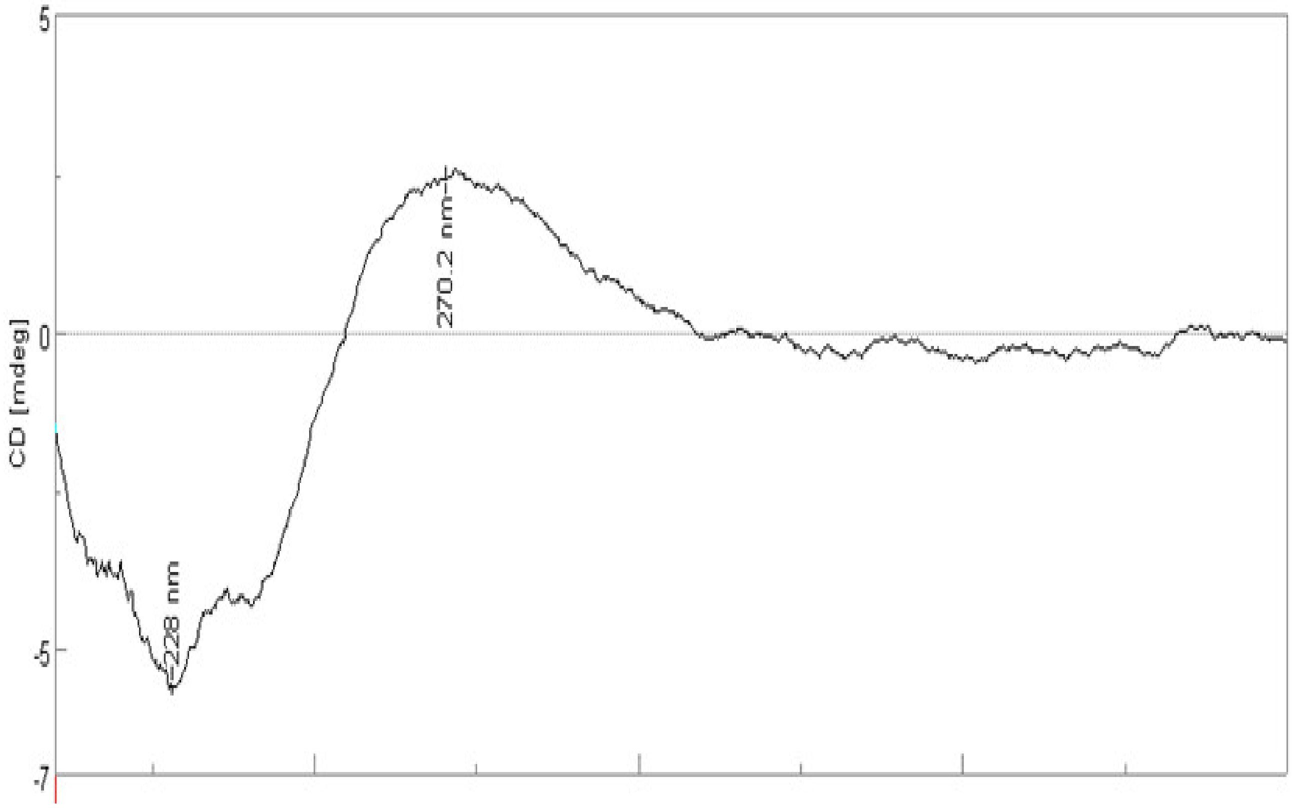

Figure PD-5. CD spectrum of polyalongarin D (**4**)

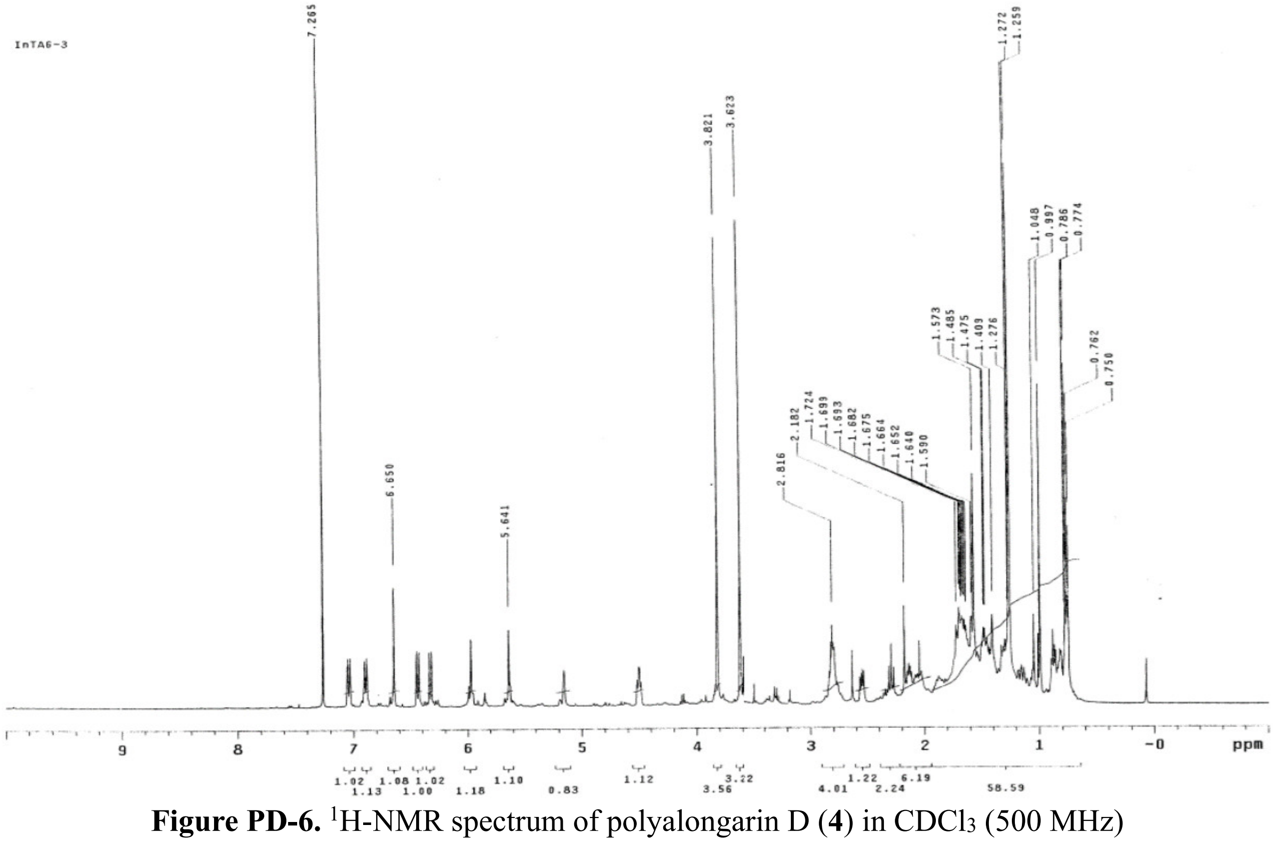

Figure PD-6. ^{1}H-NMR spectrum of polyalongarin D (**4**) in $CDCl_3$ (500 MHz)

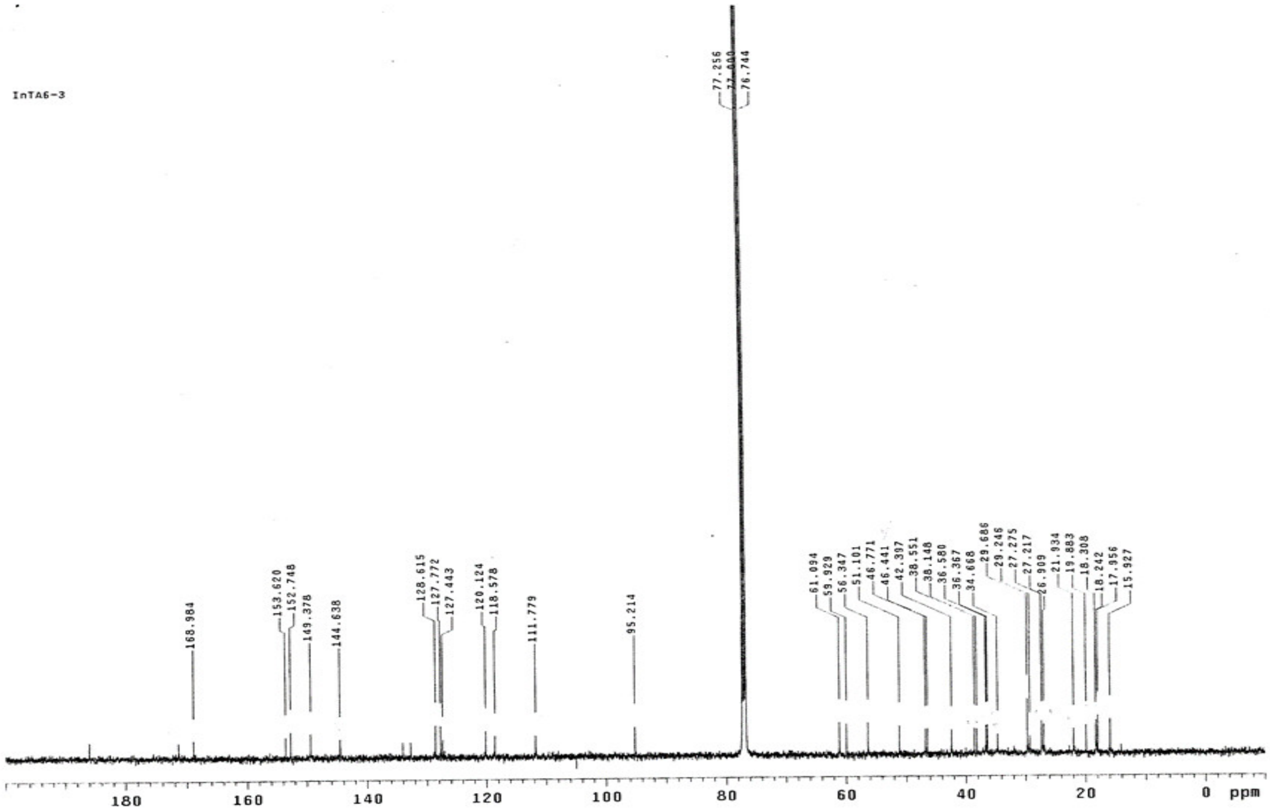

Figure PD-7. ^{13}C-NMR spectrum of polyalongarin D (**4**) in $CDCl_3$ (125 MHz)

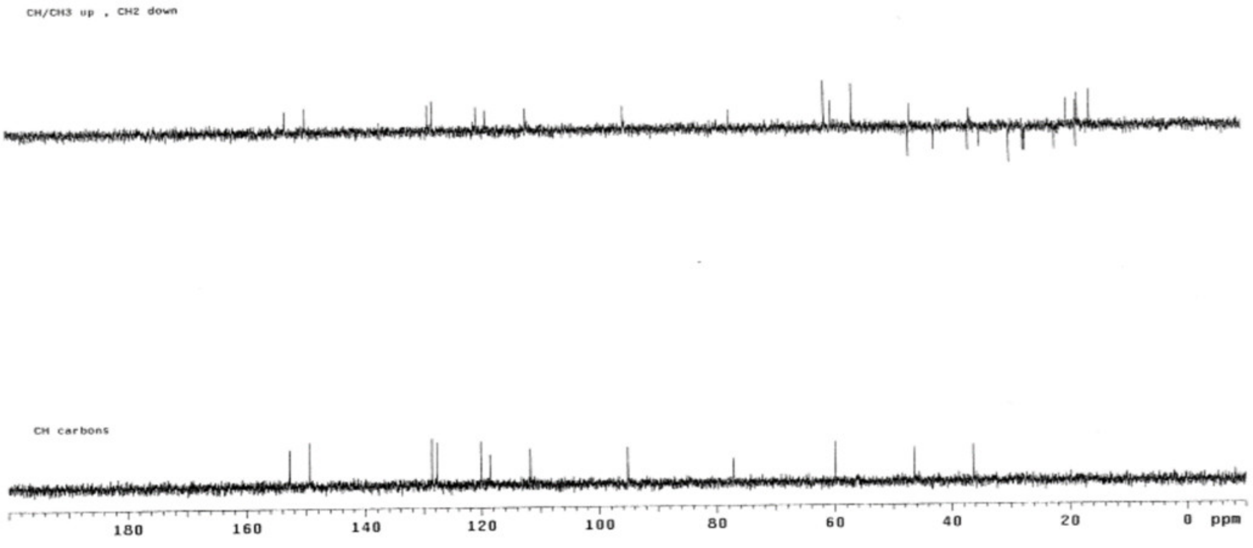

Figure PD-8. DEPT-135 and DEPT-90 spectra of polyalongarin D (**4**)

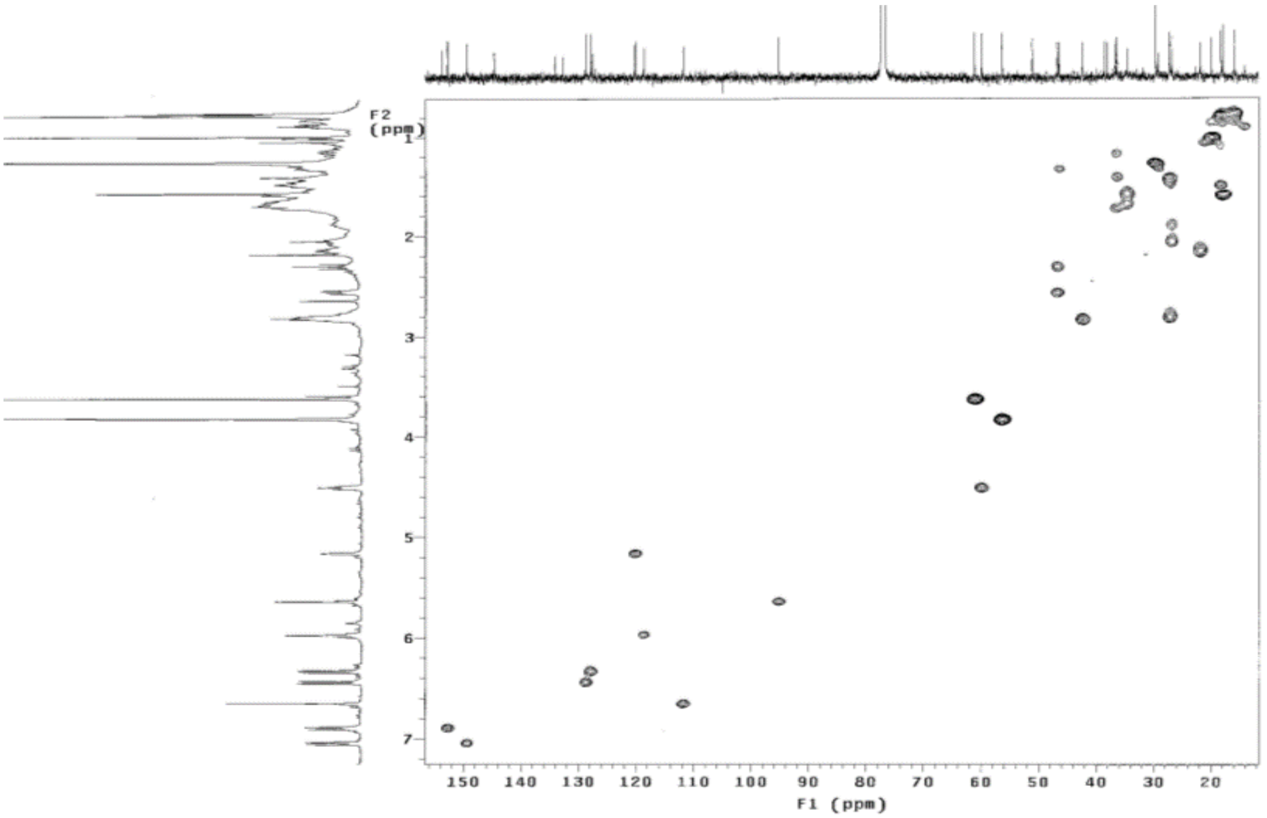

Figure PD-9. HMQC spectrum of polyalongarin D (**4**)

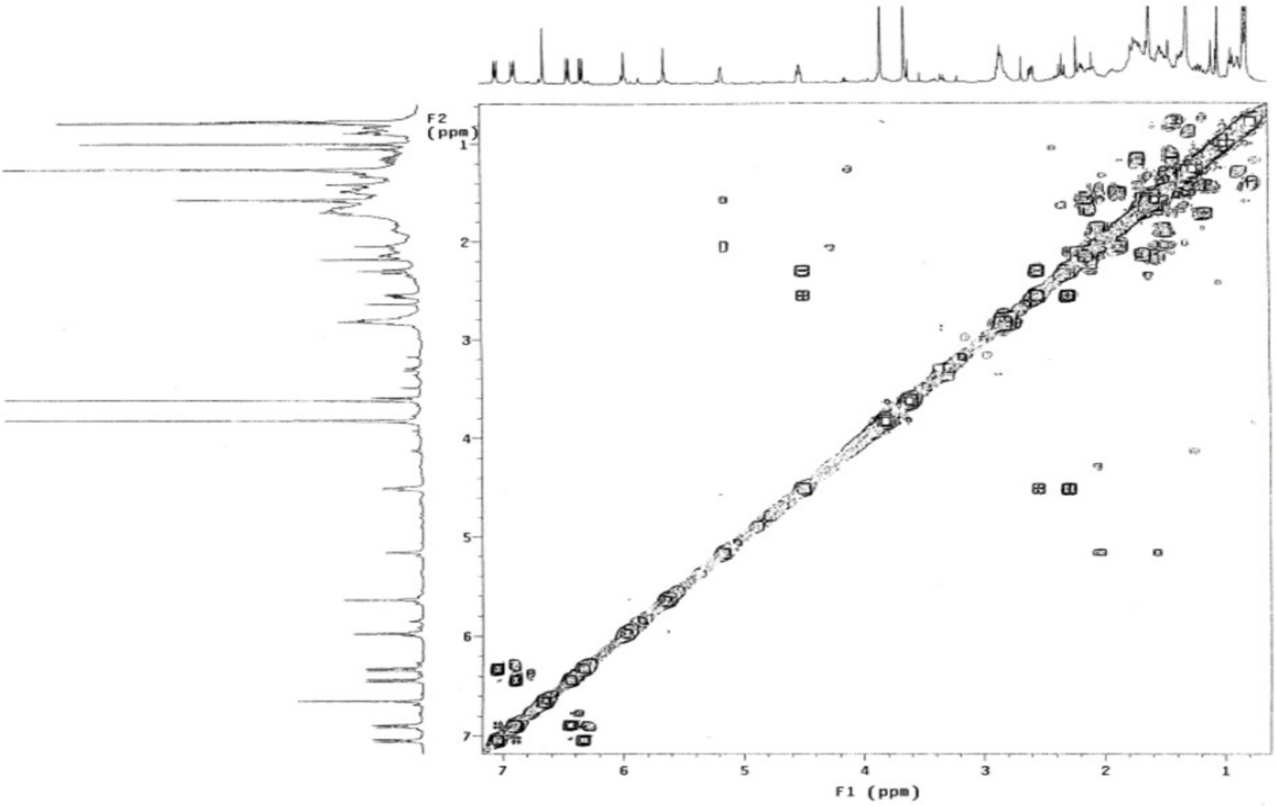

Figure PD-10. COSY spectrum of polyalongarin D (**4**)

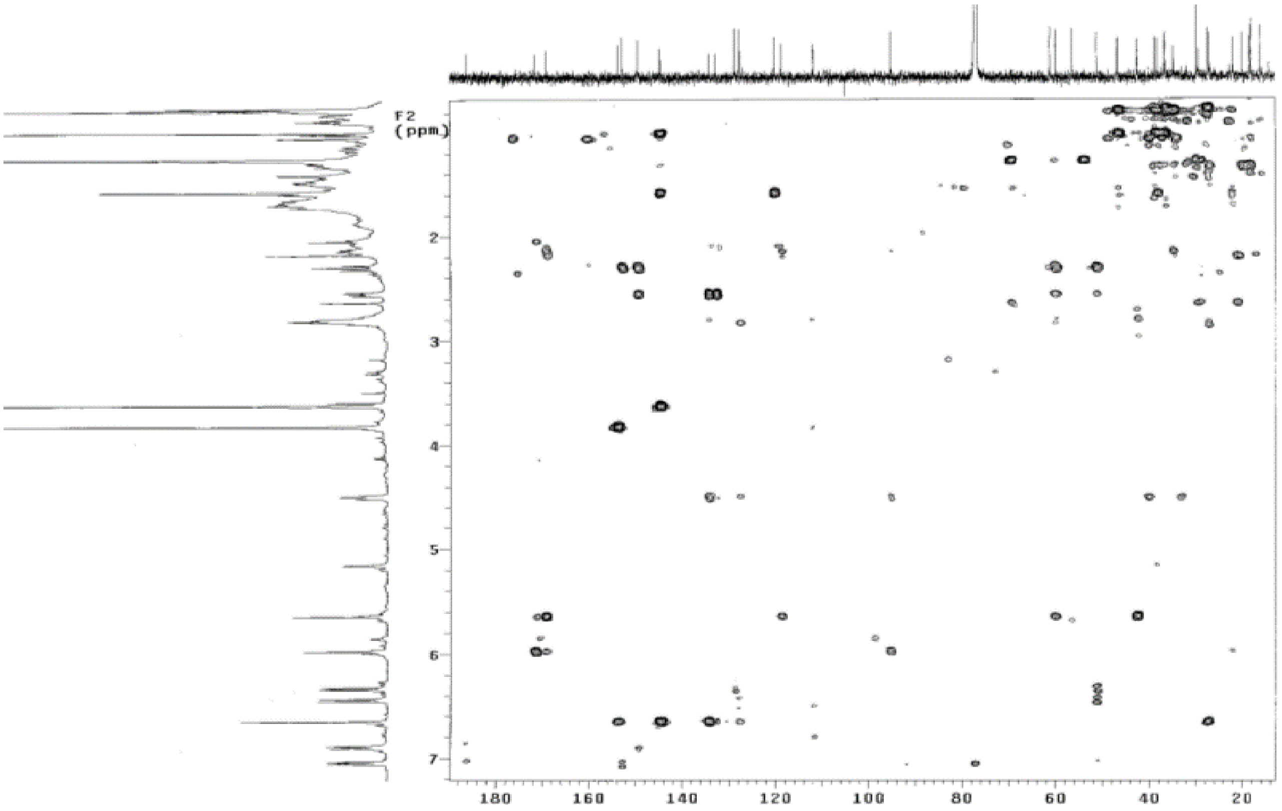

Figure PD-11. HMBC spectrum of polyalongarin D (**4**)

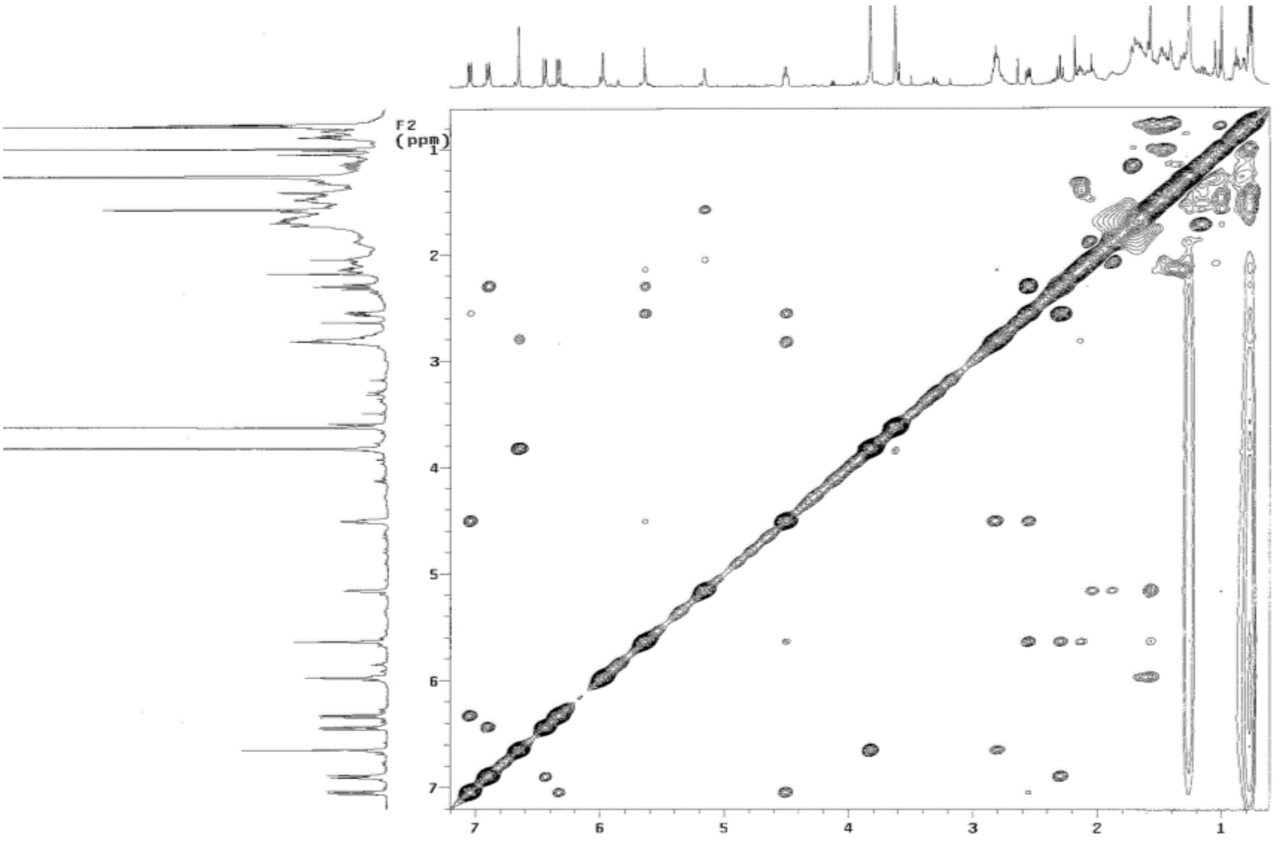

Figure PD-12. NOESY spectrum of polyalongarin D (**4**)
